# Red Wine Inspired Chemistry: Hemisynthesis of Procyanidin Analogs and Determination of Their Protein Precipitation Capacity, Octanol–Water Partition, and Stability in Phosphate-Buffered Saline

**DOI:** 10.1021/acs.jafc.3c06467

**Published:** 2023-12-04

**Authors:** Juuso Erik Laitila, Petri Tapani Tähtinen, Maarit Karonen, Juha-Pekka Salminen

**Affiliations:** a Department of Chemistry, University of Turku, Turku, FI-20014, Finland

**Keywords:** bioactivity, catechin, log *P*, PBS, proanthocyanidin, protein interaction, structure−activity, tannin

## Abstract

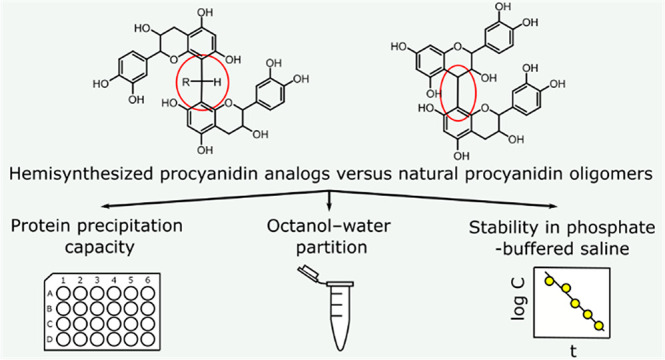

Ten dimeric procyanidin (PC) analogs were hemisynthesized from catechin or epicatechin and from five different aldehydes using the same mechanism that produces the important acetaldehyde-mediated adducts of proanthocyanidins (PAs) and anthocyanins in red wine. Protein precipitation capacity (PPC), octanol–water partition coefficient (log *P*) and stability of the PC analogs were determined. The emphasis was on the PPC because it has been shown to correlate with anthelmintic activity against gastrointestinal nematodes in ruminants and with other beneficial bioactivities in animals, as well. The PPC of PC analogs was greatly improved compared to natural PC dimers, but the capacity was not as great as that of a PC trimer or epigallocatechin gallate. The log *P* of PC analogs varied from hydrophobic to hydrophilic depending on the intramolecular linkage. Great variation was observed in stabilities of PC analogs in phosphate buffered saline, and the mixtures of degradation products were characterized using high-resolution mass spectrometry.

## 1 Introduction

**Figure 1:**
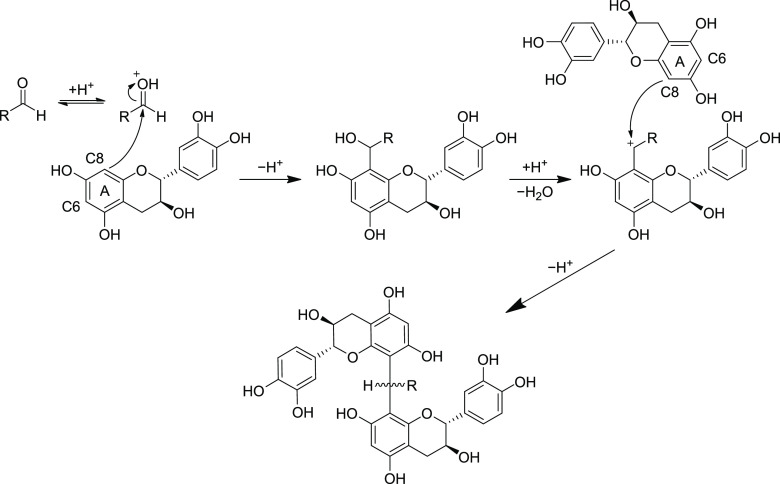
Oligomerization mechanism of (epi)catechins via an aldehyde by two subsequent electrophilic aromatic substitutions. The mechanism is the same as the one that produces methylmethine-linked adducts of proanthocyanidins and anthocyanins in red wine.^4^

The chemical reactions that occur in red wine demonstrate how reactive certain natural products can be under the right conditions. This reactivity plays a major role in the development and stability of the color of red wine because two of the reactive polyphenol groups in red wine are the anthocyanins and proanthocyanidins (PA), which together form the important PA–anthocyanin adducts.^1−3^ Anthocyanins and PAs, as well as the monomeric building units of PAs, e.g., (epi)catechins, all have a common structural moiety, which is their A-ring. This ring contains three *o*/*p* activators that make the C6 and C8 carbons nucleophilic, which in turn enables the development of the wine color by formation of certain types of PA–anthocyanin adducts.^4^ One way for PAs and anthocyanins to form adducts is via an aldehyde by two subsequent electrophilic aromatic substitutions.^3,4^ Naturally, this reaction can occur between (epi)catechins as well (Figure 1) and such products are known to form in wine-like model solutions.^5−7^

The condensation reactions between (epi)catechins and anthocyanins have been extensively studied in model solutions, but their context has mostly been red wine and how the formed products affect the properties of red wine, such as color.^8−12^ However, PAs have also been modified by similar mechanisms for other commercial applications. In recent years, PAs have been gaining attention as biobased starting materials to replace phenols in manufacturing of phenol–aldehyde resins, which are formed by similar mechanisms as PA–anthocyanin adducts in red wine.^13^ There is a growing interest toward PAs and other tannins as replacements for phenols as starting materials because of their lower costs, higher biodegradability and biocompatibility, and other enhanced properties compared to phenol-based resins.^13,14^ For instance, the resinous products of PAs and aldehydes have been studied for their applicability as adhesives, biobased foams, and as coating materials that provide resistance to water, fire, and corrosion.^13^ Importantly, however, PAs modified by reactions with aldehydes have only rarely been studied for their biological properties that natural PAs are known have.

PAs have multiple beneficial effects on human health such as anti-inflammatory, antioxidant, antimutagenic, and anticancer properties.^15^ The positive health effects are not limited only to humans but also to animals, such as ruminants as well. In goats, sheep, and cattle, the positive effects of PAs include antiparasitic effects against gastrointestinal parasites, improved growth and improved milk yields, and the global environment benefits from the reduced greenhouse gas emission caused by the ingested PAs.^16^ A key property of PAs behind their antiparasitic activity is thought to be their ability to bind to proteins, which happens via hydrophobic interactions, hydrogen bonding, and π–π stacking between PAs and proteins.^16,17^ In general, the capacity of PAs to bind with and precipitate proteins is largely explained by the degree of polymerization,^16,17^ albeit other factors play a role as well, such as three-dimensional structure and structural flexibility.^18,19^ In this context, the reactions between PAs and aldehydes provide means to potentially improve biological properties of PAs by artificially increasing the degree of oligomerization by linking PA units together, by incorporating potentially bioactive structural moieties, and by increasing structural flexibility.

**Figure 2:**
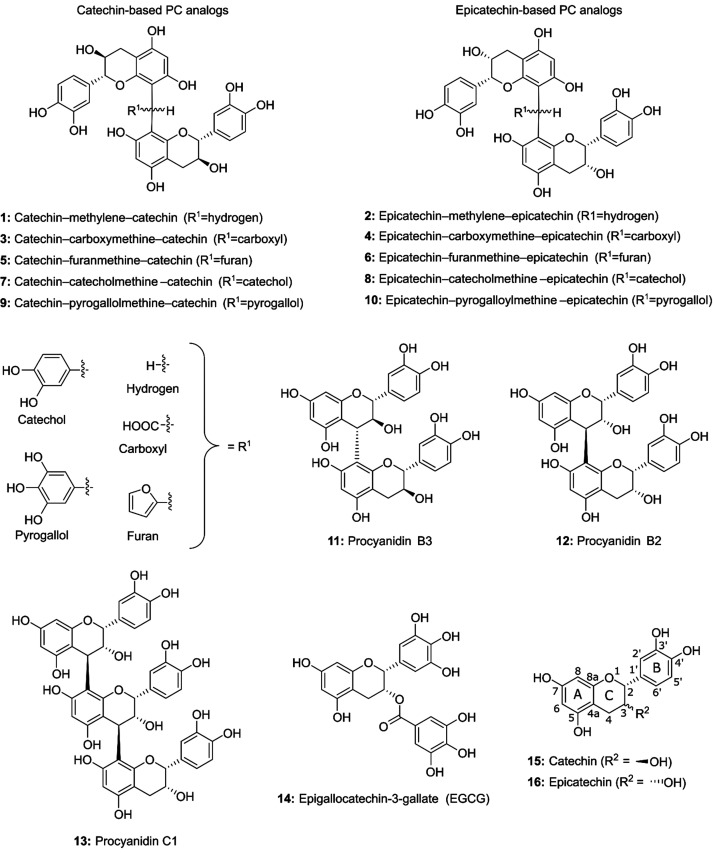
Structures of the hemisynthesized procyanidin analogs (**1**–**10**), natural procyanidin oligomers (**11**–**13**), epigallcocatechin-3-*O*-gallate (**14**), catechin (**15**), and epicatechin (**16**). The numbering of the atoms and lettering of the phenolic and heterocyclic rings of flavan-3-ols is presented in structures **15** and **16**.

Characterizing the products from reactions between PAs and aldehydes accurately poses a significant analytical challenge because the compositions of the starting materials, i.e., PA mixtures, are often a challenge to characterize accurately. Therefore, the purpose of this study was to investigate the properties of the simplest possible products of (epi)catechins and aldehydes, i.e., dimeric products to enable direct comparison of structurally analogous compounds. These synthetic dimers will be referred to as procyanidin (PC) analogs because they closely resemble procyanidins. Ten dimeric PC analogs were synthesized from catechin and epicatechin using several different aldehydes, i.e., formaldehyde, glyoxylic acid, furfural, 3,4-dihydroxybenzaldehyde (3,4-DBA), and 3,4,5-trihydroxybenzaldehyde (3,4,5-TBA; Figure 2). Then, the PC analogs were tested for their protein precipitation capacity (PPC), octanol–water partition, and stability in phosphate-buffered saline (PBS) solution. Within these tests, the PC analogs were compared to natural dimeric and trimeric PCs (B2, B3, and C1), to their starting materials (catechin and epicatechin), and to epigallocatechin-3-gallate (EGCG), which were obtained as commercial standards (Figure 2).

## 2 Material and Methods

### 2.1 Chemicals

EGCG, epicatechin, bovine serum albumin (BSA), l-ascorbic acid, 3,4-DBA, and phosphate buffered saline tablets were purchased from Sigma-Aldrich (MO, USA). One PBS tablet was dissolved in 200 mL of ultrapure water to produce 0.01 M phosphate buffer with pH of 7.4 and potassium and sodium chloride concentrations of 0.0027 and 0.137 M, respectively. The PC oligomers B3, B2, and C1 were purchased from Extrasynthese (Lyon, France). Catechin, 3,4,5-TBA, and glyoxylic acid were purchased from Carbosynth (Berkshire, UK). HPLC grade methanol, reagent grade formic acid, and LC–MS grade formic acid were purchased from VWR International (Pennsylvania, USA). Copper(II) sulfate was purchased from Riedel-de Haën, aqueous formaldehyde was purchased from J. T. Baker, and furfural was purchased from BDH. HPLC, and LC–MS grade acetonitrile and *n*-octanol were purchased from Honeywell (North Carolina, USA). Acetone-*d*_6_ was purchased from Eurisotop (Saint-Aubin, France). Ultrapure type I water was produced using a Merck Millipore Synergy UV water purification system (Darmstadt, Germany).

### 2.2 Synthesis of Procyanidin Analogs

The PC analogs were synthesized under acidic catalysis in 10 mL boiling flasks by utilizing the commonly used concept in synthesis of aldehyde-mediated catechins and other flavonoids in wine-like model solutions.^6−11^ However, the reactions conditions, i.e., solvent, pH, temperature, and concentrations, were optimized for this study. The reactions were carried out in 15% aqueous methanol to improve solubility, and the pH of the solvent was adjusted to 2.5 by using formic acid. The only exception was the reaction with glyoxylic acid, where the glyoxylic acid acted as the catalytic acid as well, and the solvent was, therefore, not acidified with formic acid. The reaction vessels were placed in a water bath at a temperature of 45 °C, and the water bath was constantly mixed to ensure an even temperature. The concentrations of catechin and epicatechin were 45 mM, and the concentration of the aldehydes were 90 mM for formaldehyde and furfural, 45 mM for 3,4-DBA and 3,4,5-TBA, and 22.5 mM for glyoxylic acid. The reactions with glyoxylic acid were catalyzed by Cu^2+^, which was added as copper(II) sulfate (1.5 mM). The copper catalysis in reactions between catechin and glyoxylic acid have been reported previously by Clark et al. (2003).^26^ The reactions were followed by UPLC–DAD at 280 nm, and the reactions were quenched by placing the boiling flasks in an ice bath after the suitable concentration of the desired products were formed. Typical reaction time for the reactions with formaldehyde, glyoxylic acid, and furfural was 1–2 h, while the reactions with 3,4-DBA and 3,4,5-TBA were carried out overnight (approximately 17 h). The products were purified using semipreparative reversed-phase HPLC-DAD (section 2.3) directly from the reaction mixtures with two subsequent injections of 5 mL. The products were characterized with high-resolution mass spectrometry, UV–vis spectroscopy, and NMR spectroscopy (sections 2.4 and 2.5).

### 2.3 Semipreparative HPLC–DAD

The synthesized compounds were purified using a semipreparative HPLC–DAD system from Waters that consisted of a Waters 2535 quaternary gradient module, a Waters 2998 photodiode array detector, and a Waters Fraction Collector III (Waters Corporation, Milford, MA). The utilized column was a reversed-phase C18 Gemini column from Phenomenex (particle size 10 μm, pore size 110 Å, 150 mm × 21.2 mm, i.d. 4.6 mm; Phenomenex, Torrance, CA). The eluents were (A) acetonitrile and (B) ultrapure water except with the PC analogs containing carboxymethine linkers (**3** and **4**). In the purification of these compounds, the B eluent was acidified with 0.1% formic acid. The gradients were optimized separately for each compound based on their retention times on the UPLC systems. The injection volume was 5 mL and the samples were filtered through 0.2 μm PTFE filters before injections.

### 2.4 UPLC-DAD and UPLC-DAD-HESI-Orbitrap-MS

Two separate Acquity UPLC systems were utilized in this study. Both systems consisted of a binary solvent manager, sample manager, and column oven (Waters Corporation, Wexford, Ireland). Two different columns were used in these systems. Both were Acquity BEH phenyl columns (1.7 μm particle size), one with dimensions of 100 mm × 2.1 mm i.d., and another with 30 mm × 2.1 mm i.d. (Waters Corporation, Wexford, Ireland). The eluents were (A) acetonitrile and (B) 0.1% aqueous formic acid. With the longer column, the flow rate was 0.5 mL/min and the gradient was the following: 0–0.5 min, 0.1% A and 99.9% B (isocratic); 0.5–5 min, A from 0.1% to 30% and B from 99.9% to 70% (linear), 5–8.5 min column wash (90% A and 10% B), and stabilization (0.1% A and 99.9% B). With the shorter column, the flow rate was 0.65 mL/min and the gradient was the following: 0–0.1 min, 3% A and 97% B (isocratic); 0.1–4 min, A from 3% to 50% and B from 97% to 50% (linear); 4–5.5 min, column wash (90% A and 10% B) and stabilization (3% A and 97% B).

One of the UPLC system was attached to a diode array detector (UPLC-DAD), and the other was attached to a diode array detector and a Q Exactive Orbitrap mass spectrometer (Thermo Fisher Scientific GmbH, Bremen, Germany; UPLC-DAD-HESI-Orbitrap-MS). A heated electrospray ion source (HESI) was used with the mass spectrometer. Nitrogen was used as a sheath and auxiliary gas, and their flow rates were 60 and 20 units, respectively. The spray voltage was set at −3000 V, S-lens RF level at 60, capillary temperature at 380 °C, and probe heater temperature at 300 °C. The full scan *m*/*z* range was 150–1700, with the automatic gain control being 3 × 10^6^ and the resolution 70 000. Nitrogen was used as the collision gas. Calibration of the MS system was done using Pierce ESI Negative Ion Calibration Solution (Thermo Fischer Scientific Inc., Waltham, MA). Data were processed with Thermo Xcalibur Qual Browser and Quan Browser.

### 2.5 NMR Spectroscopic Measurements

Bruker Avance-III 500 spectrometer (Billerica, MA) and a BB/1H Smartprobe (Fällanden, Switzerland) were utilized to perform the NMR experiments. The PC analogs were characterized by ^1^H (zg30) and ^13^C (zgpg30) 1D experiments, and COSY (cosygpmfphpp), HSQC (hsqcedetgpsisp2.3), and HMBC (hmbcetgpl3dn) 2D experiments. The *J*_CH_ coupling constants for the HSQC and HMBC experiments were set to 145 and 8 Hz, respectively. The samples were dissolved in acetone-*d*_6_ at approximately 5 mM concentration. The measurements were done in 5 °C to prevent degradation of the compounds during the experiments. The spectra were calibrated using the chemical shifts of the solvent (2.05 ppm for ^1^H and 29.84 ppm for ^13^C).^20^

### 2.6 Protein Precipitation Capacity

PPC was measured by using the turbidimetric well-plate reader assay of Engström et al. (2019), with few modifications to the volumes of the reactants and to the number of replicates, for instance. The analyzes of the supernatants were done following Engström et al. (2022).^21^ Briefly, 75 μL of BSA solution and 75 μL of the solutions of compounds **2**–**14** were pipetted to well plates, and the intensity of the forming haze was measured periodically at 420 nm using a well-plate reader. The PPC of the PC analogue **1** was not measured because it was not soluble in suitable solvents. The reactions were monitored for 30 min, and the highest intensities were recorded. A single background measurement was done at each concentration with all the components in the wells but the BSA. The reactions were done in triplicates (and in duplicates with PC C1), and the concentrations of the compounds **2**–**14** in the reaction mixtures were 0.25 0.65, 1.05, 1.45, 1.85, and 2.25 mM. The concentration of the BSA was kept constant at 0.1 mM. Therefore, the tested molar ratios to BSA were 2.5:1, 6.5:1, 10.5:1, 14.5:1, 18.5:1, and 22.5:1. After the 30 min, the supernatants and precipitates of the replicates were combined in a 1.5 mL Eppendorf tube and separated by centrifuging. The supernatants were then analyzed by UPLC–DAD using the shorter phenyl column (section 2.4) to quantify the leftover compounds **2**–**14** and BSA. Leftover BSA was quantified using a separately prepared linear calibration curve (1–125 μM), and the compounds **2–14** were quantified by analyzing the background solutions from the well-plate reader assay and constructing a calibration curve from these analyzes. BSA was quantified at 280 nm and compounds **2**–**14** at 280–320 nm. The wavelength of maximal UV absorption for compounds **2**–**14** could not be utilized in quantification because the diode array detector was saturated at the optimal wavelengths due to the high concentrations used in the well-plate reader assay. Therefore, off-optimal wavelengths were chosen for quantification separately for each compound **2**–**14** to reduce intensities and to obtain linear fits for calibration curves. From these results, the proportions of the precipitated compounds were estimated by subtracting the quantified concentrations in the supernatants from the known initial concentrations in the wells of the well plates.

### 2.7 Octanol–Water Partition

The octanol–water partition was done according to Virtanen et al. (2021).^22^ The shake-flask method was done using 2 mL Eppendorf tubes and three replicates. The concentration of each compound was 200 μM, and the compounds were first dissolved in water, which had been saturated with *n*-octanol. After mixing, aliquots of 500 μL were transferred to new Eppendorf tubes, and 500 μL of *n*-octanol was added that had been saturated with water. The solvent systems were mixed in a vortex mixer for 2 h, and then both the water and the *n*-octanol layers as well as the initial water solution were analyzed by UPLC–DAD using the shorter phenyl column (section 2.4). The chromatograms were integrated using TargetLynx software (version 4.2), and the octanol–water partition coefficient, log *P*, was calculated as the log_10_ of the ratio of area in *n*-octanol phase to the area in water phase. Recovery % was calculated by summing the areas in both phases and dividing the summation with the area measured from the initial water phase.

### 2.8 Stability in Phosphate Buffered Saline

The stabilities of the compounds were measured in PBS, which was prepared from commercially available tablets that were dissolved in ultrapure water. The phosphate concentration was 0.01 M, the pH was 7.4, and and the potassium and sodium chloride concentrations were 0.0027 and 0.137 M, respectively. The concentrations of the compounds **1**–**16** were 150 μM. The compounds were dissolved in 2 mL of the PBS solution in Eppendorf tubes, vortexed for approximately 15 min, and filtered through a 0.2 μm PTFE filter to a 2 mL UPLC vial. The solutions were analyzed with the UPLC-DAD-HESI-Orbitrap-MS instrument using the longer phenyl column (section 2.4), and repeated injections were done every 47.5 min for approximately 12 h. With compounds **15** and **16**, an additional final time point was measured at 26 h. The UV chromatograms at 280 nm were integrated using the QuanBrowser module in Xcalibur software (version 4.1.31.9), and the main degradation products were identified in QualBrowser by their high-resolution mass spectra and UV spectra. With each compound, the measured areas were transformed to proportional areas by dividing all areas with the initial area in the first injection of the time series, and the data was further log_e_ transformed and fitted with first-order linear regression curves. The regression curves were supplemented with 95% confidence intervals to visualize the uncertainty in the fitted curves. The half-lives were calculated from the slope (*k*) parameters of the linear regression model using the following equation:
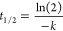


### 2.9 Statistical Analysis

All statistical analyzed were performed with R (version 4.2.2) in the integrated development environment Rstudio (version 2022.12.0.353).^23,24^ The PPC data was fitted with a dose–response model using the package *drc*.^25^ Various models, e.g., logit and log-normal models, were compared using the mselect function, and the best fitting model was chosen based on the Akaike’s information criterion and residual error when comparing all models, and log-likelihood was used when comparing nested models. All compounds were assumed to have the same upper and lower asymptotes; the former was estimated from the experimental data, and the latter was fixed at zero. Compounds that displayed no activity in the PPC experiment (**15** and **16**) or were otherwise practically inactive (**11** and **12**) were excluded from the model.

## 3 Results and Discussion

### 3.1 Synthesis and Characterization of the Procyanidin Analogs

The utilized aldehydes were chosen for this study based on a few different reasons (Figure 2). Formaldehyde was chosen to introduce the simplest possible linking unit to the PC analogs, i.e., an unsubstituted methylene group (**1** and **2**). Compared with PC dimers B2 and B3, the molecular weights of the methylene-linked dimers were approximately the same. Moreover, the methylene group itself was not expected to participate in the interaction with the model protein in the PPC experiment due to it being a small and nonpolar moiety. In other words, any differences in the properties of the PC oligomers and methylene-linked PC analogs would be explained by the increased structural flexibility. Glyoxylic acid was chosen because it is known to react exceptionally well with anthocyanins and flavan-3-ols.^26,27^ Furfural has also been demonstrated to react with catechins forming furanmethine-linked catechin dimers (**5** and **6**).^28^ The 3,4-DBA and 3,4,5-TBA have never been utilized in hemisynthesis of PC analogs or anthocyanin–catechin adducts before, most likely because these aldehydes are not found in red wines, but reactions with unsubstituted benzaldehyde have been performed.^8,29^ However, the reactions with 3,4-DBA and 3,4,5-TBA incorporate a catechol and a pyrogallol group, respectively, to the PC analogs. With catechins and gallocatechins, it has been shown that these groups, i.e., the B-rings are the sites that interact with BSA more strongly than other parts of the molecules, and the B-rings participate in protein interactions between PC dimers and proline-rich protein IB7_14_.^19,30^ Therefore, it was hypothesized that these linking units in compounds **7**–**10** could actively participate to the protein interaction and, thereby, increase the PPC in comparison to PC analogs **1** and **2**, for instance.

The synthesis protocol was designed so that the necessary amount of products (approximately 10 mg or more) could be synthesized and purified in a single working day in most reactions. The reactions with formaldehyde, glyoxylic acid, and furfural lasted typically 1–2 h before a suitable amount of products could be isolated. The reactions with 3,4-DBA and 3,4,5-TBA were run overnight because these reactions did not proceed to form polymeric species and precipitate, which happened with the other three aldehydes and which limited their reaction times to the above-mentioned 1–2 h. Due to the longer reaction times, a lower concentration of 3,4-DBA and 3,4,5-TBA could be utilized compared to reactions with furfural and formaldehyde. The lower concentrations of 3,4-DBA and 3,4,5-TBA were also desired to ease the semipreparative purification. The concentration of glyoxylic acid was 22.5 mM because at this concentration the pH of the solution was approximately 2.5, i.e., the same as that with the other reactions. The yields of the isolated products were 3–18%. The lowest isolated yields were obtained for methylene-linked PC analogs **1** and **2**. The yields of the other compounds were 8–18% with the furanmethine and carboxymethine-linked PC analogs (**3**–**6**), providing the best yields. Individual yields are presented in the Supporting Information (SI) along with the compound characterization data. The reported yields were only for the individual main products of the reactions, and they do not represent the overall yield for all the positional isomers of the dimeric products and the higher oligomers, which all reactions produced. As mentioned, the reactions with some aldehydes (formaldehyde, furfural, and glyoxylic acid) proceeded to form oligomeric mixtures that precipitated out of the solutions if the reactions were left unattended. In these cases, nearly all (epi)catechin was consumed in the reactions (data not shown). Elsewhere, acetaldehyde and catechin have been shown to similarly produce precipitates consisting of oligomeric products with yields up to 90%.^31^ That is to say, the yields can be excellent in applications in which precipitates of oligomeric and polymeric mixtures are the desired products. Here, however, only the main dimer from each reaction was targeted and isolated, which explains the lower isolated yields.

The PC analogs were characterized based on their high-resolution mass spectra, UV-spectra, and NMR spectra. The molecular formulas calculated from the mass spectra corresponded to the expected products, and the errors between calculated exact masses and measured masses were 2 ppm or less in every measurement throughout the study (SI). The UV spectra were nearly identical to the starting materials, i.e., to catechin and epicatechin, and no new absorption maxima were observed above 280 nm for the PC analogs. This was expected because the PC analogs did not contain further conjugated systems compared to the starting materials. Based on the MS and UV data, the synthesized PC analogs were interpreted to be one of the possible positional isomers where the linkage could vary between C6 and C8 positions. Similar aldehyde-mediated dimers of flavan-3-ols and flavan-3-ols and anthocyanins have been hemisynthesized and isolated previously.^7,32−36^ The reported main dimeric products have always been C8–C8 linked, which is why the PC analogs hemisynthesized in this study were expected to be C8–C8 linked as well. NMR spectroscopy was used to verify this assumption. Compounds **1**–**6** and **8** and **10** were verified to be C8–C8 linked based on HMBC correlations. Briefly, the singlet ^1^H signals of the hydrogens in the linking methylene or methine groups were observed to correlate with the C8a carbons of the (epi)catechin units, which was expected only if the methylene or methine groups were attached to the C8 positions. The position of the linkage in compounds **7** and **9** could not be conclusively established in the same way because the hydrogen in the methine group did not show these same correlations in the HMBC spectra. The HMBC spectra of **7** and **9** showed fewer correlations in general compared to other PC analogs, and the linkage position could not be verified with other correlations either. The lack of HMBC correlations could be related to the broadened peaks in ^1^H NMR spectra of compounds **7**–**10**, which is discussed further in the SI. Nonetheless, the ^1^H and ^13^C NMR spectra of **7** and **9** were nearly identical to the corresponding spectra of **8** and **10**, all other PC analogs besides **7** and **9** were confirmed to be C8–C8 linked, and the literature data of similar reactions support the results of this study regarding the linkage position of the main products.^7,32−36^ Therefore, compounds **7** and **9** were assumed to be C8–C8 linked as well. The NMR spectroscopic data are presented as SI, Figures S1–S50, together with more discussion about the characterization and assignation of the spectra.

### 3.2 Protein Precipitation Capacity

**Figure 3:**
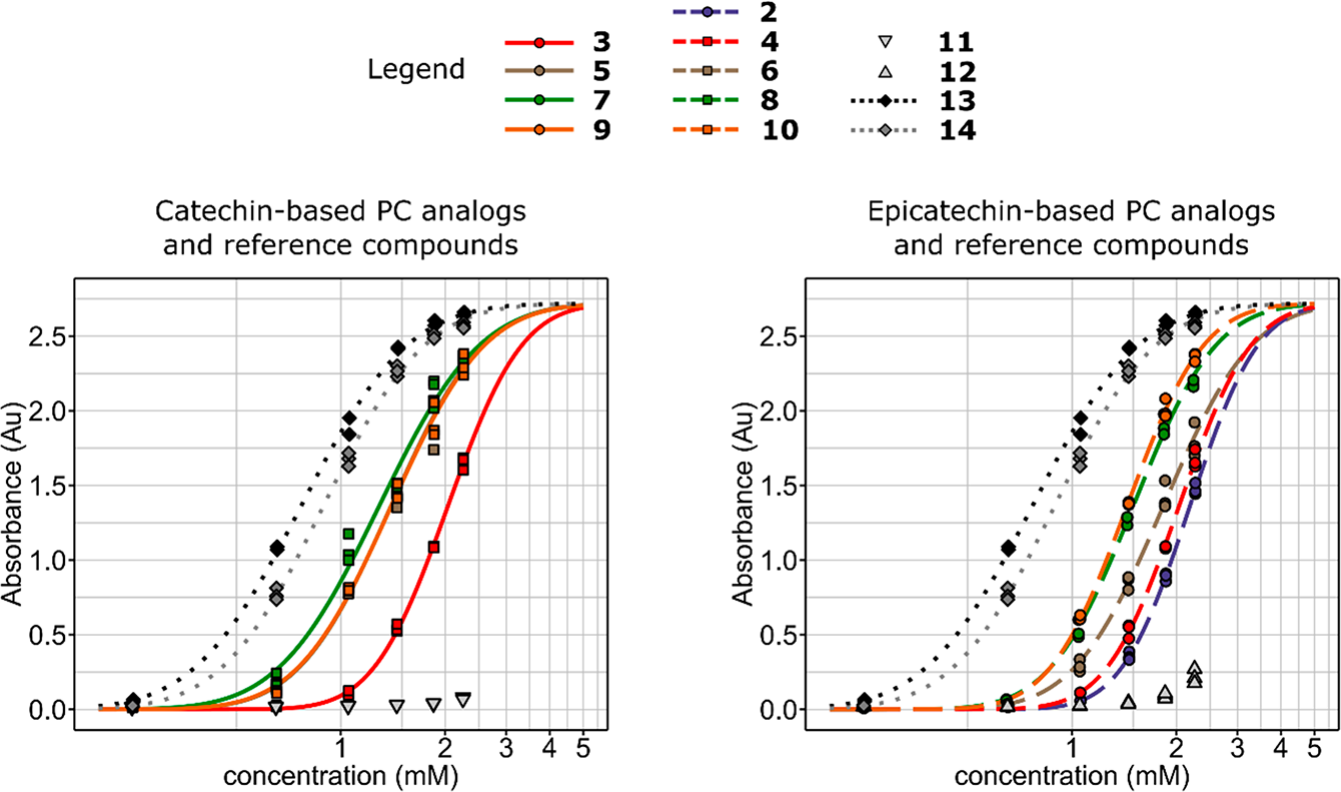
Plots of the log-normal dose–response model for the protein precipitation capacity. The concentration in the *x*-axis is the concentration of each compound **2**–**14** in the reaction mixture, while the concentration of BSA was kept at a constant of 0.1 mM. The compounds that had no activity (**15**, **16**), low activity (**11**, **12**), or were not soluble to suitable solvents (**1**) were excluded from the model. The common lower asymptote was assumed to be zero, and each compound was assumed to have the same upper asymptote, which was estimated from the data. Refer to Table 1 for the estimates of the parameters (effective doses and slope).

The data from the PPC experiment were analyzed by fitting log-normal dose–response models to the data (Figure 3, Table 1, and SI, Figure S51). The PC dimers B2 and B3 (**11** and **12**) were excluded from the model because of their low PPC, and epicatechin and catechin (**15** and **16**) were excluded because they had no PPC at all. The statistical model was restricted by fixing the lower asymptote to zero because the absorbances at lower concentrations were practically zero after subtraction of background. The upper asymptote was assumed to be the same for all compounds because all tested compounds had relatively similar structures and molecular weights, and thus, the mechanisms of interactions were expected to be similar. Moreover, the experimental data supported this assumption because the responses of the most active compounds leveled at around the same absorbance values (Figure 3), and the BSA was shown to have been precipitated almost completely at the highest absorbances with the most active compounds (SI, Figure S52), which will be discussed later in detail. The following observations were made based on visual observations of the fitted dose–response models (Figure 3, and SI, Figure S51) and on the parameters of the models (Table 1). There was less variation in the slope parameters and more variation in the effective dose parameters (ED_50_ and ED_90_), which were, therefore, primarily used in assessing the performance of the compounds.

**Table 1:** Parameters of the Log-Normal Dose–Response Model for the Protein Precipitation Capacity^a^

compd	ED_50_ (mM)	ED_50_ (molar ratio to BSA)	ED_90_ (mM)	ED_90_ (molar ratio to BSA)	slope
**1**^b^					
**2**	2.18 (0.02)	22:1	3.45 (0.10)	35:1	2.80 (0.13)
**3**	2.03 (0.02)	20:1	3.34 (0.09)	33:1	2.57 (0.10)
**4**	2.03 (0.02)	20:1	3.30 (0.08)	33:1	2.64 (0.11)
**5**	1.39 (0.02)	14:1	2.58 (0.06)	26:1	2.08 (0.07)
**6**	1.83 (0.02)	18:1	3.32 (0.09)	33:1	2.15 (0.08)
**7**	1.29 (0.02)	13:1	2.53 (0.07)	25:1	1.90 (0.06)
**8**	1.50 (0.02)	15:1	2.61 (0.06)	26:1	2.30 (0.08)
**9**	1.40 (0.02)	14:1	2.61 (0.06)	26:1	2.04 (0.06)
**10**	1.44 (0.01)	14:1	2.41 (0.05)	24:1	2.48 (0.08)
**11**^c^					
**12**^c^					
**13**	0.77 (0.01)	8:1	1.57 (0.06)	16:1	1.79 (0.08)
**14**	0.89 (0.01)	9:1	1.76 (0.06)	18:1	1.88 (0.07)
**15**^d^					
**16**^d^					

a The compounds that had no activity (**15**, **16**), low activity (**11**, **12**), or were not soluble to suitable solvents (**1**) were excluded from the model. Common lower asymptote was assumed to be zero, and each compound was assumed to have the same upper asymptote, which was estimated from the data to be 2.72 (standard error 0.03). The values in the parentheses are standard errors (SE) for the estimates. The molar ratios at effective dose 50 (ED_50_) and effective dose 90 (ED_90_) concentrations were calculated manually, i.e., the molar ratio was not a parameter of the dose–response model.

b Was not soluble in sufficient concentrations to suitable solvents.

c Had only low activity that was considerably lower than the other compounds.

d Did not have any activity.

All tested PC analogs (**2**–**10**) had a significantly improved PPC compared to natural PC dimers B2 and B3 (**11** and **12**; Figure 3). With the epicatechin based dimers, the highest activity was achieved by the dimers with catecholmethine and pyrogallolmethine linkers (**8** and **10**), followed by the dimer with a furanmethine linker (**6**). The activity of the dimers with carboxymethine (**4**) and methylene (**2**) linkers were approximately similar, albeit the PPC of **4** was marginally better. There were small differences to this trend with the catechin based dimers, as the dimer with furanmethine linker (**5**) had similar activity to the dimers with catecholmethine and pyrogallolmethine linkers (**7** and **9**). Overall, the catechin based dimers had slightly better PPC than the epicatechin based dimers (dimers with catecholmethine and furanmethine linkers) or the PPCs were equal (dimers with pyrogallolmethine and carboxymethine linkers; SI, Figure S51). The ED_50_ concentration of the best performing synthetic PC analogs was approximately 36% lower than the ED_50_ concentration of the worst performing PC analogs. Importantly, both PC dimers B2 and B3 were practically inactive in the utilized experimental setup and only the highest concentrations showed any haze formation at all (Figure 3). The PPC of trimer C1 (**13**) was greatly improved compared to the dimers B2 and B3, and it exceeded the activities of the synthetic PC analogs as well. EGCG (**14**) was also more active than the PC analogs, but it was less active than trimer C1. On average, however, both PC C1 and EGCG achieved nearly 90% of their maximal absorbances at concentration where the synthetic PC analogs achieved only 50% (Table 1, Figure 3).

The 3,4-DBA and 3,4,5-TBA were chosen for this study as starting materials because it was hypothesized that the PPC could be improved by introducing moieties to the PC analogs that could participate in the tannin–protein interactions. Noncovalent tannin–protein interaction is a complex phenomenon, and several mechanisms have been suggested to cause the interactions and further precipitation. The most prominent mechanisms are hydrophobic interactions, π–π stacking, and hydrogen bonding.^18,37^ Moreover, the precipitation is a 2-fold process where protein is first covered by tannins, and the protein–tannin complexes then precipitate by cross-linking as the aggregated complexes become less soluble.^18^ Our hypothesis was that the additional catechol and pyrogallol units in **7**–**10** could participate directly in either the formation of BSA–PC analogue complexes or their further cross-linking by hydrogen bonding, for instance. Indeed, the dimers catecholmethine and pyrogallolmethine linkers (**7**–**10**) turned out to be more active than the dimers with methylene or carboxymethine linkers (**2**–**4**), which could be explained by the participation of the linker unit in the protein interactions. However, there was not a significant difference in PPC between the PC analogs with catecholmethine and pyrogallolmethine groups (**7**–**10**) in the linking units, and the dimer with catecholmethine linker (**8**) even outperformed the pyrogallolmethine-linked dimer (**10**) with epicatechin-based PC analogs. Clearly, the additional hydroxyl group in the pyrogallol moieties did not further improve the PPC. The number of possible sites for hydrogen bonding is one feature affecting the protein affinity and aggregation of PC analogue–protein complexes, but other properties play a role as well. For instance, three-dimensional structure of PAs affect their interactions with proteins,^19^ and structural flexibility of tannins in general is a recognized factor that contribute to their ability to interact with proteins.^18^ The third hydroxyl group in the pyrogallol unit in **9** and **10** could produce conformational constrains compared to the catechol units in **7** and **8** so that the additional hydroxyl group could not participate to the protein interactions by hydrogen bonding or that the pyrogallol moieties could not interact with proteins via hydrophobic interactions any better than the catechol moieties.

The PC analogs with furanmethine linkers, i.e., **5** and **6** had better PPC than dimers with only methylene and carboxymethine linkers (**2**–**4**), suggesting that the furanmethine unit had an active role in the protein precipitation. The oxygen atom in the furan moiety cannot act as a donor in hydrogen bonding, but it can act as a hydrogen bond acceptor and, thereby, interact with BSA via hydrogen bonding. Naturally, the furanmethine moiety might participate in protein precipitation by other mechanisms too, such as hydrophobic interactions. Importantly, the added structural flexibility of the PC analogs alone was a highly important factor in improving the PPC. The PC dimers B2 and B3 (**11** and **12**) had significantly weaker PPC than the PC analogs **2**–**10**, even when compared to PC analogs where the linking unit could only add structural flexibility and not participate to the protein interactions directly, i.e., the dimers with carboxymethine and methylene linkers (**2**–**4**).

The supernatants from the PPC experiments were analyzed by UPLC–DAD to determine the relative proportions of the precipitated reactants (SI, Figure S52). This data supported the primary PPC data from the well-plate reader assay and the assumption that the same upper asymptote could be assumed for all compounds in the dose–response model. This could be concluded because the precipitation patterns were similar between compounds, i.e., at the same absorbances in the well-plate reader assay, approximately the same proportion of BSA was precipitated out of the solution (SI, Figure S52). At the ED_50_ concentration of each compound (Table 1), for instance, approximately 20–25% of BSA was precipitated (SI, Figure S52). Based on the proportions of precipitated BSA, the PC dimers B2 and B3 (**11** and **12**) were practically inactive, while the trimer C1 and EGCG (**13** and **14**) precipitated almost all BSA at the highest tested concentration. The least active PC analogs (**2**–**4**) precipitated approximately 25% of the BSA out of the solution at the highest tested concentration of 2.25 mM (molar ratio to BSA 23:1), whereas the most active PC analogs precipitated approximately 55%.

### 3.3 Octanol–Water Partition

The octanol–water partition coefficients (*K*_ow_ and log *P*) were determined for the compounds to measure how the various linking units affect the hydrophobicity of the synthetic PC analogs (Table 2). The measured log *P* values of the dimers B2 and B3 and trimer C1 were −0.87, −0.79, and −1.17, respectively, meaning the PC oligomers were hydrophilic. The log *P* of the synthetic PC analogs varied from hydrophilic (negative log *P*) to hydrophobic (positive log *P*) depending on the linking units (Table 2). The dimers with furanmethine (**5** and **6**) and methylene linkers (**1** and **2**) were hydrophobic, whereas the dimers with carboxymethine, pyrogallolmethine, and catecholmethine linkers were hydrophilic (**3**, **4**, **7**–**10**). With each different linking unit, the synthetic dimers made of epicatechin were more hydrophilic than their counterparts made of catechin. The same was not observed for dimers B2 and B3, as the log *P* values were practically identical.

**Table 2:** Octanol–water Partition Coefficients of Compounds **1**–**16**^b^

compd	*K*_OW_	log *P* (log_10_*K*_OW_)	recovery (%)
**1**	5.00 (0.05)	0.70 (0.00)	94.7 (0.6)
**2**	1.46 (0.01)	0.16 (0.00)	95.9 (0.7)
**3**	0.04 (0.00)	–1.37 (0.01)	97.6 (0.2)
**4**	0.02 (0.00)	–1.78 (0.01)	97.7 (0.0)
**5**	5.28 (0.02)	0.72 (0.00)	90.9 (0.8)
**6**	2.08 (0.00)	0.32 (0.00)	94.6 (0.3)
**7**	0.79 (0.00)	–0.10 (0.00)	96.9 (0.4)
**8**	0.34 (0.00)	–0.47 (0.00)	96.4 (0.4)
**9**^a^	0.14 (0.01)	–0.85 (0.03)	99.4 (1.2)
**10**^a^	0.07 (0.00)	–1.18 (0.01)	107.3 (0.7)
**11**	0.16 (0.00)	–0.79 (0.00)	97.4 (0.1)
**12**	0.13 (0.00)	–0.87 (0.00)	94.3 (0.6)
**13**	0.07 (0.00)	–1.17 (0.00)	97.6 (0.0)
**14**	13.58 (0.06)	1.13 (0.00)	94.3 (0.6)
**15**	2.61 (0.03)	0.42 (0.00)	91.3 (0.5)
**16**	1.44 (0.01)	0.16 (0.00)	91.1 (0.3)

a Tentative values, the experiment was shorter than with other compounds because the compounds degraded during the full-length two-hour experiment.

b The values in the parentheses are standard errors for the average parameters.

The simplest linking unit that only added structural flexibility, i.e., the methylene-linkage, caused the dimers **1** and **2** to become hydrophobic in comparison to natural PC dimers B2 and B3 (**11** and **12**; Table 2). The mechanistic reasons for this effect cannot be established without further and more detailed experiments, and possible utilization of computational chemistry. However, the additional carboxyl group in **3** and **4** in comparison to **1** and **2** made the PC analogs hydrophilic, which was likely caused by the addition of the polar carboxyl moiety that enhanced solvation with water. Similarly, the additional hydroxyl group in **9** and **10** likely improved the solubility of these compounds in water in comparison to those of **7** and **8** due to the additional hydroxyl moiety, enabling a more efficient solvation with water. Interestingly, EGCG was strongly lipophilic, despite it having two pyrogallol groups. However, this result was well in line with previous experiments in literature.^38^

The variation in the hydrophobicity was considerable, and this provides a useful way of controlling the solubility of the PC analogs. Moreover, it has been suggested that low *K*_ow_, i.e., negative log *P* would be favorable in the context of ruminant feeds to avoid any negative nutritional effects, because high *K*_ow_, i.e., positive log *P*, could be a sign of potential toxicity.^38^ Importantly, the PPC did not correlate here with the log *P* (*r* = −0.25, *p* = 0.45), which meant that the hydrophobicity could be adjusted by selecting a suitable linking unit without simultaneously reducing PPC. For instance, the dimers with furanmethine linkers (**5** and **6**) had good PPC and they were hydrophobic, while the catecholmethine-linked and pyrogallolmethine-linked dimers (**7**–**10**) had at least equal PPC but they were hydrophilic (**9** and **10**) or they were at the middle of the log *P* scale (**7** and **8**).

### 3.4 Stability in Phosphate Buffered Saline

**Figure 4:**
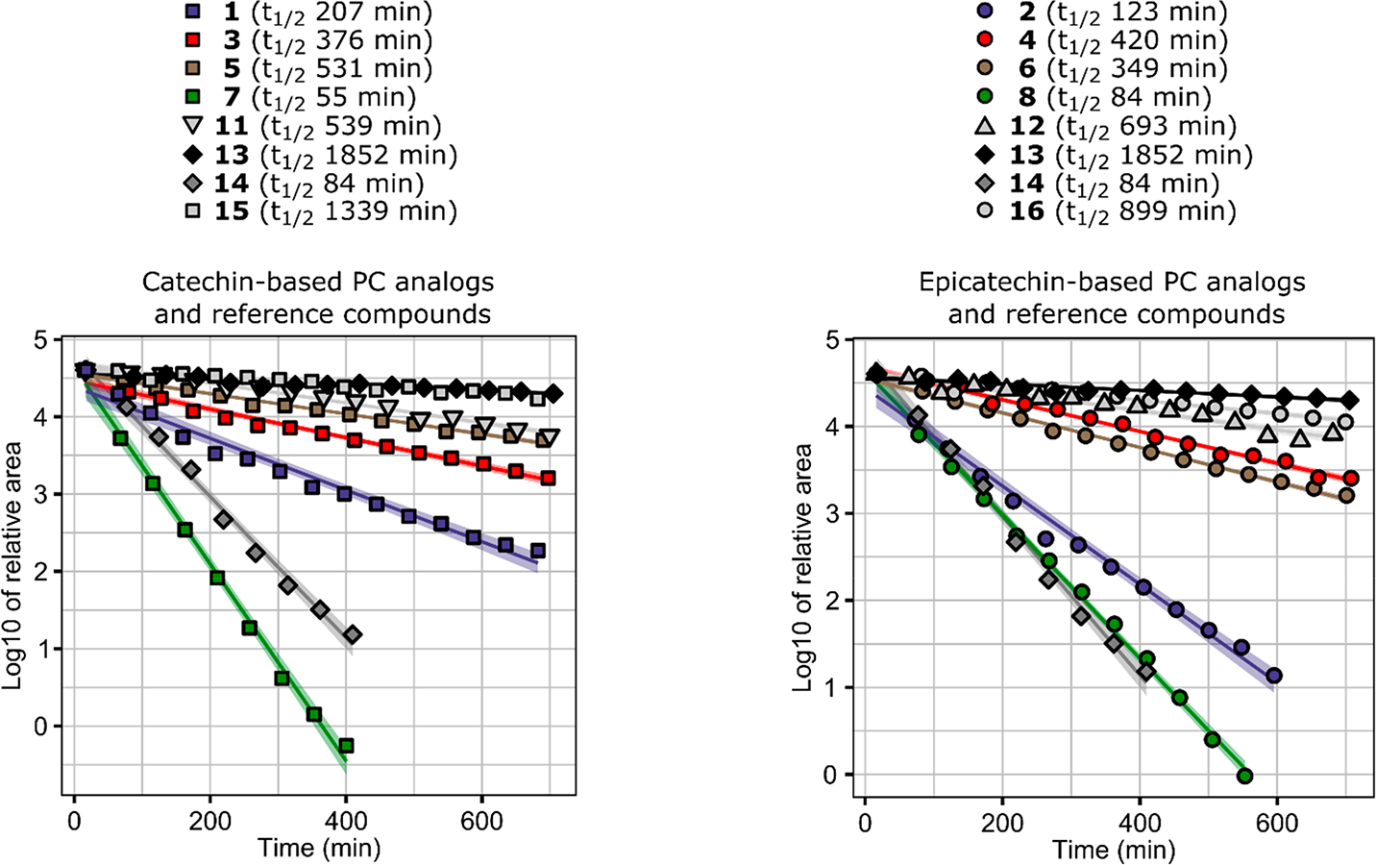
Stability of compounds **1**–**8** and **11**–**16** in phosphate buffered saline. Compounds **9** and **10** were completely degraded already at the first time point. UV chromatograms were integrated at 280 nm, and with each compound the areas were transformed to proportional areas by dividing the chromatogram areas with the initial area in the first time point. The degradation followed a first-order kinetics, so the relative areas were further log_e_ transformed to obtain linear fits for the regression models. Half-lives are presented in parentheses in the legend. The colored areas around the fitted curves are 95% confidence intervals for the linear fits.

**Figure 5:**
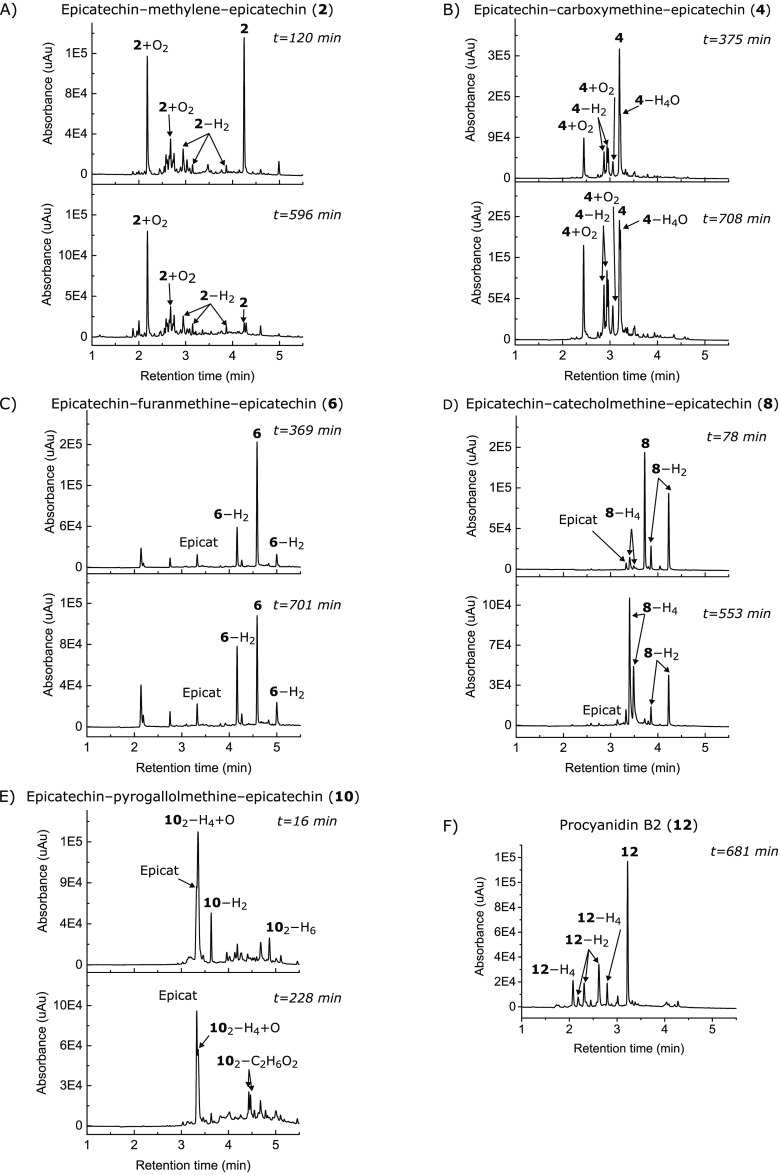
UV chromatograms (280 nm) of the epicatechin-based PC analogs and PC B2 from the stability experiments. Upper chromatograms are at the half-life of each compound and lower ones are at the last time point that was utilized for the kinetics plot (Figure 4), i.e., at a time point when all starting material had degraded or at the end of the experiments. The procyanidin B2 chromatogram is from the last time point from the end of the experiment.

The stabilities of compounds **1**–**16** were measured under physiologically relevant conditions using a commercial PBS solution. PBS is used as a buffer in various *in vitro* assays, which makes it important to understand the behavior of the PC analogs in such conditions, especially because flavonoids containing catechol or pyrogallol moieties are unstable in PBS solutions or in neutral phosphate buffers.^39−42^ Additionally, other polyphenols such as hydrolyzable tannins are unstable in PBS buffers as well.^41,42^ Half-lives (*t*_1/2_) were calculated from the kinetic data for each compound (Figure 4), and the initial degradation products were characterized using high-resolution mass spectrometry and UV data (Figure 5, and SI, Table S1, Figures S53–S60). Chromatograms of the epicatechin-based PC analogs from the stability experiments are presented in Figure 5 as examples, and the chromatograms of the other compounds are presented in SI, Figures S53 and S60 because both epicatechin and catechin based dimers behaved similarly in the experiments.

Generally, the natural PC oligomers (**11**–**13**) were more stable in the tested matrix compared to the synthetic PC analogs, and the PC trimer C1 was especially stable as it had the highest half-life (*t*_1/2_ = 1852 min) of all tested compounds (Figure 4). The dimers with carboxymethine (**2**, **3**) and furanmethine (**4**, **5**) linkers were the most stable synthetic PC analogs (*t*_1/2_ = 349–531 min) followed by the dimers with methylene-linkage (**1**, **2**; *t*_1/2_ = 131–207 min). The PC analogs with catecholmethine linkers (**7**, **8**; *t*_1/2_ = 55–84 min) and pyrogallolmethine linkers (**9**, **10**) were the least stable PC analogs, and the half-lives of **9** and **10** could not even be determined because no starting material was present even at the first time point. This meant that the half-lives of compounds **9** and **10** were considerably less than 15 min, which was the approximate time it took from adding the PBS buffer until the start of the first analysis. Interestingly, the half-life of EGCG (**14**) was similar to the half-lives of **7** and **8** despite it containing two pyrogallol groups.

During the PBS incubations, all compounds yielded products with molecular formulas M–H_2_, where the M stands for the molecular formula of a tested compound and the notation means the loss of two hydrogens. This notation, and analogous notation with compound numbers, will be used throughout to express the changes in the molecular formulas. M–H_2_ were the main degradation products with PC analogs containing catecholmethine or furanmethine linkers (**5**–**8**) and with all PC oligomers (**11**–**13**; Figures 5, and SI, Figures S55–S56 and S58, Table S1). Moreover, the resulting mixtures of products were less complicated with these compounds than with the other tested compounds, as only the products M–H_2_, and M–H_4_ with compounds **7** and **8**, were formed in significant quantities. Interestingly, however, the M–H_2_ products formed from different tested compounds were most likely not structurally similar, because their UV spectra had small differences. For instance, the **4**–H_2_ and **6**–H_2_ had similar UV spectra but the UV spectrum of **8**–H_2_ was evidently different, while the UV spectra of the initial compounds **4**, **6** and **8** were identical (SI, Figures S54–S56). The **10**–H_2_ and **12**–H_2_ had similarities in their UV spectra (SI, Figures S57 and S58), but they differed from the spectra of the previously mentioned three products **4**–H_2_, **6**–H_2_, and **8**–H_2_. Again, the UV spectra of the initial compounds **10** and **12** were identical, and they matched the spectra of **4**, **6**, and **8** as well. Similarities in UV spectra were observed between the **12**–H_4_ and **4**–H_2_ and **6**–H_2_, suggesting that the compounds had similarities in their structure even though the **12**–H_4_ product was further oxidized than **4**–H_2_ and **6**–H_2_ (SI, Figures S54, S55, S58).

The molecular formulas M–H_2_ could correspond to formation of either *o*-quinone structures from the catechol or pyrogallol groups (linker units or the B-rings) or to formation of new intramolecular linkages between the constituting units. From these two possibilities, the formation of *o*-quinone structures was less likely for two reasons. *O*-quinones are electrophilic and highly unstable in aqueous media and they react with nucleophiles, such as the phloroglucinol rings of (epi)catechins (A-ring) forming oligomers, or with dissolved O_2_, for instance.^43,44^ Second, flavonoids with *o*-quinone structures absorb visible light at around 400 nm and above,^43,45^ which none of the M–H_2_ products did. The M–H_2_ products exhibited only a new shoulder above the absorption maxima at 280 nm in their UV spectra compared to the UV spectra of starting materials. Therefore, the M–H_2_ molecular formulas corresponded more likely to formation of new intramolecular linkages between the building units. Even though the M–H_2_ products did not likely contain *o*-quinone structures, catechins are capable of forming dimers in few different ways via reactive *o*-quinones,^39,46,47^ and these reactions could play a role in the formation of the proposed new intramolecular linkages in M–H_2_ products. Catechins are known to dimerize by a mechanism, where a nucleophilic C8 or C6 carbon of the A-ring of intact catechin attacks the strongly electrophilic *o*-quinone moiety in the B-ring of another catechin unit, forming a C–C linkage between the A and B rings.^46,47^ Numerous such products, which are called dehydrocatechins, and further transformed products have been characterized as a result of enzymatic or auto-oxidation of catechins. On the other hand, a C–O–C ether linkage can be formed between a hydroxyl group of the A-ring of one catechin unit and the B-ring of another catechin unit via semiquinone radical intermediate.^46^ In the dimerization of EGCG, a new C–C linkage is formed between the B-rings by dismutation reaction between two *o*-quinone structures resulting in the formation of theasinensins, e.g., EGCG dimers.^48^ For catechins, however, this mechanism is only hypothetical, as the mechanism has so far only been proposed for EGCG, and the additional hydroxyl group in the B-ring of EGCG could be essential. All the above-described mechanisms resulting in the formation of (epi)catechin and EGCG dimers have been reported to occur with monomeric starting materials, but in this study, the formation of new linkages between the constituting units or the linker units would be intramolecular reactions that have not been reported before.

UV maxima at higher wavelengths beyond 270–280 nm were observed with further oxidized M–H_4_ products of the catecholmethine-linked dimers (**7**–H_4_ and **8**–H_4_) and with the M–H_4_O products of the carboxymethine-linked dimers (**3**–H_4_O and **4**–H_4_O; SI, Figures S54 and S56). The structures of **7**–H_4_ and **8**–H_4_ can be speculated to be either products with *o*-quinone structures, or they could correspond to the formation of yet another intramolecular linkage that would result in an extended conjugated system to explain the absorbance maxima at 430 nm and above (SI, Figure S56). The colored products **3**–H_4_O and **4**–H_4_O, however, can be identified to be xanthylium salts based on the literature data.^27,49^ Carboxymethine-linked dimers of catechin have been observed to further react to xanthylium salts, which have an additional pyran ring connecting the A-rings of the catechin units.^27^ Here, the characterization of the xanthylium salts was supported by the characteristic absorbances at 275 and 435 nm (SI, Figure S54).^27^ The xanthylium salts have been suggested to form through a condensation of the OH groups at C-7 positions (−H_2_O) to form a new heterocyclic ring between the building units and by a subsequent oxidation (−H_2_), which results to the overall loss of a H_4_O unit. Xanthylium-type structures have also been described to form from furanmethine-linked catechins in mildly acidic conditions,^28^ but such products were not observed in this study. In general, the carboxyl moiety in the linking unit in **3** and **4** should not be essential for the formation of the xanthylium-type structures, meaning that similar products could form from other PC analogs as well. However, the corresponding M–H_4_O products were not observed with the other compounds.

The least stable compounds, i.e., EGCG (**14**) and the PC analogs with pyrogallolmethine linkers (**9**, **10**), contained a common structural feature, which was the pyrogallol group. All these compounds were observed to oligomerize during the stability experiment, leading to dimeric products (Figure 5, and SI, Figures S57 and S59, Table S1). Flavonoids with pyrogallol groups, such as EGCG and myricetins, have been shown to be highly unstable at high pH and even in neutral conditions in phosphate buffers.^39,40,50^ The mechanism of dimerization of EGCG has been suggested to involve formation of *o*-quinone structures in the B-rings of two EGCG molecules, which then react together by dismutation to form C–C-linked EGCGs via an dehydrotheasinensin intermediate.^48^ With the PC analogs **9** and **10**, dimeric products (consisting of four catechin units in total) with a new single bond between the building units were not observed at all, but rather all dimers were further transformed species, such as **10**_2_–H_2_, **10**_2_–H_4_+O and **10**_2_–C_2_H_6_O_2_ (Figure 5, and SI, Figure S57, Table S1). Additionally, the dimers **9** and **10** were the only compounds that degraded back to catechin and epicatechin in significant proportions, although the formation of catechin and epicatechin was observed with other compounds as well but to a lesser extent. Some of the degradation products of **14** could be characterized based on literature data from previous experiments done in phosphate buffers.^48,50^ The molecular formulas of two degradation products of **14** corresponded to theasinensin (**14**_2_) and theasinensin P-2 (**14**_2_–CH_2_O; SI, Table S1). The former is a dimer of **14** with a single C–C bond between the B-rings, and the latter is a further oxidized product that has additional modifications in the B ring.

In addition to the unstable compounds **9**, **10**, and **14**, the more stable compounds **1**–**4** produced multiple major products with an increased number of oxygen atoms, most notably the M + O_2_ products of **1**–**4** (Figure 5, and SI, Figure S53 and S54). The formation of these products most likely the addition of either dissolved oxygen or water, but their precise structures could not be even tentatively identified here. However, similar changes in molecular formulas have been observed when theasinensins further oxidized in phosphate buffers.^50^ The oxidation reactions of theasinensins could, therefore, imply what kinds of structural changes happened to compounds **1**–**4** when they formed the M + O_2_ products, but these speculations will be omitted here.^50^ Nonetheless, it was an interesting pattern that the M + O_2_ products were only formed from the PC analogs with the less sterically hindered linkers (**1**–**4**) and not with the PC analogs with the bulkier linkers (**5**–**8**).

Catechin and epicatechin (**15** and **16**) reacted differently than the natural PC dimers, synthetic PC analogs, or EGCG, as they formed highly oligomerized species (SI, Figure S60, Table S1). Even after 26 h incubation, which was twice as long as with the other compounds, majority of the starting materials remained but a late eluting broad peak appeared in the chromatograms. These peaks were tentatively characterized as mixtures of oxidized oligomers (SI, Figure S60). The peak shape was broadened, likely because the oligomers eluted simultaneously during the washing period of the gradient. The oligomers of epicatechin were characterized up to nonamers, and the catechin oligomers were characterized up to undecamers (SI, Table S1). The characterization was tentative, but the mass errors were low, which provided convincing evidence of the elemental compositions and characterization. Ideally, extracted ion chromatograms would have provided more evidence that each compound was actually their own separate compound and not fragment ions of a single higher oligomer or cluster ions of smaller oligomers. Unfortunately, this could not be achieved because the oligomers coeluted during the wash segment. However, some of the lower oligomers eluted separately at earlier retention times, which confirmed the formation of individual oxidized oligomers. Interestingly, the oligomers were not simple oligomers with a single linkage between the building units. A pattern was observed where the degree of oxidation increased as the degree of oligomerization increased. With the highest characterized oligomers **15**_9_–H_12_ and **16**_11_–H_18_ (SI, Table S1), there were a difference of 12 and 18 hydrogens, respectively, compared to compounds which would only have a single new bond between each constituting (epi)catechin units. Again, the structural nature of the modification could not be established with mass spectrometry alone, but nonetheless, most of the constituting units in the oligomers had to have undergone further oxidation. The fact that **15** and **16** were capable to oligomerize in the PBS solution also supported the earlier conclusion that the M–H_2_ products of the PC analogs and PC oligomers corresponded to formation of new intramolecular bonds.

Overall, the stabilities and the complexities of the mixtures of products of the tested PC analogs varied considerably considering how little the compounds differed from one another. For instance, the PC analogs with less bulky linkage units, i.e., methylene (**1** and **2**) or carboxymethine linkers (**3** and **4**), yielded multiple products with increased number of oxygen, whereas the PC analogs with bulkier linkers, i.e., furanmethine and catecholmethine linkers (**5**–**8**), produced fewer products where only the number of hydrogens had decreased. The PC analogs with pyrogallolmethine linkers (**9** and **10**) were an exception to this, as they degraded practically instantly to complex mixtures products. The three-dimensional structures of the tested compounds certainly affected the possible formation of intramolecular linkages via oxidation by dictating which structural moieties of the (epi)catechin units were in close vicinity to one another to enable these reactions. The degradation of the tested PC analogs can naturally affect their biological properties, and the degradation could have either positive or negative effect on the biological activities. For instance, if further intramolecular bonds are formed between the building units, this could negatively affect the PPC. Similar phenomenon has been established to occur with galloyl glucoses and ellagitannins where the formation of hexahydroxydiphenoyl (HHDP) groups from galloyl groups negatively affects the PPC.^21^ Here, an additional C–C linkage between the B-rings would produce a structure very similar to that of an HHDP group. On the other hand, the formation of *o*-quinone containing products from catechol and pyrogallol groups could change the mode of interaction with proteins because the *o*-quinone structures would enable covalent bonding with proteins, which is irreversible compared to the weaker interactions.^51^ The fact that the tested PC analogs did degrade in a neutral PBS solution is something that needs to be considered in any further *in vivo* and *in vitro* experiments where similar PC analogs are studied.

The results obtained in this study encourage the further assessment of the biological activities of PA analogs. This study focused entirely on dimeric PC analogs, but for any practical applications, such as utilizing PC analogs as anthelmintics, higher oligomers and polymers would certainly be worth investigating. For instance, it would be interesting to further assess whether the more flexible methylene or substituted methine linkages in higher PC analogs would increase the PPC further still when compared to similarly sized natural PAs. This study can serve as motivation for further studies, as it was evident that the properties of the PC analogs were greatly affected by the selected aldehyde in their synthesis and by the linker unit they provided to the PC analogs. With dimeric compounds, it was shown that the PPC of PC analogs increased substantially compared to similarly sized natural PC dimers. The log *P* and, thereby, the hydrophobicity, could be controlled by selection of the linkage-unit, and the behavior in physiologically relevant conditions in PBS depended significantly on the linkage unit as well.

## Supporting Information Available

Characterization data of the PC analogs; 1D and 2D NMR spectra for each compound and assignation of the 1D spectra, as well as exact masses, molecular formulas, and UV absorption maxima of the PC analogs; dose–response models fitted to the PPC data, proportions of the precipitated PC analogs and BSA in the PPC experiment, and UV chromatograms of each PC analogue from the stability experiments at two different time points; data for accurate mass measurements and the elemental compositions of the products formed in the stability experiments (PDF)

## Abbreviations

3,4,5-TBA: 3,4,5-trihydroxybenzaldehyde
3,4-DBA: 3,4-dihydroxybenzaldehyde
COSY: correlated spectroscopy
DAD: diode array detector
ED_50_: effective dose 50 (concentration where 50% of maximal absorption is achieved)
EGCG: epigallocatechin-3-*O*-gallate
HESI: heated electrospray ionization
HMBC: heteronuclear multiple bond correlation
HPLC: high-performance liquid chromatography
HSQC: heteronuclear single quantum coherence
LC–MS: liquid chromatography–mass spectrometry
log *P*: logarithm of octanol–water partition coefficient
NMR: nuclear magnetic resonance
PA: proanthocyanidin
PBS: phosphate-buffered saline
PC: procyanidin
PPC: protein precipitation capacity
UPLC: ultraperformance liquid chromatography
