# Supplementary material for: Red Wine Inspired Chemistry: Hemisynthesis of Procyanidin Analogs and Determination of Their Protein Precipitation Capacity, Octanol–Water Partition, and Stability in Phosphate-Buffered Saline
Source: J Agric Food Chem. 2023 Dec 4;71(49):19832–44. doi: 10.1021/acs.jafc.3c06467 (PMC10722540; doi:10.1021/acs.jafc.3c06467)
Supplement: Supplementary file 1 — jf3c06467_si_001.pdf [file jf3c06467_si_001.pdf]

SUPPORTING INFORMATION

Red Wine Inspired Chemistry: Hemisynthesis of Procyanidin Analogs and Determination of Their Protein Precipitation Capacity, Octanol–Water Partition and Stability in Phosphate-Buffered Saline

Juuso Erik Laitila[a,*], Petri Tapani Tähtinen[a], Maarit Karonen[a], Juha-Pekka Salminen[a]

[a]Department of Chemistry, University of Turku, Turku, FI-20014, Finland

*E-mail: juerlai@utu.fi, Tel.: +358294505000

Characterization data of the PC analogs

Catechin–methylene–Catechin (1; Figure S1):

Yield 3%; UV: λ_{max} 203 nm, 230 nm (sh), 280 nm (23/77, acetonitrile/0.1% formic acid in water (v:v); HRMS: measured *m/z* 591.1520 ($[M-H]^-$), measured mass 592.1593 Da, exact mass 592.1581 Da, mass error 1.67 ppm, molecular formula $C_{31}H_{28}O_{12}$; ^1H NMR (500.08 MHz, $(CD_3)_2CO$, 278 K): δ 2.54 (dd, 2, *J* = 8.7, 16.4 Hz, H4α), δ 2.94 (dd, 2, *J* = 5.4, overlapped by solvent signal Hz, H4β), δ 3.65 (s, 1, H9), δ 4.08 (m, 2, H3), δ 4.16 (d, 2, *J* = 4.8 Hz, OH-3), δ 4.68 (d, 2, *J* = 8.2 Hz, H2), δ 5.98 (s, 2, H6), δ 6.81 (br, 4, H5'/H6'), δ 6.95 (br, 2, H2'); ^{13}C NMR (125.76 MHz, $(CD_3)_2CO$, 278 K): δ 16.45 (C9), δ 28.97 (C4), δ 67.64 (C3), δ 83.75 (C2), δ 97.02 (C6), δ 100.94 (C4a), δ 105.28 (C8), δ 115.36 (C2'), δ 115.88 (C5'), δ 120.25 (C6'), δ 130.72 (C1'), δ 145.71 (C3'), δ 146.07 (C4'), δ 152.35 (C8a), δ 155.31 (C5), δ 155.53 (C7).

Epicatechin–methylene–Epicatechin (2; Figure S6):

Yield: 3%; UV: λ_{max} 201 nm, 230 nm (sh), 279 nm (25/75, acetonitrile/0.1% formic acid in water (v:v); HRMS: measured *m/z* 591.1520 ($[M-H]^-$), measured mass 592.1593 Da, exact mass 592.1581 Da, mass error 2.02 ppm, molecular formula $C_{31}H_{28}O_{12}$; ^1H NMR (500.08 MHz, $(CD_3)_2CO$, 278 K): δ 2.79 (dd, 2, *J* = 2.9, 16.7 Hz, H4α), δ 2.89 (dd, 2, *J* = 5.4, 16.7, H4β), δ 3.82 (s, 1, H9), δ 3.90 (m, 2, H3), δ 4.25 (m, 2, OH-3), δ 5.03 (br, 2, H2), δ 5.94 (s, 2, H6), δ 6.82 (d, 2, *J* = 8.2, H5'), δ 6.92 (dd, 2, *J* = 1.8, 8.2 Hz, H2'); ^{13}C NMR (125.76 MHz, $(CD_3)_2CO$, 278 K): δ 16.31 (C9), δ 29.14 (C4), δ 66.43 (C3), δ 80.53 (C2), δ 96.87 (C6), δ 99.93 (C4a), δ 105.37 (C8), δ 115.23 (C2'), δ 115.63 (C5'), δ 119.48 (C6'), δ 131.25 (C1'), δ 145.46 (C3'), δ 146.61 (C4'), δ 152.47 (C8a), δ 155.48 (C5), δ 155.76 (C7).

Catechin–carboxymethine–catechin (3; Figure S11):

Yield: 13%; UV: λ_{max} 202 nm, 280 nm (14/86, acetonitrile/0.1% formic acid in water (v:v); HRMS: measured *m/z* 635.1415 ($[M-H]^-$), measured mass 636.1485 Da, exact mass 636.1491 Da, mass error 0.89 ppm, molecular formula $C_{32}H_{28}O_{14}$; ^1H NMR (500.08 MHz, $(CD_3)_2CO$, 278 K): δ 2.50 (dd, 1, *J* = 8.8, 16.2 Hz, H4α_A), δ 2.55 (dd, 1, *J* = 8.0, 16.2, H4α_B), δ 2.76 (dd, 1, *J* = 5.3, 16.2, H4β_B), δ 2.93 (dd, 1, *J* = 5.7, 16.2, H4β_A), δ 3.91 (m, 1, H3_B), δ 3.96 (m, 1, H3_A), δ 4.01 (d, 1, *J* = 4.8, OH-3_B),), δ 4.04 (d, 1, *J* = 4.4, OH-3_A), δ 4.29 (d, 1, *J* = 8.0, H2_A), δ 4.65 (d, 1, *J* = 7.2, H2_B), δ 5.95 (s, 1, H9), δ 6.00 (s, 1, H6_A), δ 6.07 (s, 1, H6_B), δ 6.53 (dd, 1, *J* = 1.9, 8.2, H6'$_B$), δ 6.62 (dd, 1, *J* = 2.0, 8.2, H6'$_A$), δ 6.66 (d, 1, *J* = 8.2, H5'$_B$), δ 6.72 (d, 1, *J* = 8.2, H5'$_A$), δ 6.79 (d, 1, *J* = 2.0, H2'$_A$), δ 6.86 (d, 1, *J* = 1.9, H2'$_B$); ^{13}C NMR (125.76 MHz, $(CD_3)_2CO$, 278 K): δ 28.28 (C4_B), δ 29.19 (C4_A), δ 36.17 (C9), δ 68.21 (C3_A and C3_B, two overlapping signals), δ 82.63 (C2_A), δ 82.94 (C2_B), δ 96.35 (C6_B), δ 96.92 (C6_A), δ 100.63 (C4a$_A$), δ 101.08 (C4a$_B$), δ 105.96 (C8_A/C8_B), δ 106.01 (C8_A/C8_B), δ 114.78 (C2'$_A$), δ 115.16 (C2'$_B$), δ 115.52 (C5'$_A$), δ 115.63 (C5'$_B$), δ 119.64 (C6'$_B$), δ 120.17 (C6'$_A$), δ 131.60 (C1'$_B$), δ 131.74 (C1'$_A$), δ 145.43 (C3'$_A$/C3'$_B$/C4'$_A$/C4'$_B$, two overlapping signals), δ 145.53 (C3'$_A$/C3'$_B$/C4'$_A$/C4'$_B$), δ 145.57 (C3'$_A$/C3'$_B$/C4'$_A$/C4'$_B$), δ 153.84 (C8_A/C8_B), δ 153.90 (C8_A/C8_B), δ 154.34 (C7_B), δ 155.38 (C5_A), δ 155.52 (C5_B), δ 156.02 (C7_A), δ 174.53 (C10).

Epicatechin–carboxymethine–epicatechin (4; Figure S16):

Yield: 18%; UV: λ_{max} 200 nm, 279 nm (18/82, acetonitrile/0.1% formic acid in water (v:v); HRMS: measured *m/z* 635.1416 ($[M-H]^-$), measured mass 636.1489 Da, exact mass 636.1479 Da, mass error 1.52 ppm, molecular formula $C_{32}H_{28}O_{14}$; ^1H NMR (500.08 MHz, $(CD_3)_2CO$, 278 K): δ 2.64 (dd, 1, *J* = 4.4, 16.4 Hz, H4α_A), δ 2.73 (dd, 1, *J* = 3.2 / 16.4, H4α_B), δ 2.84 (dd, 1, *J* = 4.8, 16.4, H4β_B), δ 2.87 (dd, 1, *J* = 4.8, 16.4, H4β_A), δ 3.60 (d, 1, *J* = 5.3, OH-3_A), δ 3.64 (d, 1, *J* = 5.5, OH-3_B), δ 4.15 (m, 2, H3_A and H3_B, overlapping signals), δ 4.72 (br, 1,H2_B), δ 4.93 (br, 1, H2_B), δ 6.03 (s, 1, H6_B), δ 6.05 (s, 1, H6_A), δ 6.07 (s, 1, H9), δ 6.63 (d, 1, *J* = 2.2, H5'$_A$), δ 6.69 (dd, 1, *J* = 1.8, 8.2, H6'$_A$), δ 6.72 (d, 1, *J* = 8.2, H6'$_B$), δ 6.78 (dd, 1, *J* = 1.8, 8.2, H6'$_B$), δ 6.99 (d, 1, *J* = 1.9, H2'$_A$), δ 7.01 (d, 1, *J* = 1.9, H2'$_B$); ^{13}C NMR (125.76 MHz, $(CD_3)_2CO$, 278 K): δ 28.46 (C4_A), δ 29.30 (C4_B), δ 36.20 (C9), δ

66.49 ($C3_B$), δ 66.75 ($C3_A$), δ 79.81 ($C2_B$), δ 79.70 ($C2_A$), δ 96.60 ($C6_A$), δ 96.99 ($C6_B$), δ 100.08 ($C4a_B$), δ 100.15 ($C4a_A$), δ 106.07 ($C8_A$ and $C8_B$, two signals overlapping), δ 115.14 (C2'$_A$), δ 115.19 (C2'$_B$), δ 115.27 (C5'$_B$), δ 115.32 (C5'$_A$), δ 119.30 (C6'$_B$), δ 119.46 (C6'$_A$), δ 131.46 (C1'$_A$), δ 131.76 (C1'$_B$), δ 145.15 (C3'$_A$/C3'$_B$/C4'$_A$/C4'$_B$, overlapping signals), δ 145.18 (C3'$_A$/C3'$_B$/C4'$_A$/C4'$_B$, overlapping signals), δ 153.88 ($C8a_A$/$C8a_B$), δ 153.91 ($C8a_A$/$C8a_B$), δ 154.85 ($C7_A$/ $C7_B$), δ 155.70 ($C5_A$), δ 155.76 ($C5_B$), δ 155.85 ($C7_A$/ $C7_B$), δ 174.68 (C10).

Catechin–furanmethine–catechin (5; Figure S21):

Yield: 14%; UV: λ_{max} 202 nm, 280 nm (26/74, acetonitrile/0.1% formic acid in water (v:v); HRMS: measured *m/z* 657.1626 ($[M{-}H]^-$), measured mass 658.1699 Da, exact mass 658.1686 Da, mass error 1.88 ppm, molecular formula $C_{35}H_{30}O_{13}$; ^{1}H NMR (500.08 MHz, $(CD_3)_2CO$, 278 K): δ 2.52 (dd, 1, *J* = 8.2, 16.2 Hz, H4α_A), δ 2.57 (dd, 1, *J* = 8.0 / 16.2, H4α_B), δ 2.78 (dd, 1, *J* = 5.2, 16.2, H4β_B), δ 2.90 (dd, 1, *J* = 5.7, 16.2, H4β_A), δ 3.92 (m, 1, $H3_B$), δ 4.00 (m, 1, $H3_A$), δ 4.00 (d, 1, *J* = 4.6, OH-3_B), δ 4.11 (m, 1, *J* = 4.3 Hz, OH-3_A), δ 4.33 (d, 1, *J* = 7.8 Hz, $H2_A$), δ 4.60 (d, 1, *J* = 7.1 Hz, $H2_B$), δ 5.71 (m, 1, H14), δ 6.02 (s, 1, $H6_A$), δ 6.05 (s, 1, $H6_B$), δ 6.11 (m, 1, H13), δ 6.38 (s, 1, H9), δ 6.47 (dd (br), 1, *J* = 8.1 Hz, H6'$_B$), δ 6.55 (dd, 1, *J* = 1.9, 8.1, H6'$_A$), δ 6.64 (d, 1, *J* = 8.1, H5'$_B$), δ 6.67 (dd, 1, *J* = 8.1, H5'$_A$), δ 6.73 (d, 1, *J* = 1.9, H2'$_A$), δ 6.81 (d, 1, *J* = 1.7, H2'$_B$), δ 7.27 (m, 1, H12); ^{13}C NMR (125.76 MHz, $(CD_3)_2CO$, 278 K): δ 28.38 ($C4_B$), δ 28.89 ($C4_A$), δ 30.41 (C9), δ 67.88 ($C3_A$), δ 68.13 ($C3_B$), δ 82.53 ($C2_A$), δ 82.89 ($C2_B$), δ 96.82 ($C6_A$), δ 96.93 ($C6_B$), δ 100.90 ($C4a_A$), δ 101.01 ($C4a_B$), δ 105.91 (C14), δ 106.88 ($C8_B$), δ 107.26 ($C8_A$), δ 110.63 (C13), δ 114.86 (C2'$_A$), δ 115.09 (C2'$_B$), δ 115.45 (C5'$_A$/C5'$_B$), δ 115.49 (C5'$_A$/C5'$_B$), δ 119.70 (C6'$_B$), δ 120.03 (C6'$_A$), δ 131.44 (C1'$_B$), δ 131.50 (C1'$_A$), δ 141.42 (C12), δ 145.32 (C3'$_A$/C3'$_B$), δ 145.36 (C3'$_A$/C3'$_B$), δ 145.53 (C4'$_A$ and C4'$_B$, two overlapping signals), δ 153.88 ($C8a_A$/$C8a_B$), δ 154.01 ($C8a_A$/$C8a_B$), δ 155.08 ($C5_A$/$C5_B$/$C7_A$/$C7_A$), δ 155.43 ($C5_A$/$C5_B$/$C7_A$/$C7_A$), δ 155.48 ($C5_A$/$C5_B$/$C7_A$/$C7_A$), δ 155.90 ($C5_A$/$C5_B$/$C7_A$/$C7_A$), δ 157.00 (C10).

Epicatechin–furanmethine–epicatechin (6; Figure S26):

Yield: 14%; UV: λ_{max} 201 nm, 215 nm (sh), 280 nm (27/73, acetonitrile/0.1% formic acid in water (v:v); HRMS: measured *m/z* 657.1615 ($[M-H]^-$), measured mass 658.1688 Da, exact mass 658.1686 Da, mass error 0.21 ppm, molecular formula $C_{35}H_{30}O_{13}$; ^1H NMR (500.08 MHz, $(CD_3)_2CO$, 278 K): δ 2.63 (dd, 1, *J* = 4.5, 16.5 Hz, $H4\alpha_A$), δ 2.67 (dd, 1, *J* = 4.8, 16.5, $H4\alpha_B$), δ 2.82 (dd, 1, *J* = 4.8, 16.5, $H4\beta_B$), δ 2.88 (dd, 1, *J* = 4.7, 16.5, $H4\beta_A$), δ 3.64 (m, 1, OH-3_B), δ 3.65 (m, 1, OH-3_A), δ 4.14 (m, 2, $H3_A/H3_B$), δ 4.68 (br, 1, $H2_B$), δ 4.88 (br, 1, $H2_A$), δ 5.79 (m, 1, H14), δ 6.01 (s, 1, $H6_A$), δ 6.06 (s, 1, $H6_B$), δ 6.14 (m, 1, H13), δ 6.49 (s, 1, H9), δ 6.57 (m, 2, H5'$_A$/ H6'$_A$), δ 6.69 (m, 2, H5'$_B$/ H6'$_B$), δ 6.88 (br, 1, H2'$_A$), δ 6.90 (br, 1, H2'$_B$), δ 7.28 (s, 1, H12); ^{13}C NMR (125.76 MHz, $(CD_3)_2CO$, 278 K): δ 28.45 ($C4_A$), δ 28.93 ($C4_B$), δ 30.52 (C9), δ 66.37 ($C3_B$), δ 66.78 ($C3_A$), δ 79.65 ($C2_B$), δ 79.77 ($C2_A$), δ 96.89 ($C6_A$ and $C6_B$, two overlapping signals), δ 100.23 ($C4a_A$), δ 100.32 ($C4a_B$), δ 106.16 (C14), δ 107.36 ($C8_A$), δ 107.04 ($C8_B$), δ 110.66 (C13), δ 115.11 (C2'$_A$/ C2'$_B$/ C5'$_A$/ C5'$_B$), δ 115.14 (C2'$_A$/ C2'$_B$/ C5'$_A$/ C5'$_B$), δ 115.19 (C2'$_A$/ C2'$_B$/ C5'$_A$/ C5'$_B$, two overlapping signals), δ 119.41 (C6'$_B$), δ 119.64 (C6'$_A$), δ 131.34 (C1'$_A$), δ 131.55 (C1'$_B$), δ 141.50 (C12), δ 145.07 (C3'$_A$/C3'$_B$/C4'$_A$/C4'$_B$, two overlapping signals), δ 145.10 (C3'$_A$/C3'$_B$/C4'$_A$/C4'$_B$), δ 145.16 (C3'$_A$/C3'$_B$/C4'$_A$/C4'$_B$), δ 154.02 ($C8a_A/C8a_B$), δ 154.05 ($C8a_A/C8a_B$), δ 155.33 ($C5_A/C5_B/C7_A/C7_A$), δ 155.64 ($C5_A/C5_B/C7_A/C7_A$, two overlapping signals), δ 155.74 ($C5_A/C5_B/C7_A/C7_A$), δ 157.14 (C10).

Catechin–catecholmethine–catechin (7; Figure S31):

Yield: 9%; UV: λ_{max} 201 nm, 280 nm (19/81, acetonitrile/0.1% formic acid in water (v:v); HRMS: measured *m/z* 699.1728 ($[M-H]^-$), measured mass 700.1801 Da, exact mass 700.1792 Da, mass error 1.223 ppm, molecular formula $C_{37}H_{32}O_{14}$; ^1H NMR (500.08 MHz, $(CD_3)_2CO$, 278 K): δ 2.55 (dd, 1, *J* = 8.3, 16.8 Hz, $H4\alpha_A$), δ 2.56 (dd, 1, *J* = 9.1, 16.6, $H4\alpha_B$), δ 2.82 (dd (br), 1, *J* = –, 16.1, $H4\beta_B$), δ 2.92 (dd, 1, *J* = 4.5, 16.2, $H4\beta_A$), δ 3.84 (br, 1, $H3_B$), δ 4.02 (m, 1, $H3_A$), δ 4.02 (br, 1, OH-3_A/OH-3_B), δ 4.13 (br, 1, OH-3_A/OH-3_B), δ 4.39 (d, 1, $H2_A$), δ 4.54 (d, 1, $H2_B$), δ 6.02 (br, 2, $H6_A$/ $H6_B$), δ 6.29 (br, 1, H6'$_A$/H6'$_B$), δ 6.37 (m, 2, H9 and H15, two signals overlapping), δ 6.45 (m, 1, H5'$_A$/

H5'$_B$), δ 6.51 (br, 1, H11), δ 6.52 (m, 1, H6'$_A$/ H6'$_B$), δ 6.60 (m, 1, H14), δ 6.72 (br, 1, H2'$_A$/H2'$_B$); ^{13}C NMR (125.76 MHz, $(CD_3)_2CO$, 278 K): δ 28.93 (C4$_A$ and C4$_B$, two signals overlapping), δ 34.26 (C9), δ 67.81 (C3$_A$), δ 68.37 (C3$_B$), δ 82.44 (C2$_A$), δ 83.04 (C2$_B$), δ 96.97 (C6$_A$/C6$_B$), δ 97.07 (C6$_A$/C6$_B$), δ 100.94 (C4a$_A$), δ 101.18 (C4a$_B$), δ 108.03 (C8$_A$/ C8$_B$), δ 109.04 (C8$_A$/ C8$_B$), δ 114.86 (C2'$_A$/ C2'$_B$), δ 115.03 (C2'$_A$/ C2'$_B$), δ 115.15 (C11), δ 115.52 (C5'$_A$/ C5'$_B$), δ 115.56 (C5'$_A$/ C5'$_B$), δ 115.64 (C14), δ 119.19 (C15), δ 119.44 (C6'$_A$/C6'$_B$), δ 120.19 (C6'$_A$/C6'$_B$), δ 131.11 (C1'$_A$/C1'$_B$), δ 131.30 (C1'$_A$/C1'$_B$), δ 134.93 (C10), δ 143.44 (C13), δ 145.06 (C12), δ 145.31 (C3'$_A$/C3'$_B$/C4'$_A$/C4'$_B$), δ 145.36 (C3'$_A$/C3'$_B$/C4'$_A$/C4'$_B$), δ 145.50 (C3'$_A$/C3'$_B$/C4'$_A$/C4'$_B$), δ 145.60 (C3'$_A$/C3'$_B$/C4'$_A$/C4'$_B$), δ 153.91 (C8a$_A$ and C8a$_A$, two signals overlapping), 155.29 (C5$_A$/C5$_B$/C7$_A$/C7$_B$), 155.33 (C5$_A$/C5$_B$/C7$_A$/C7$_B$), 155.45 (C5$_A$/C5$_B$/C7$_A$/C7$_B$), 156.09 (C5$_A$/C5$_B$/C7$_A$/C7$_B$).

Epicatechin–catecholmethine–epicatechin (8; Figure S36):

Yield: 10%; UV: λ_{max} 200 nm, 280 nm (22/78, acetonitrile/0.1% formic acid in water (v:v); HRMS: measured *m/z* 699.1729 ([M−H]$^-$), measured mass 700.1802 Da, exact mass 700.1792 Da, mass error 1.423 ppm, molecular formula $C_{37}H_{32}O_{14}$; ^{1}H NMR (500.08 MHz, $(CD_3)_2CO$, 278 K): δ 2.71 m, 1, H4α$_A$), δ 2.74 (m, 1, H4α$_B$), δ 2.86 (m, 1, H4β$_A$), δ 2.90 (m, 1, H4β$_B$), δ 3.38 (m, 1, OH-3$_B$), δ 3.72 (m, 1, OH-3$_A$), δ 4.11 (m, 1, H3$_B$), δ 4.20 (m, 1, H3$_A$), δ 4.82 (m, 1, H2$_A$), δ 4.85 (m, 1, H2$_B$), δ 6.01 (s, 1, H6$_A$/ H6$_B$), δ 6.02 (s, 1, H6$_A$/ H6$_B$), δ 6.23 (br, 1, H6'$_B$), δ 6.46 (br, 1, H9), δ 6.51 (d, 1, *J* = 8.2 Hz, H15), δ 6.56 (d, 1, *J* = 8.2 Hz, H5'$_B$), δ 6.62 (br, 1, H11), δ 6.67 (m, 2, H14 and H2'$_B$), δ 6.69 (m, 1, H5'$_A$), δ 6.92 (br, 1, H2'$_A$); ^{13}C NMR (125.76 MHz, $(CD_3)_2CO$, 278 K): δ 28.91 (C4$_A$), δ 29.05 (C4$_B$), δ 34.36 (C9), δ 66.40 (C3$_A$), δ 66.71 (C3$_B$), δ 79.71 (C2$_A$), δ 79.92 (C2$_B$), δ 97.02 (C6$_A$/C6$_B$), δ 97.12 (C6$_A$/C6$_B$), δ 100.21 (C4a$_A$/C4a$_B$), δ 100.29 (C4a$_A$/C4a$_B$), δ 107.92 (C8$_A$/ C8$_B$), δ 108.92 (C8$_A$/ C8$_B$), δ 114.45 (C2'$_B$), δ 114.96 (C2'$_A$), δ 115.31 (C5'$_A$/ C5'$_B$/ C11/C14), δ 115.36 (C5'$_A$/ C5'$_B$/ C11/C14), δ 115.41 (C5'$_A$/ C5'$_B$/ C11/C14), δ 115.78 (C5'$_A$/ C5'$_B$/ C11/C14), δ 119.45 (C6'$_B$), δ 119.51 (C6'$_A$ and C15, two overlapping signals), δ 131.01 (C1'$_B$), δ 131.34 (C1'$_A$), δ 134.76 (C10), δ 143.54 (C13), δ 145.03 (C3'$_B$), δ 145.11 (C3'$_A$/C4'$_A$/C4'$_B$, two signals overlapping), δ 145.23

($C3'_A/C4'_A/C4'_B$), δ 145.42 ($C3'_A/C4'_A/C4'_B$), δ 153.70 ($C8a_A$ and $C8a_A$, two signals overlapping), δ 153.77 ($C8a_A$ and $C8a_A$), 155.65 ($C5_A/C5_B/C7_A/C7_B$), 155.70 ($C5_A/C5_B/C7_A/C7_B$), 155.87 ($C5_A/C5_B/C7_A/C7_B$, two signals overlapping).

Catechin–pyrogallolmethine–catechin (9; Figure S41):

Yield: 6%; UV: λ_{max} 201 nm, 279 nm (16/84, acetonitrile/0.1% formic acid in water (v:v); HRMS: measured *m/z* 715.1675 ($[M-H]^-$), measured mass 716.1748 Da, exact mass 716.1741 Da, mass error 0.92 ppm, molecular formula $C_{37}H_{32}O_{15}$; ^1H NMR (500.08 MHz, $(CD_3)_2CO$, 278 K): δ 2.54 (dd, 1, *J* = 8.3, 16.3 Hz, $H4\alpha_A$), δ 2.56 (dd, 1, *J* = 8.3, 16.3 Hz, $H4\alpha_B$), δ 2.81 (dd (br), 1, *J* = 16.3 Hz, $H4\beta_A$), δ 2.92 (dd, 1, *J* = 5.7, 16.3 Hz, $H4\beta_B$), δ 3.83 (br, 1, $H3_B$), δ 4.01 (m, 2, $H3_A$ and $OH\text{-}3_B$), δ 4.13 (br, 1, $OH\text{-}3_A$), δ 4.37 (br, 1, $H2_A$), δ 4.55 (br, 1, $H2_B$), δ 6.00 (br, 2, $H6_A$ and $H6_B$), δ 6.10 (s, 2, H11 and H15), δ 6.32 (br, 2, H9 and $H6'_B$), δ 6.53 (br, 1, $H6'_A$/ $H6'_B$), δ 6.61 (br, 1, $H5'_A$/ $H5'_B$), δ 6.65 (br, 1, $H5'_A$/ $H5'_B$), δ 6.72 (br, 1, $H2'_A$/ $H2'_B$); ^{13}C NMR (125.76 MHz, $(CD_3)_2CO$, 278 K): δ 28.97 ($C4_A$ and $C4_B$, two overlapping signals), δ 34.36 (C9), δ 67.83 ($C3_A$), δ 68.37 ($C3_B$), δ 82.39 ($C2_A$), δ 82.98 ($C2_B$), δ 96.94 ($C6_A/C6_B$), δ 97.05 ($C6_A/C6_B$), δ 100.90 ($C4a_A$), δ 101.18 ($C4a_B$), δ 106.92 (C11 and C15, two signals overlapping), δ 108.01 ($C8_A/C8_B$), δ 109.07 ($C8_A/C8_B$), δ 114.82 ($C2'_A/C2'_B$), δ 114.99 ($C2'_A/C2'_B$), δ 115.55 ($C6'_A/C6'_B$), δ 115.66 ($C6'_A/C6'_B$, δ 119.46 ($C5'_A/C5'_B$), δ 120.25 ($C5'_A/C5'_B$), δ 131.12 ($C1'_A/C1'_B$/C10), δ 131.31 ($C1'_A/C1'_B$/C10), δ 131.40 ($C1'_A/C1'_B$/C10), δ 145.04 ($C3'_A$/ $C3'_B/C4'_A/C4'_B$/C12/C14), δ 145.30 ($C3'_A/C3'_B/C4'_A/C4'_B$/C12/C14), δ 145.50 ($C3'_A$/ $C3'_B/C4'_A/C4'_B$/C12/C14), δ 145.60 ($C3'_A$/ $C3'_B/C4'_A/C4'_B$/C12/C14), δ 146.12 (C13), δ 153.92 ($C8a_A$ and $C8a_A$, two signals overlapping), 155.25 ($C5_A/C5_B/C7_A/C7_B$), 155.29 ($C5_A/C5_B/C7_A/C7_B$), 155.42 ($C5_A/C5_B/C7_A/C7_B$), 156.01 ($C5_A/C5_B/C7_A/C7_B$).

Epicatechin–pyrogallolmethine–epicatechin (10; Figure S46):

Yield: 13%; UV: λ_{max} 201 nm, 278 nm (19/81, acetonitrile/0.1% formic acid in water (v:v); HRMS: measured *m/z* 715.1681 ($[M-H]^-$), measured mass 716.1754 Da, exact mass 716.1741 Da, mass error 1.75 ppm, molecular formula $C_{37}H_{32}O_{15}$; ^{1}H NMR (500.08 MHz, $(CD_3)_2CO$, 278 K): δ 2.70 (m, 1, H4α_A), δ 2.73 (m, 1, H4α_B), δ 2.86 (m, 1, H4β_B), δ 2.89 (m, 1, H4β_A), δ 3.33 (br, 1, OH-3$_A$), δ 3.71 (br, 1, OH-3$_B$), δ 4.11 (br, 1, H3$_A$), δ 4.20 (br, 1, H3$_B$), δ 4.81 (br, 1, H2$_B$), δ 4.86 (br, 1, H2$_A$), δ 5.99 (s, 2, H6$_A$ and H6$_B$), δ 6.22 (s, 2, H11 and H15), δ 6.30 (br, 1, H6'$_A$/H6'$_B$), δ 6.42 (s, 1, H9), δ 6.57 (d, 1, *J* = 8.2 Hz, H5'$_A$/ H5'$_B$), δ 6.68 (br, 3, H2'$_A$/ H2'$_B$ and H5'$_A$/ H5'$_B$ and H6'$_A$/ H6'$_B$), δ 6.90 (br, 1, H2'$_A$/ H2'$_B$); ^{13}C NMR (125.76 MHz, $(CD_3)_2CO$, 278 K): δ 28.87 (C4$_A$/C4$_B$), δ 29.02 (C4$_A$/C4$_B$), δ 34.49 (C9) δ 66.41 (C3$_B$), δ 66.75 (C3$_A$), δ 79.63 (C2$_B$), δ 79.83 (C2$_A$), δ 96.96 (C6$_A$/C6$_B$), δ 97.09 (C6$_A$/C6$_B$), δ 100.19 (C4a$_A$ and C4a$_B$, two overlapping signals), δ 107.21 (C11 and C15, two overlapping signals), δ 108.97 (C8$_A$ and C8$_B$, two overlapping signals), δ 114.43 (C2'$_A$/C2'$_B$), δ 114.91 (C2'$_A$/C2'$_B$), δ 115.33 (C6'$_A$/C6'$_B$), δ 115.47 (C6'$_A$/C6'$_B$), δ 119.41 (C5'$_A$/C5'$_B$), δ 119.47 (C5'$_A$/C5'$_B$), δ 131.10 (C1'$_A$/C1'$_B$/C10), δ 131.29 (C1'$_A$/C1'$_B$/C10), δ 131.33 (C1'$_A$/C1'$_B$/C10), δ 145.02 (C3'$_A$/C3'$_B$/C4'$_A$/C4'$_B$/C12/C14), δ 145.06 (C3'$_A$/C3'$_B$/C4'$_A$/C4'$_B$/C12/C14), δ 145.14 (C3'$_A$/C3'$_B$/C4'$_A$/C4'$_B$/C12/C14), δ 145.22 (C3'$_A$/C3'$_B$/C4'$_A$/C4'$_B$/C12/C14), δ 146.30 (C13), δ 153.78 (C8a$_A$ and C8a$_A$, two overlapping signals), 155.61 (C5$_A$/C5$_B$/C7$_A$/C7$_B$), 155.65 (C5$_A$/C5$_B$/C7$_A$/C7$_B$), 155.85 (C5$_A$/C5$_B$/C7$_A$/C7$_B$, two signals overlapping).

The numbering of the structures of the PC analogs is presented with the ^{1}H NMR spectra in supplementary figures S1, S6, S11, S16, S21, S26, S31, S36, S41 and S46. Overall, the ^{1}H and especially the ^{13}C NMR spectra of the PC analogs resembled closely the ^{1}H and ^{13}C NMR spectra of catechin, epicatechin and PC dimers that have been reported in the literature, which helped to verify the assignations of the spectra of the PC analogs.[1,2] Additionally, the spectra of catechin–carboxymethine–catechin (**3**) matched the reported spectra of the same compound in the literature.[3] The ^{1}H NMR and spectra of compounds **1**–**6** were fully assigned and the ^{13}C NMR spectra were almost fully assigned with the main exceptions being in some of the signals of the carbons that were attached to hydroxyl groups in A and B rings. These signals could not always be assigned to specific catechin or epicatechin unit due to the close proximity or overlapping of signals, but their positions in the catechin or epicatechin units were identified. The assignation of the spectra began by identifying the H4 signals of the C-ring from the multiplicity edited HSQC spectra based on their negative phase. From there on, the signals of C and B rings were assigned separately for both monomeric units using COSY, HSQC and HMBC spectra. The important C8a carbon was characterized by HMBC correlations from H4 and H2 to C8a. Then, the C8a was observed to correlate with the hydrogen in the linking methylene or methine groups in the HMBC spectra, which confirmed the linkage position. The rest of the signals of the A-ring and the linkage unit were then identified using HMBC and HSQC correlations. The ^{1}H NMR and ^{13}C NMR spectra of **1** and **2** displayed only one set of signals because there is a symmetry plane at the methylene group. All other PC analogs displayed separate signals for both monomeric units due to the compounds being helically chiral.

The assignation of the ^{1}H and ^{13}C NMR spectra of compounds **7**–**10** was affected by the fact that the signals in the ^{1}H NMR spectra were broadened. The NMR experiments were performed at 5 °C to prevent degradation of the compounds during the measurements, but a wider temperature range was tested with compound **8** by measuring the ^{1}H NMR spectra in several temperatures in between 5–45 °C to improve the quality of the spectra. However, the temperature had only a negligible effect on the peak shapes. The broadened peak shapes were most likely due to some dynamic intramolecular process such as equilibrium between two or more major conformations. The signals in the 1D spectra

were assigned to specific atoms of the catechin or epicatechin units, but in most cases the signals of the two monomeric units could not be separated from one another due to lack of resolution and proximity of certain signals, especially in the ^{13}C NMR spectra. Despite the broadened peaks, the compounds **8** and **10** could be confirmed to be C8–C8 linked as their HMBC spectra showed correlation between the methine group hydrogen and C8a carbons. However, the position of the catecholmethine and pyrogallolmethine groups in **7** and **9** could not be confirmed from the HMBC spectra because the hydrogen of the methine group did not show any HMBC correlations and nor did the carbon of the methine group. Compounds **7** and **9** had fewer HMBC correlations in general in comparison to other compounds. For instance, H2 showed correlations to carbons in B-ring with all other compounds but in compounds **7** and **9** the H2 did not show any HMBC correlations. The lack of HMBC correlations could have been related to the decreased sensitivity due to the broadened peaks, or to coupling constants being affected by the intramolecular processes. Nonetheless, the ^{1}H and ^{13}C NMC spectra of **7** and **9** were almost identical to corresponding spectra of **8** and **10** and to other PC analogs as well, and because all other PC analogs were confirmed to be C8–C8 linked, and the literature data of similar reactions support the results of this study regarding the primary linkage position, therefore, the compounds **7** and **9** were assumed to be C8–C8 linked as well.

References

(1) Khan, M. L.; Haslam, E.; Williamson, M. P. Structure and Conformation of the Procyanidin B-2 Dimer. *Magnetic Resonance in Chemistry* **1997**, *35* (12), 854–858. https://doi.org/10.1002/(sici)1097-458x(199712)35:12<854::aid-omr184>3.0.co;2-n.

(2) Davis, A. L.; Cai, Y.; Davies, A. P.; Lewis, J. R. 1H and 13C NMR Assignments of Some Green Tea Polyphenols. *Magnetic Resonance in Chemistry* **1996**, *34* (11), 887–890. https://doi.org/10.1002/(SICI)1097-458X(199611)34:11<887::AID-OMR995>3.0.CO;2-U.

(3) Fulcrand, H.; Cheynier, V.; Oszmianski, J.; Moutounet, M. An Oxidized Tartaric Acid Residue as a New Bridge Potentially Competing with Acetaldehyde in Flavan-3-Ol Condensation. *Phytochemistry* **1997**, *46*, 223–227.

Supplementary figures and tables

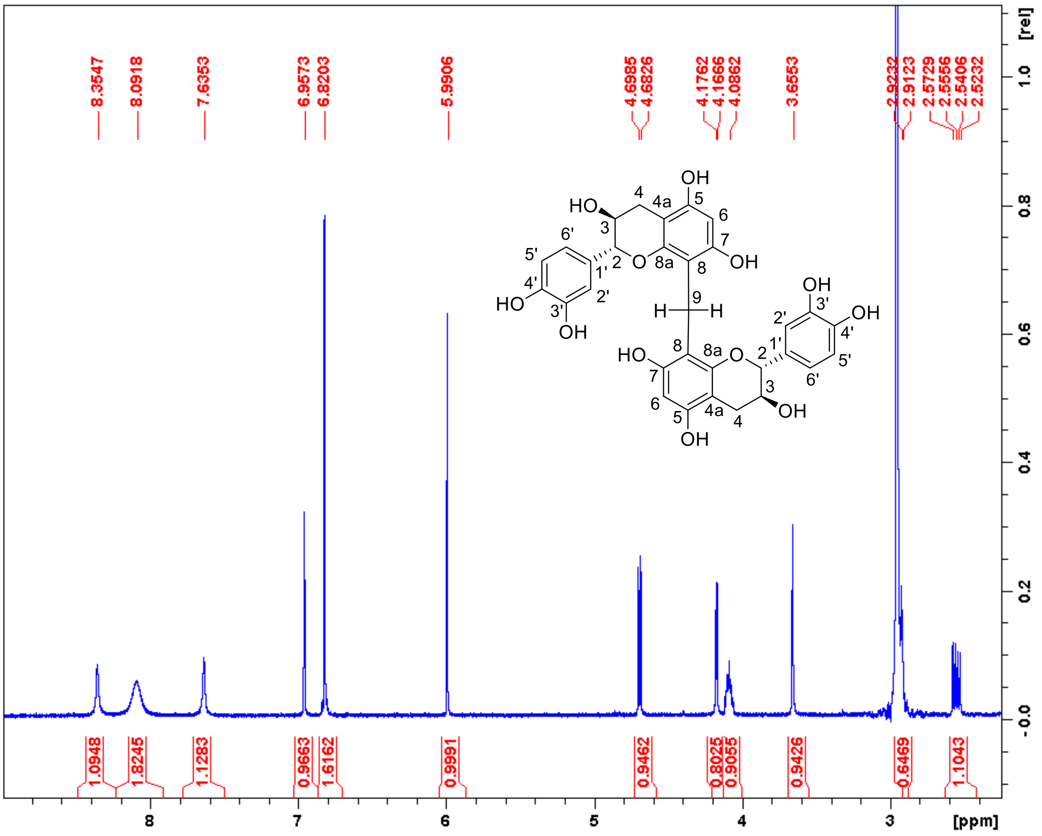

Figure S1. ^{1}H NMR spectrum and structure of catechin–methylene–catechin (**1**).

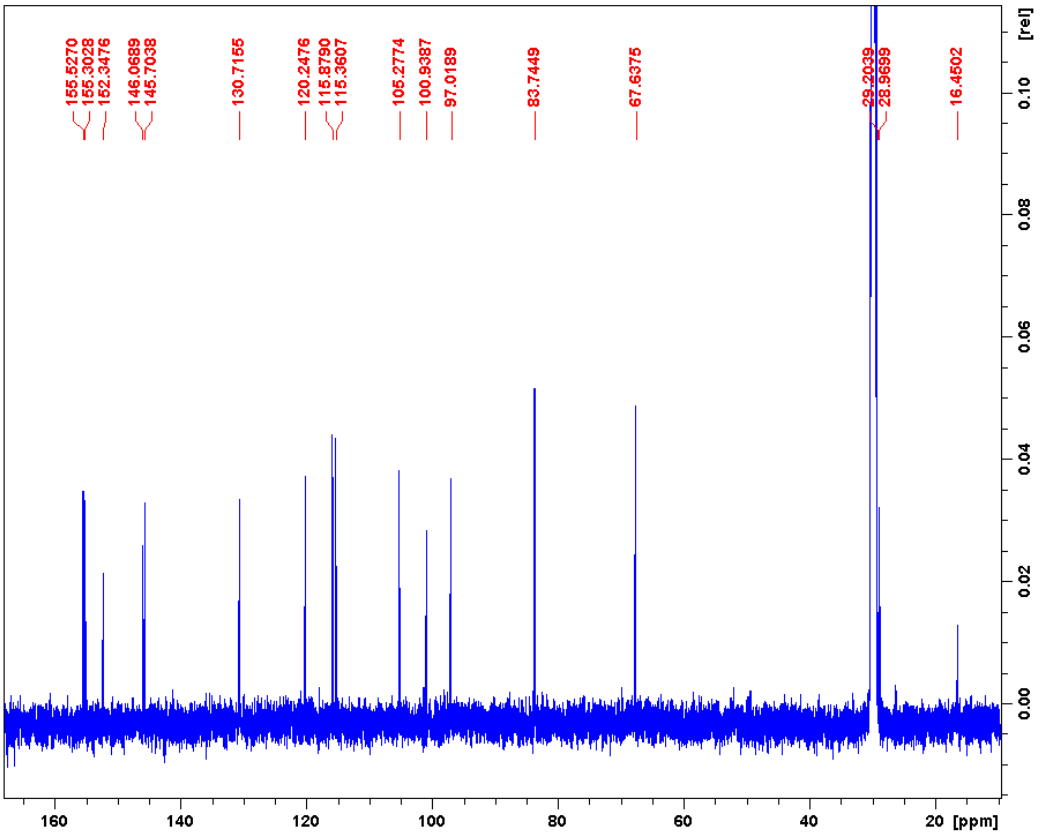

Figure S2. ^{13}C NMR spectrum of catechin–methylene–catechin (**1**).

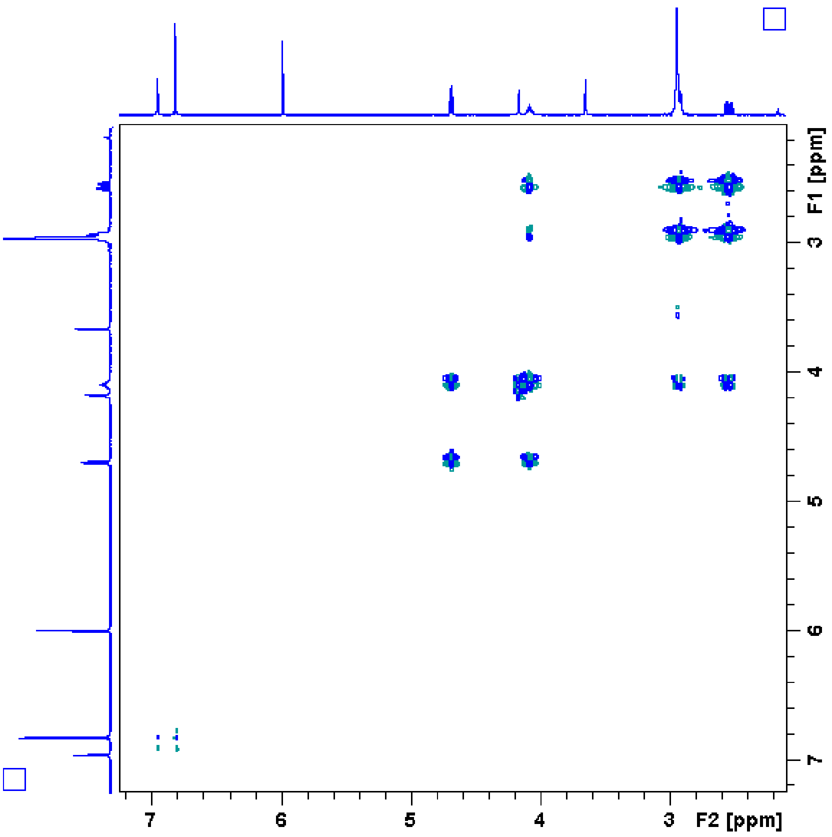

Figure S3. ^{1}H, ^{1}H COSY spectrum of catechin–methylene–catechin (**1**).

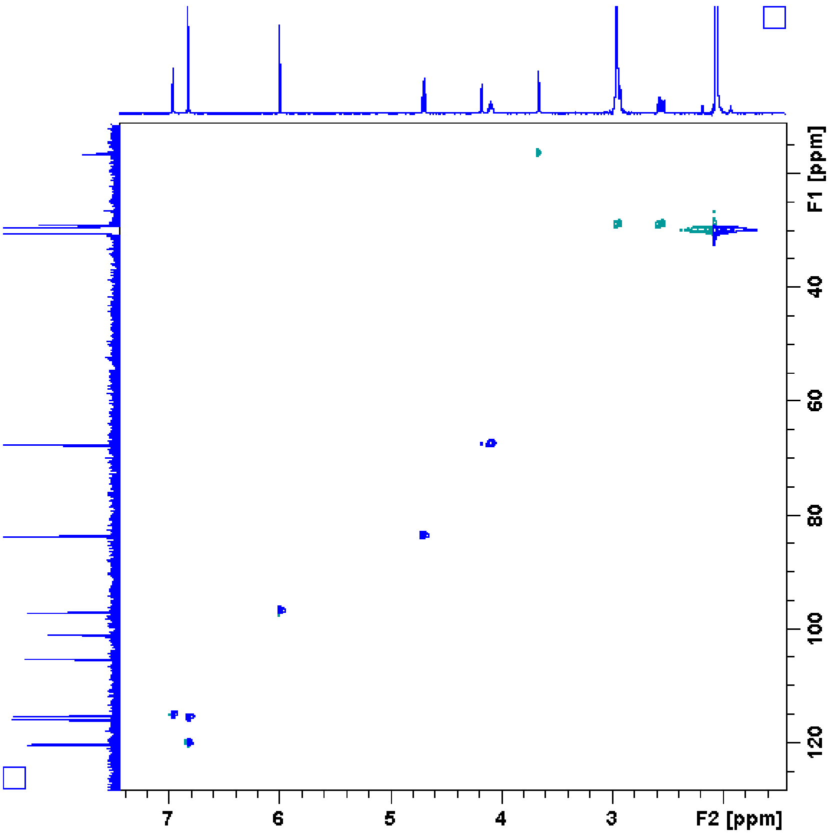

Figure S4. ^{1}H, ^{13}C HSQC spectrum of catechin–methylene–catechin (**1**).

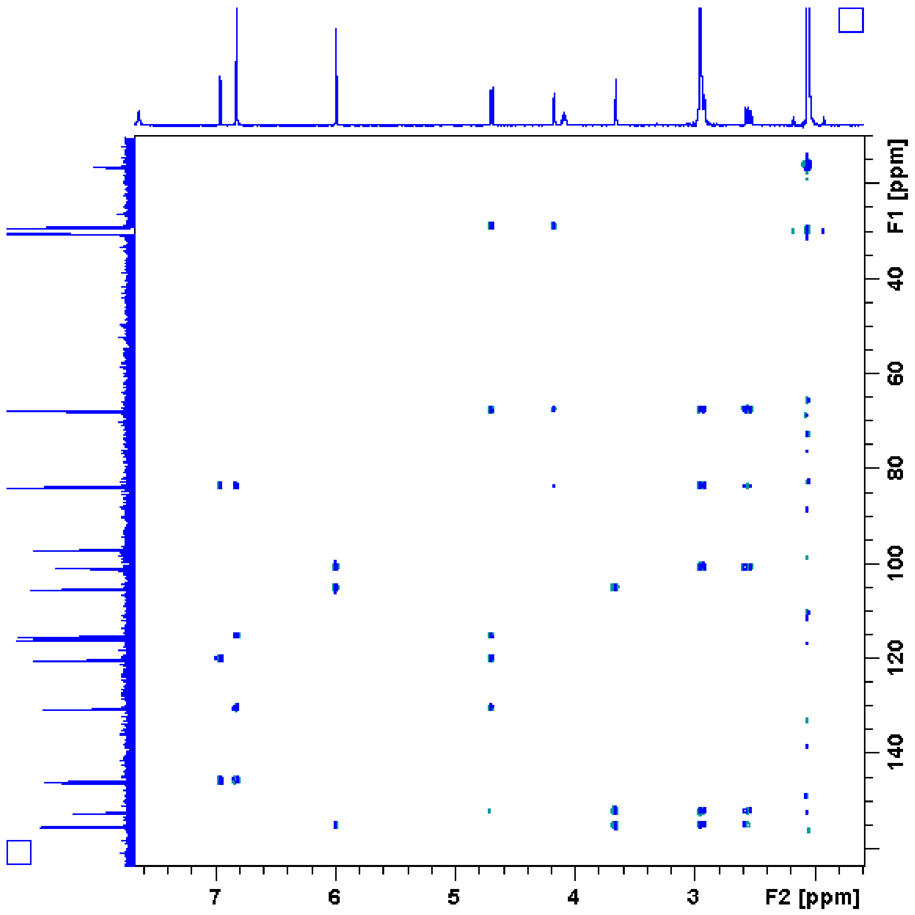

Figure S5. ^{1}H, ^{13}C HMBC spectrum of catechin–methylene–catechin (**1**).

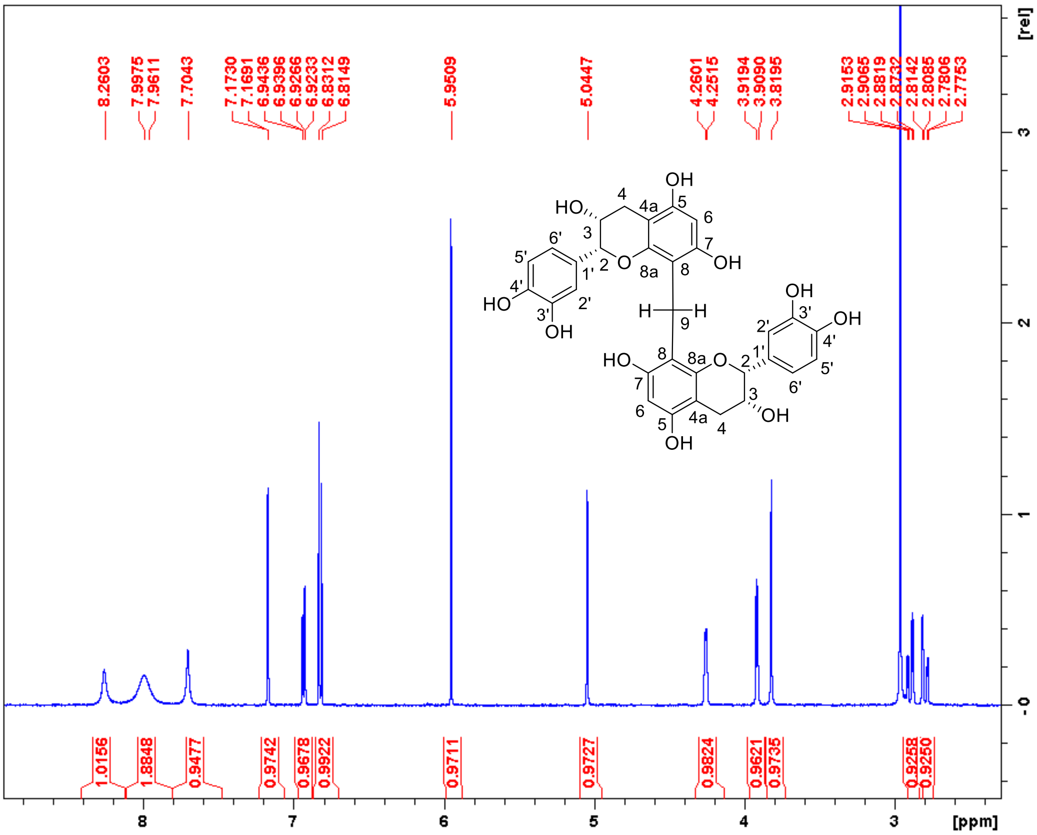

Figure S6. ^{1}H NMR spectrum and structure of epicatechin–methylene–epicatechin (**2**).

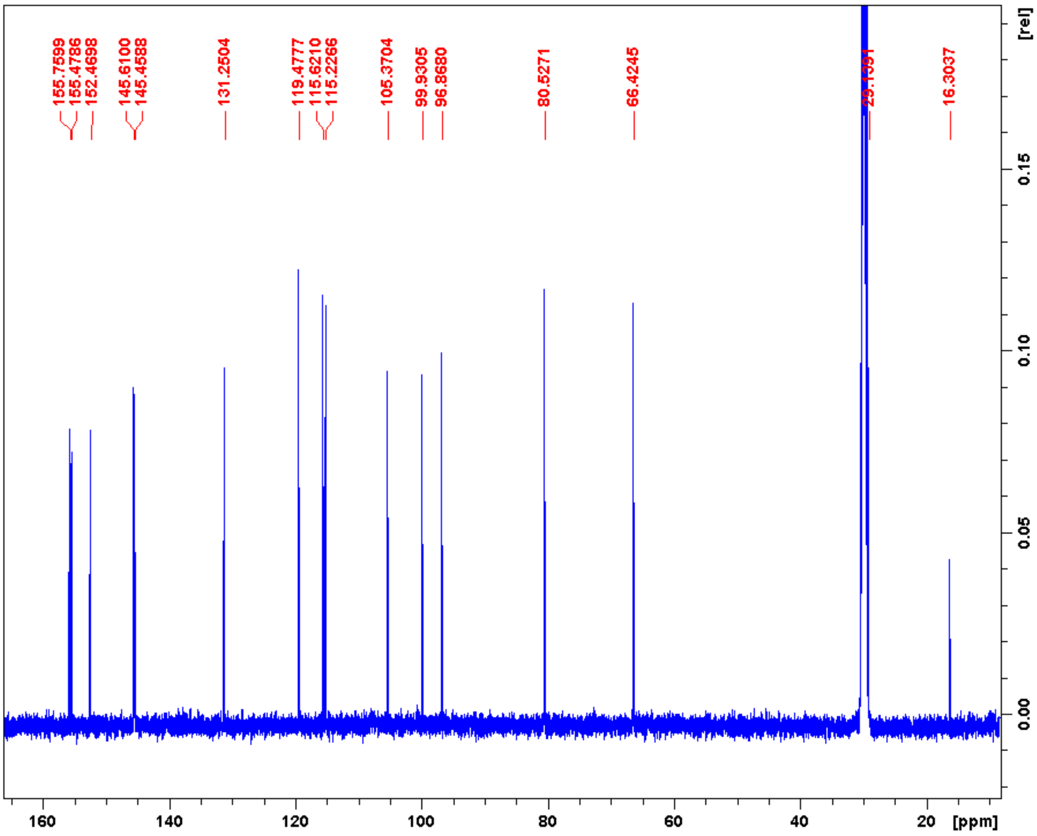

Figure S7. ^{13}C NMR spectrum of epicatechin–methylene–epicatechin (**2**).

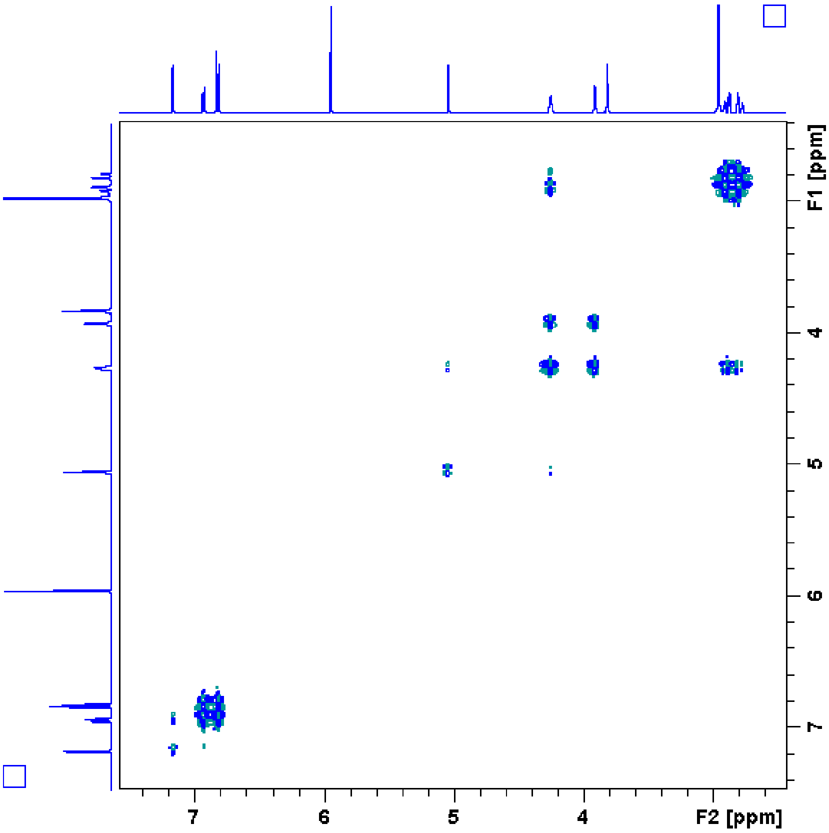

Figure S8. ^{1}H, ^{1}H COSY spectrum of epicatechin–methylene–epicatechin (**2**).

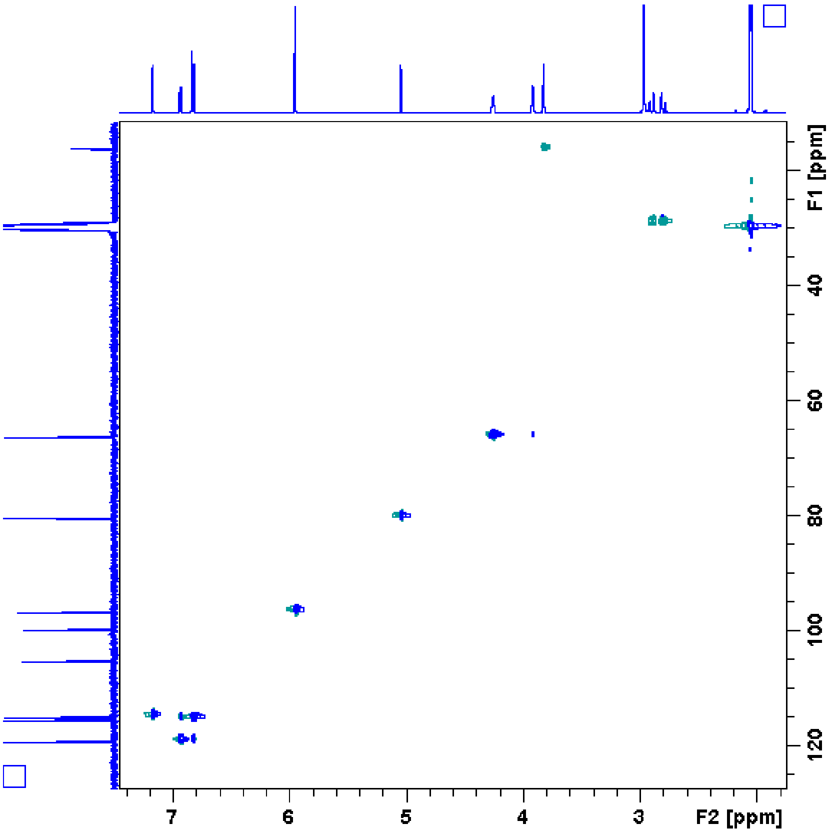

Figure S9. ^{1}H, ^{13}C HSQC spectrum of epicatechin–methylene–epicatechin (**2**).

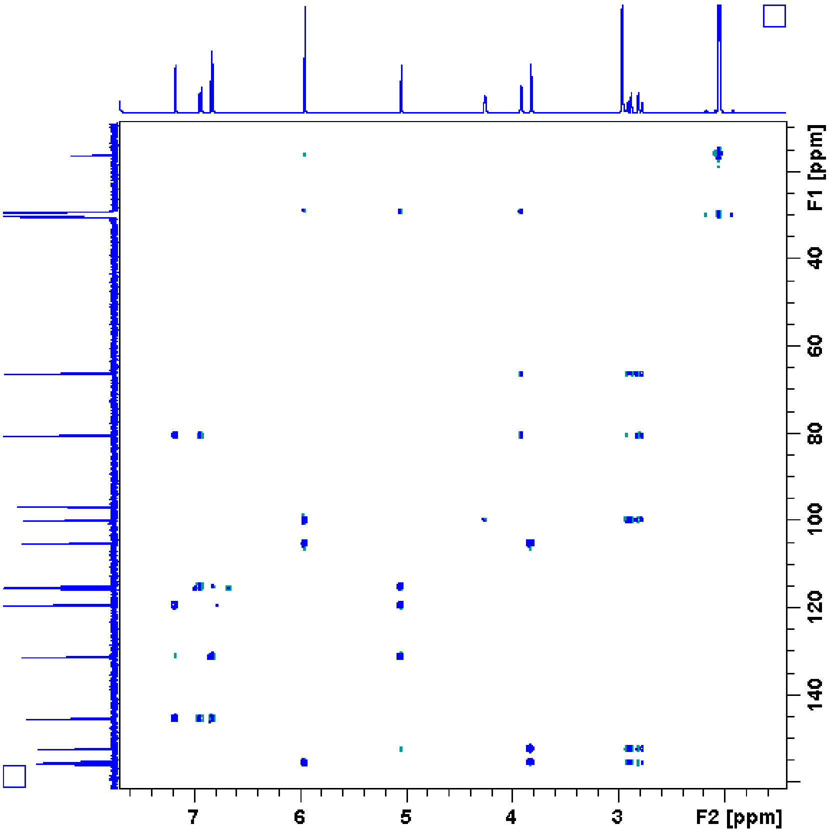

Figure S10. ^{1}H, ^{13}C HMBC spectrum of epicatechin–methylene–epicatechin (**2**).

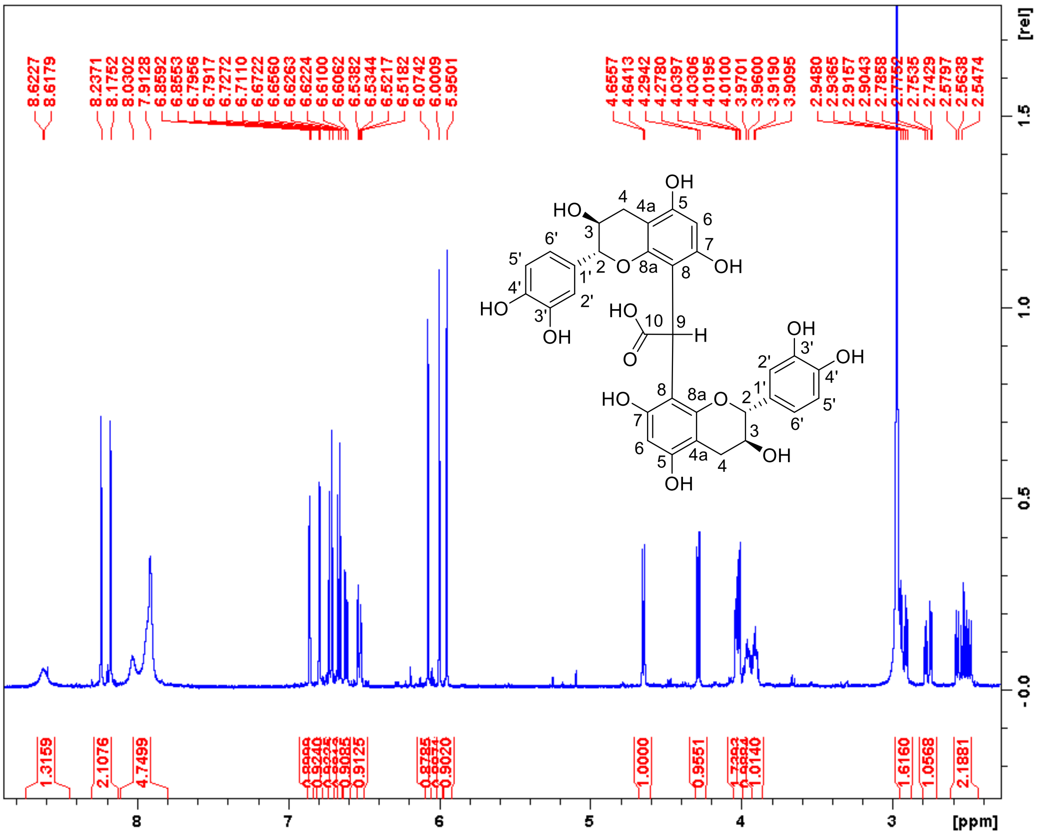

Figure S11. ^{1}H NMR spectrum and structure of catechin–carboxymethine–catechin (**3**)

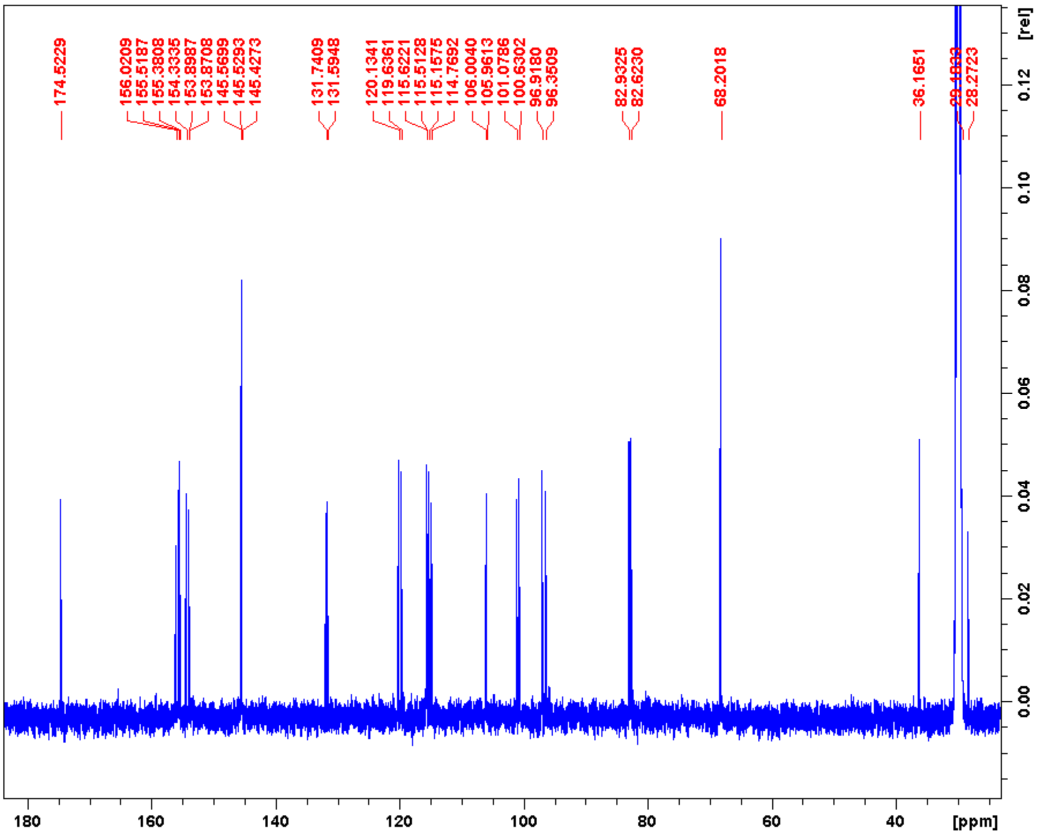

Figure S12. ^{13}C NMR spectrum of catechin–carboxymethine–catechin (**3**)

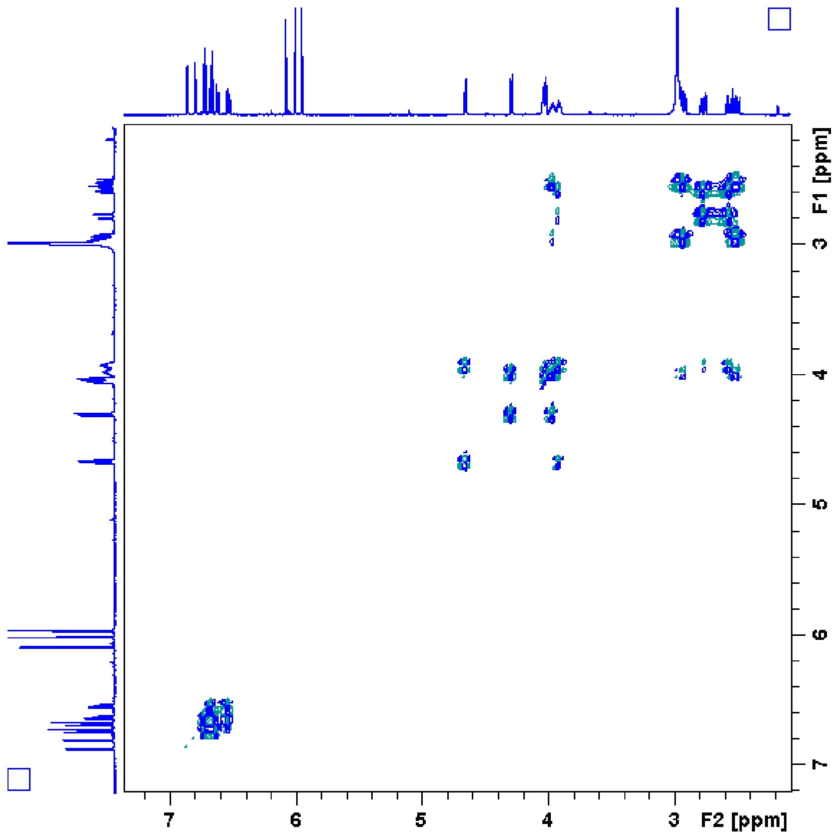

Figure S13. ^{1}H, ^{1}H COSY spectrum of catechin–carboxymethine–catechin (**3**)

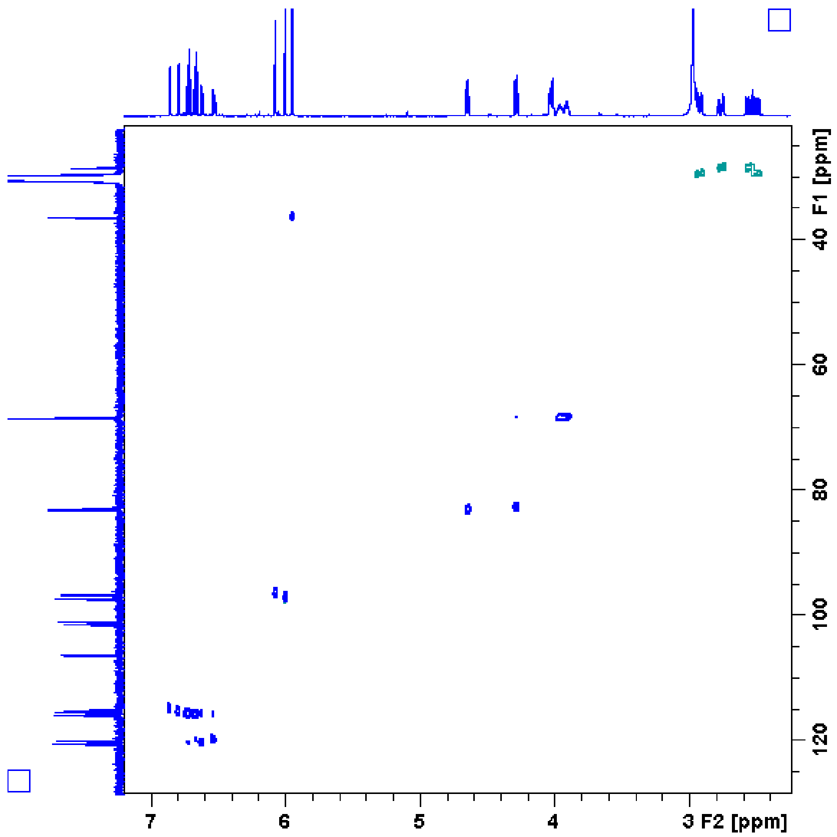

Figure S14. ^{1}H, ^{13}C HSQC spectrum of catechin–carboxymethine–catechin (**3**)

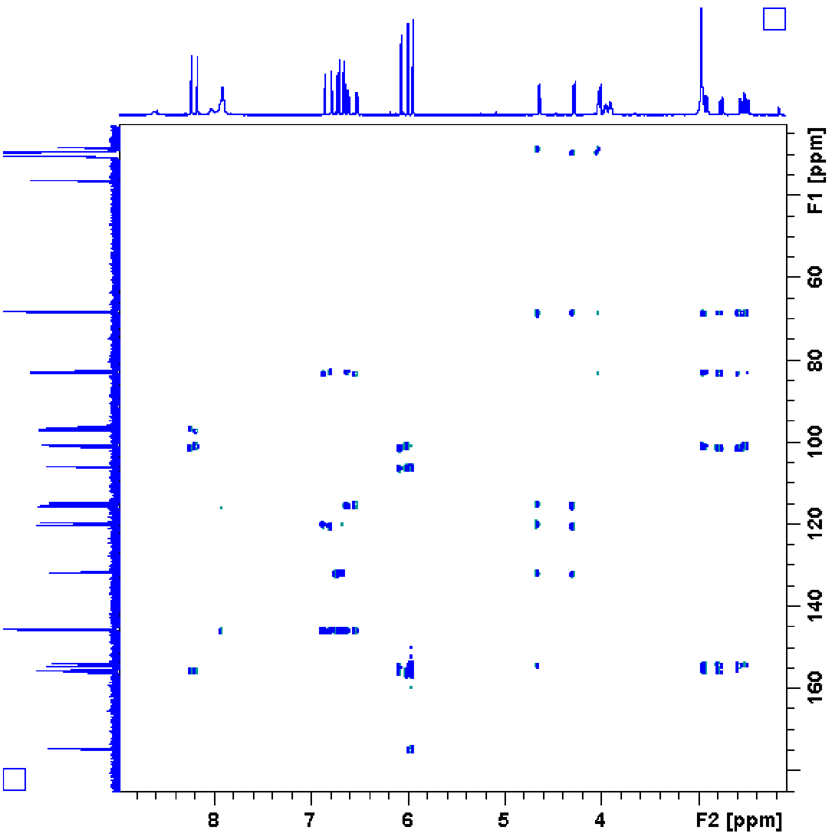

Figure S15. ^{1}H, ^{13}C HMBC spectrum of catechin–carboxymethine–catechin (**3**)

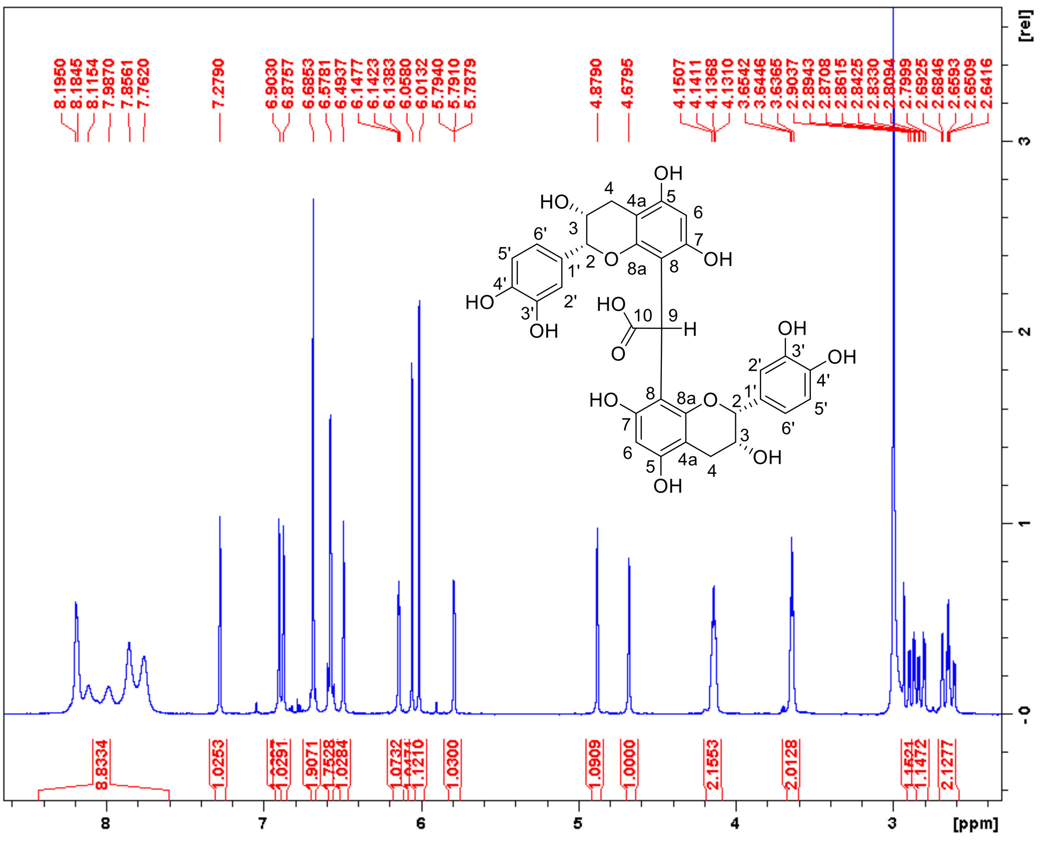

Figure S16. ^{1}H NMR spectrum and structure of epicatechin–carboxymethine–epicatechin (**4**)

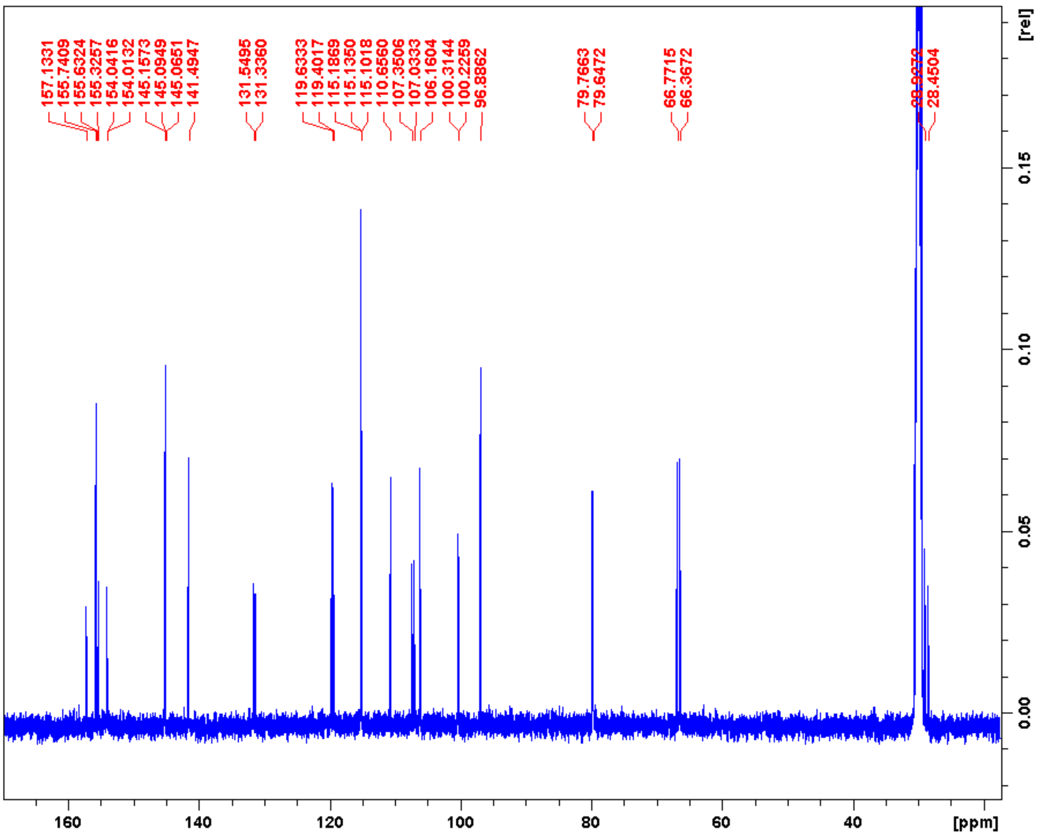

Figure S17. ^{13}C NMR spectrum of epicatechin–carboxymethine–epicatechin (**4**)

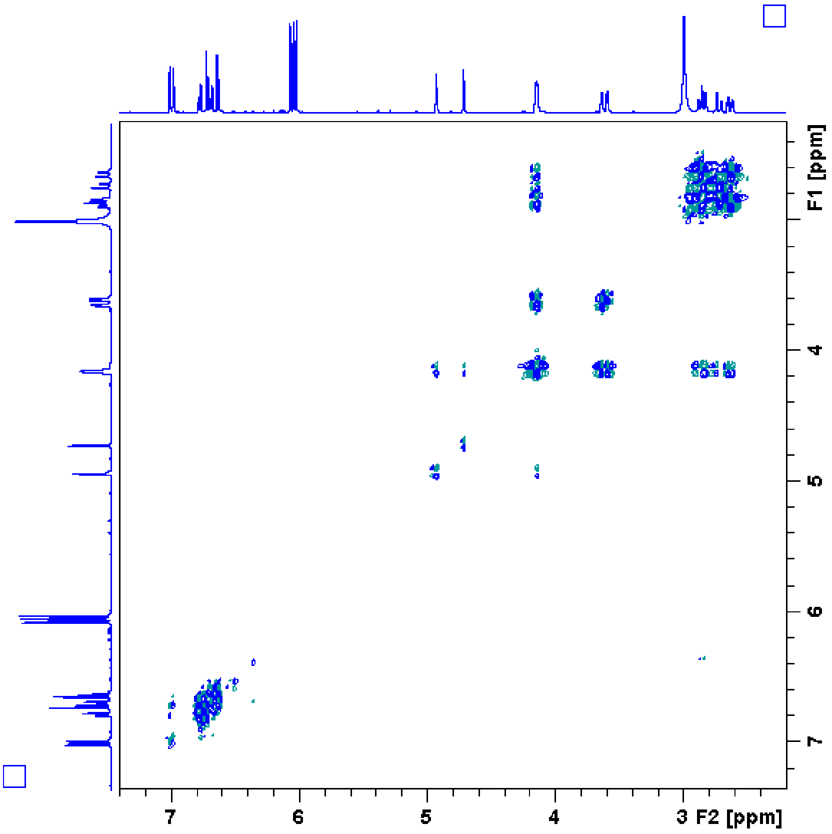

Figure S18. ^{1}H, ^{1}H COSY spectrum of epicatechin–carboxymethine–epicatechin (**4**)

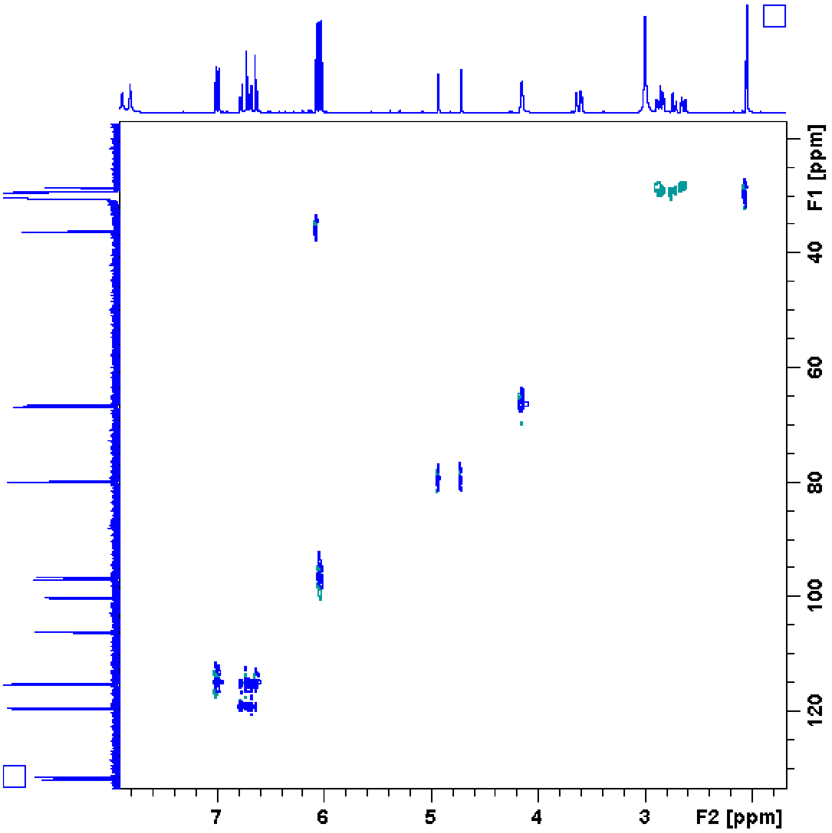

Figure S19. ^{1}H, ^{13}C HSQC spectrum of epicatechin–carboxymethine–epicatechin (**4**)

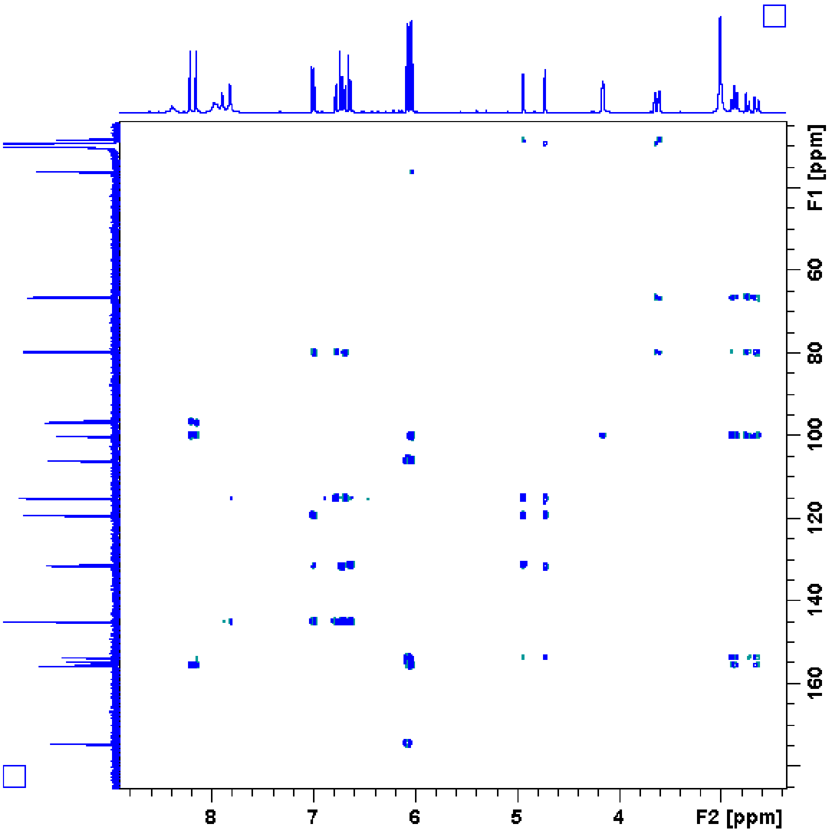

Figure S20. ^{1}H, ^{13}C HMBC spectrum of epicatechin–carboxymethine–epicatechin (**4**)

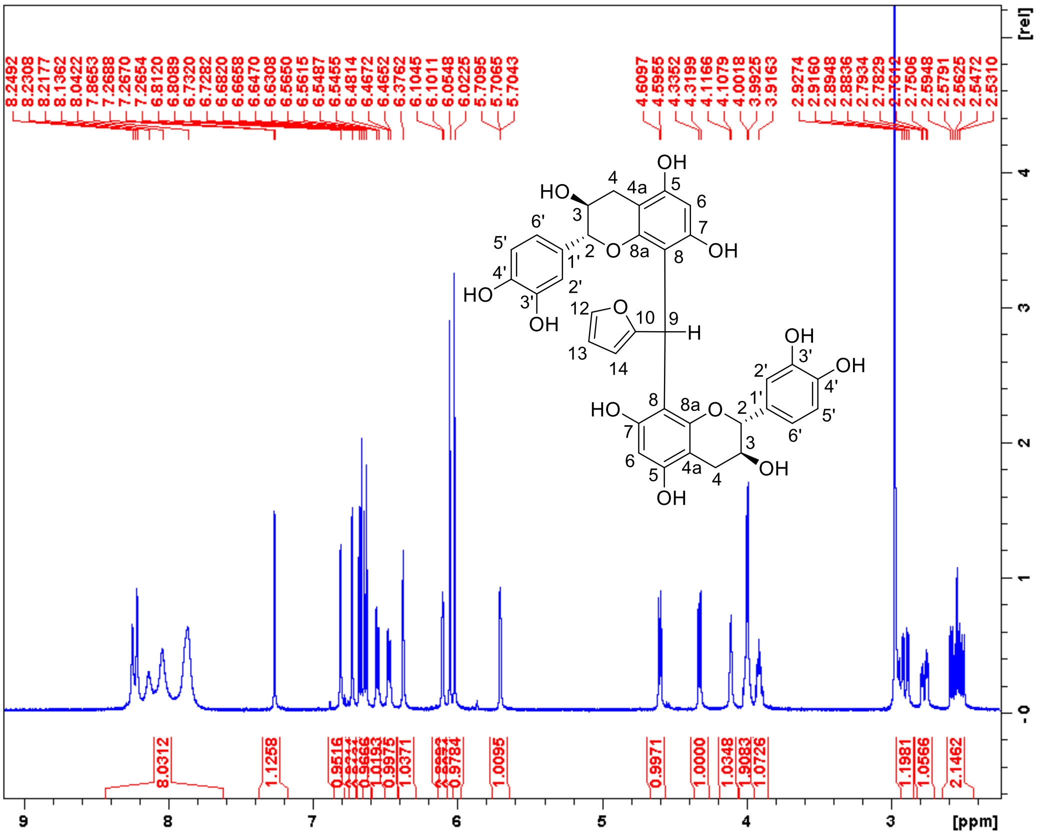

Figure S21. ^{1}H NMR spectrum and structure of catechin–furanmethine–catechin (**5**)

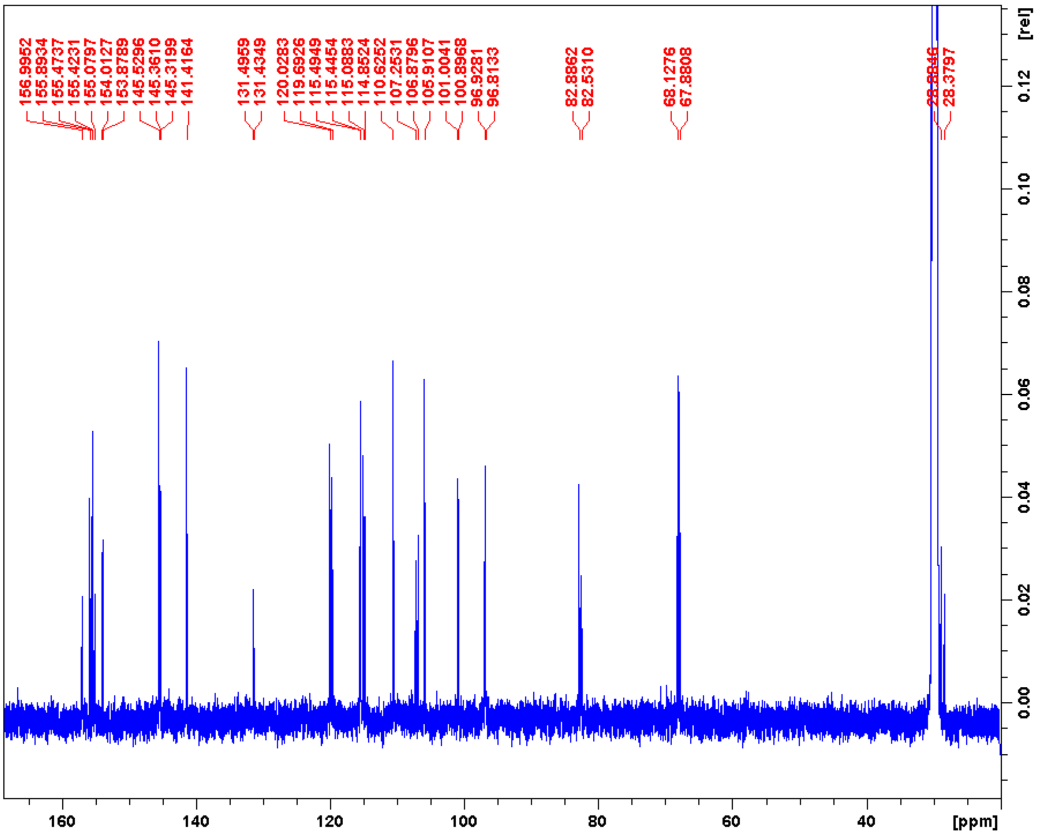

Figure S22. ^{13}C NMR spectrum of catechin–furanmethine–catechin (**5**)

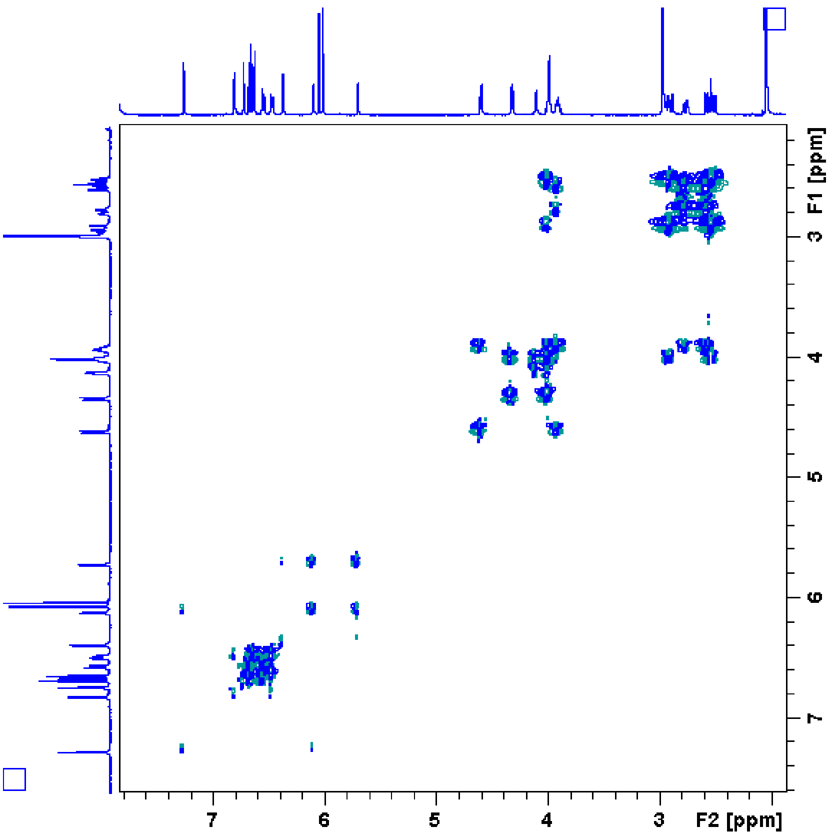

Figure S23. ^{1}H, ^{1}H COSY spectrum of catechin–furanmethine–catechin (**5**)

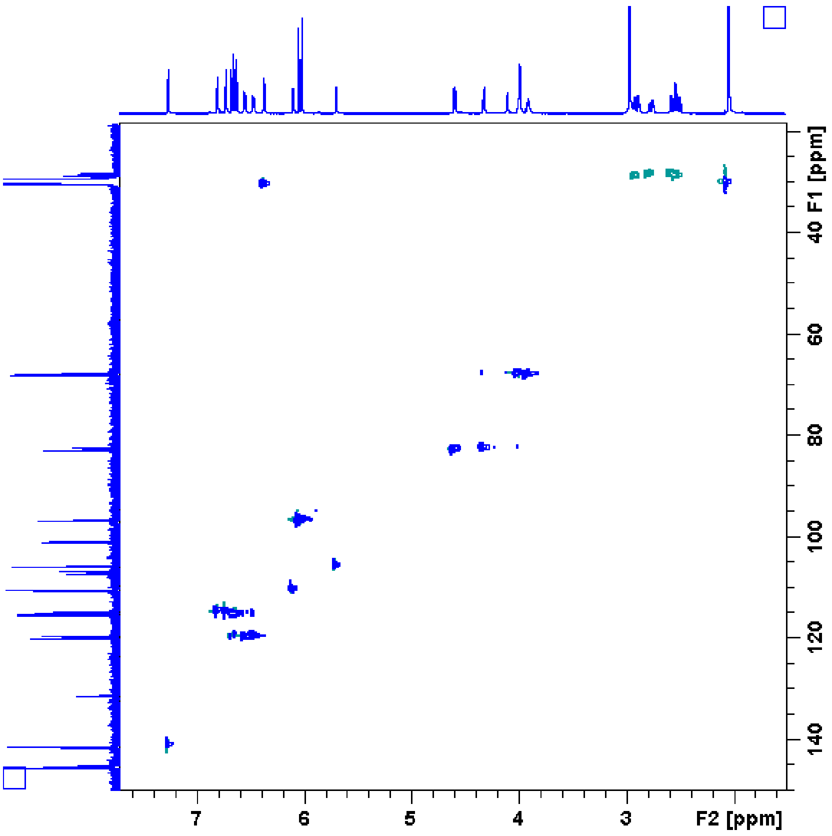

Figure S24. ^{1}H, ^{13}C HSQC spectrum of catechin–furanmethine–catechin (**5**)

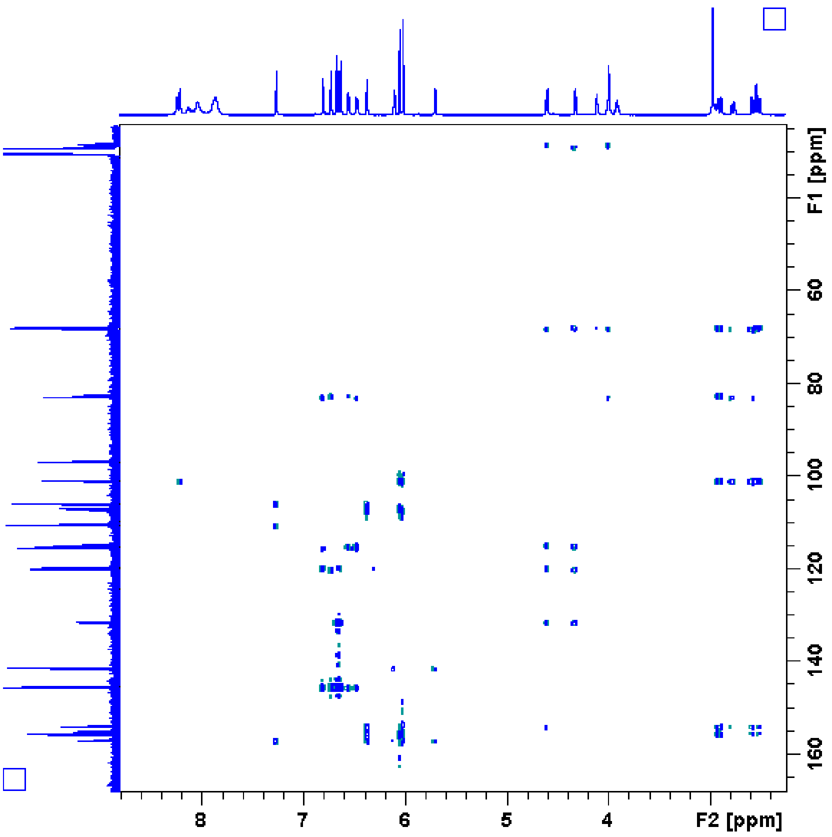

Figure S25. ^{1}H, ^{13}C HMBC spectrum of catechin–furanmethine–catechin (**5**)

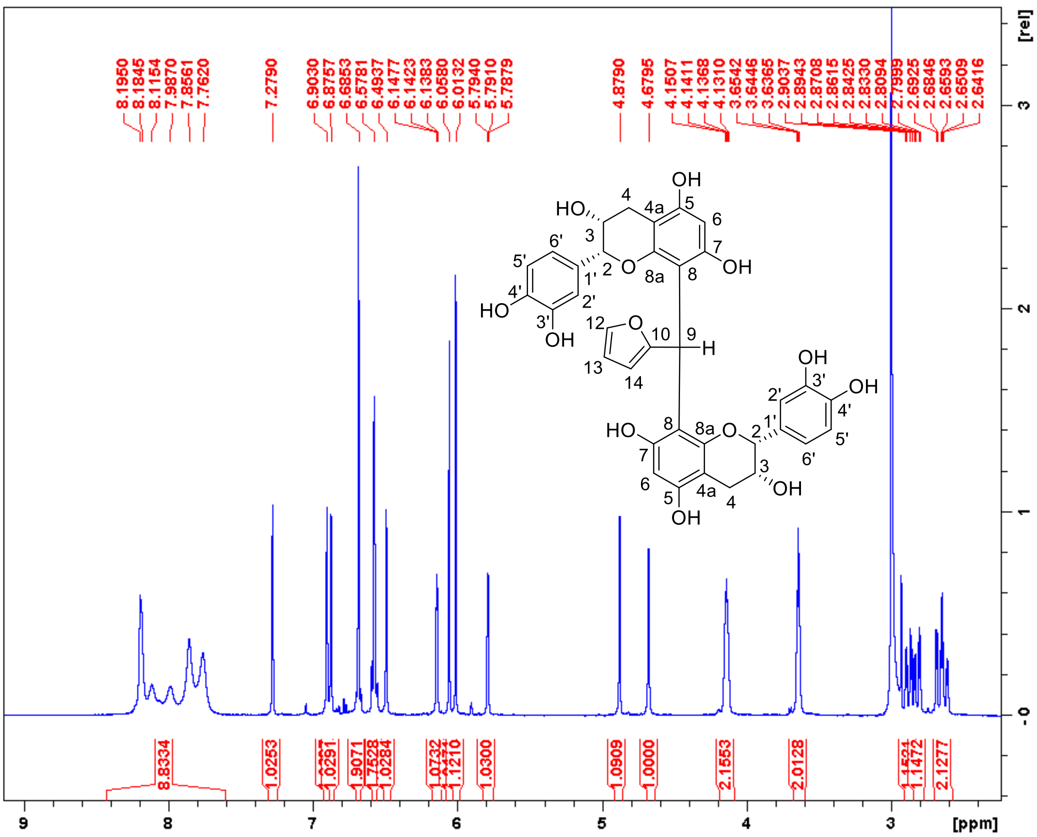

Figure S26. ¹H NMR spectrum and structure of epicatechin–furanmethine–epicatechin (**6**)

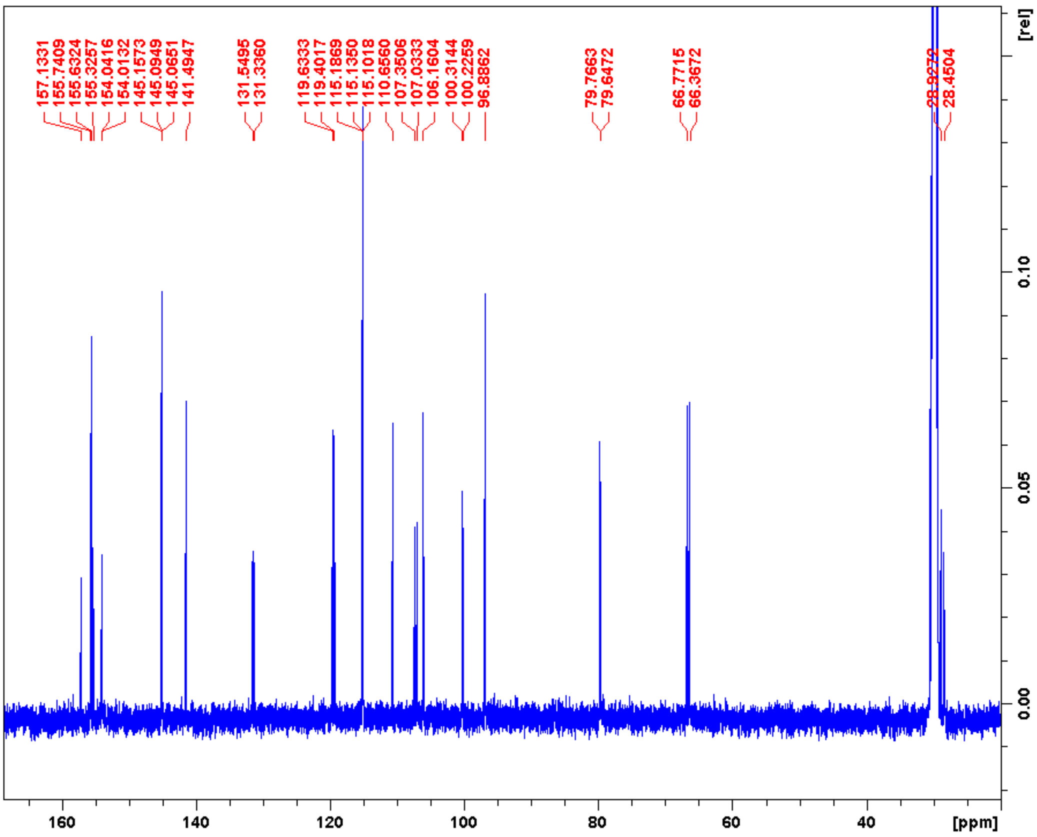

Figure S27. ¹³C NMR spectrum of epicatechin–furanmethine–epicatechin (**6**)

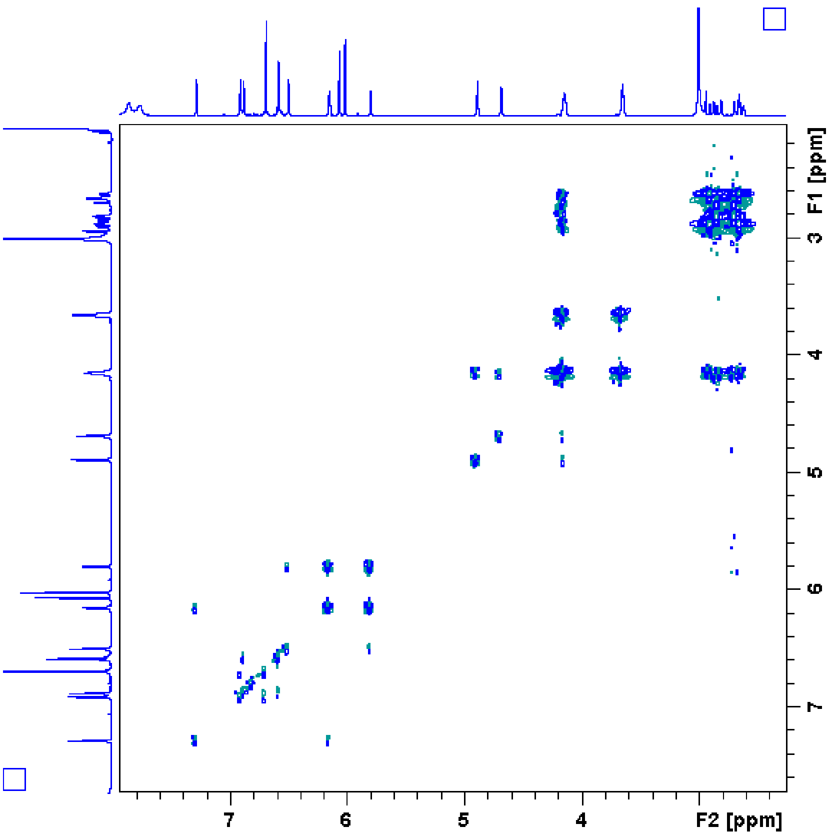

Figure S28. ^{1}H, ^{1}H COSY spectrum of epicatechin–furanmethine–epicatechin (**6**)

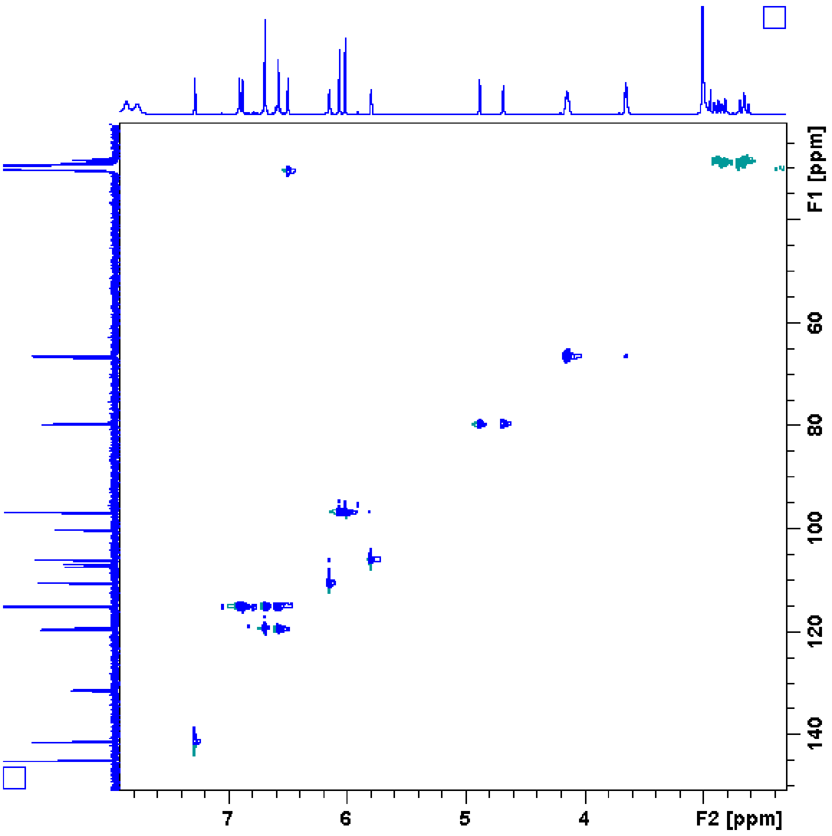

Figure S29. ^{1}H, ^{13}C HSQC spectrum of epicatechin–furanmethine–epicatechin (**6**)

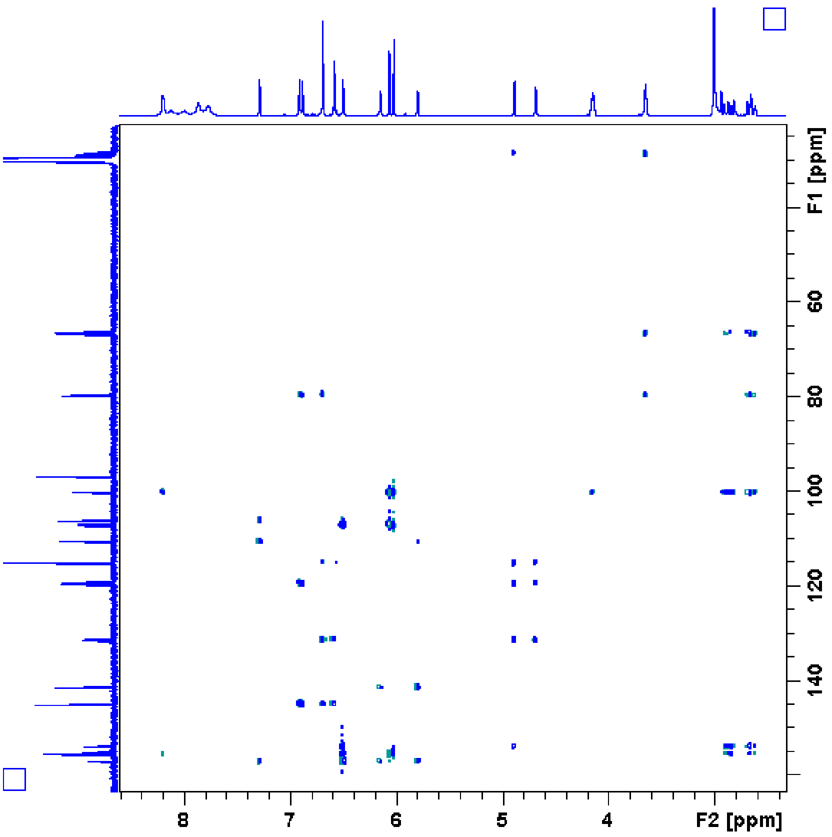

Figure S30. ^{1}H, ^{13}C HMBC spectrum of epicatechin–furanmethine–epicatechin (**6**)

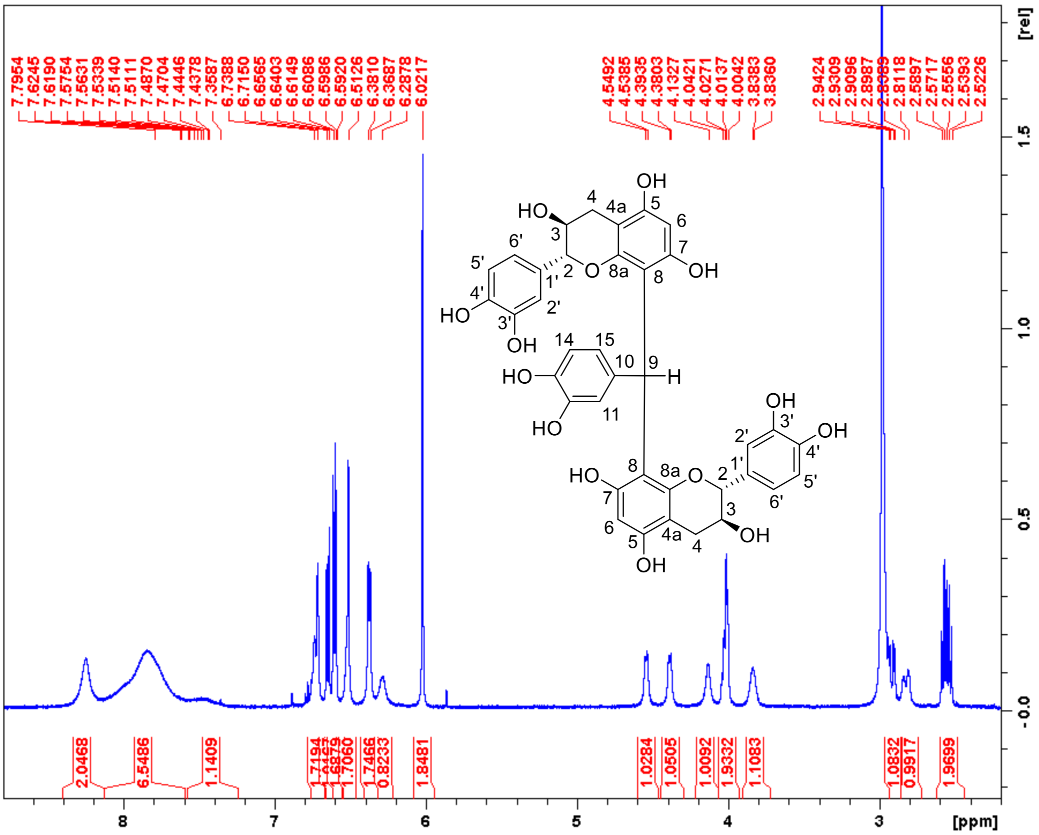

Figure S31. ^{1}H NMR spectrum and structure of catechin–catecholmethine–catechin (**7**)

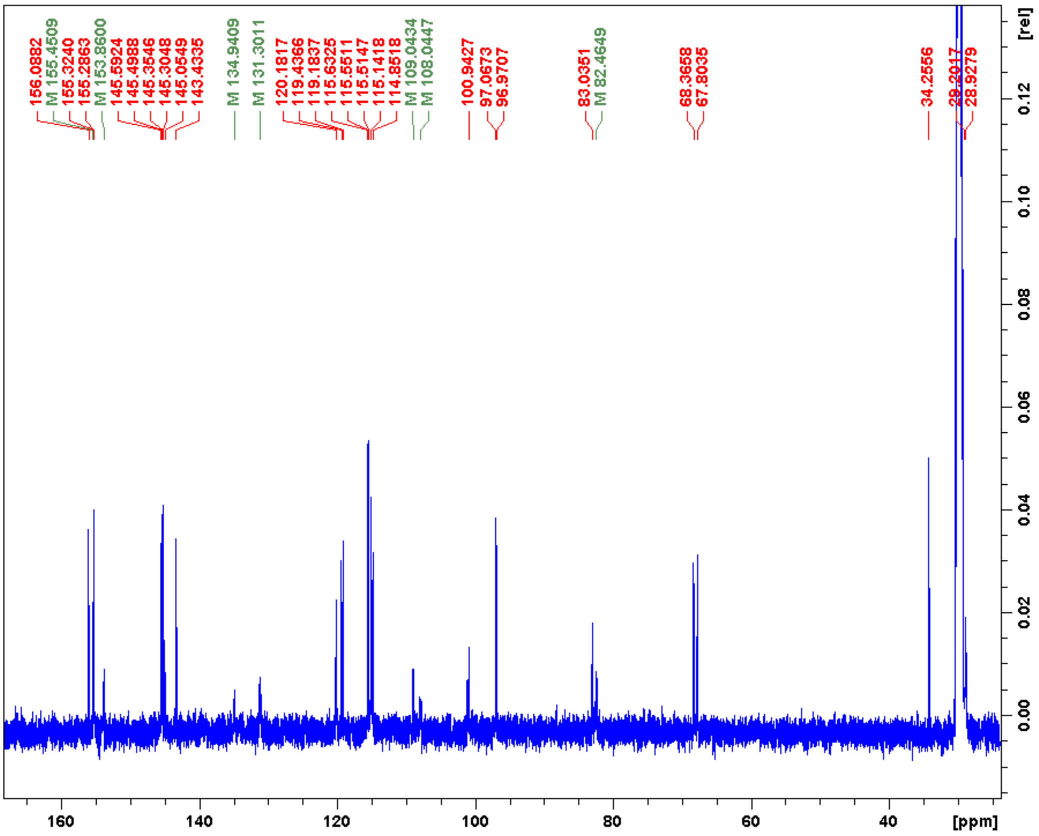

Figure S32. ^{13}C NMR spectrum of catechin–catecholmethine–catechin (**7**)

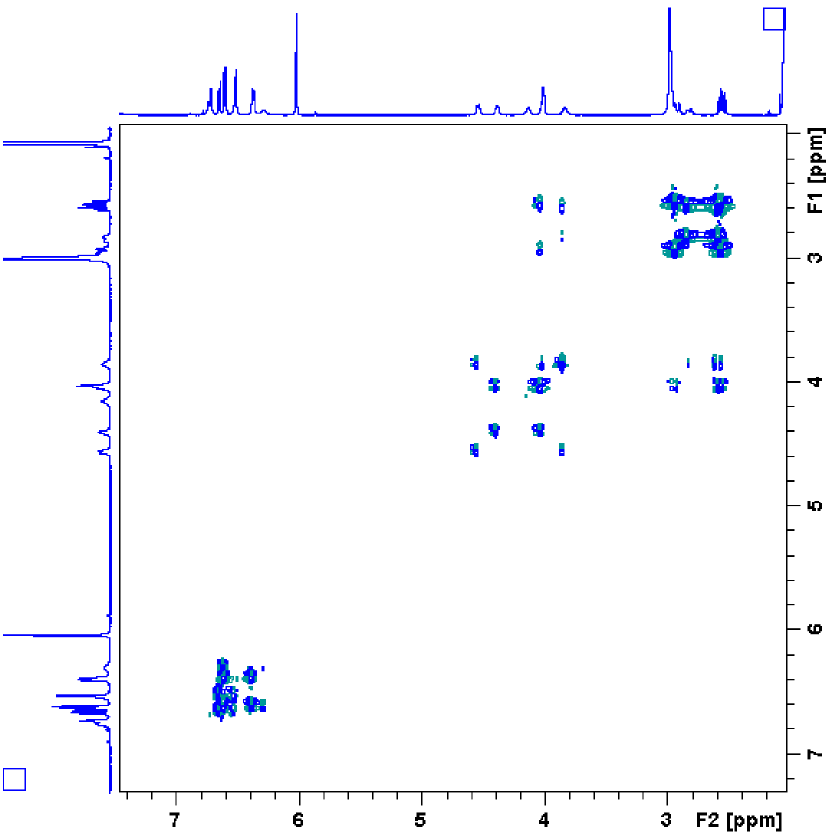

Figure S33. ^{1}H, ^{1}H COSY spectrum of catechin–catecholmethine–catechin (**7**)

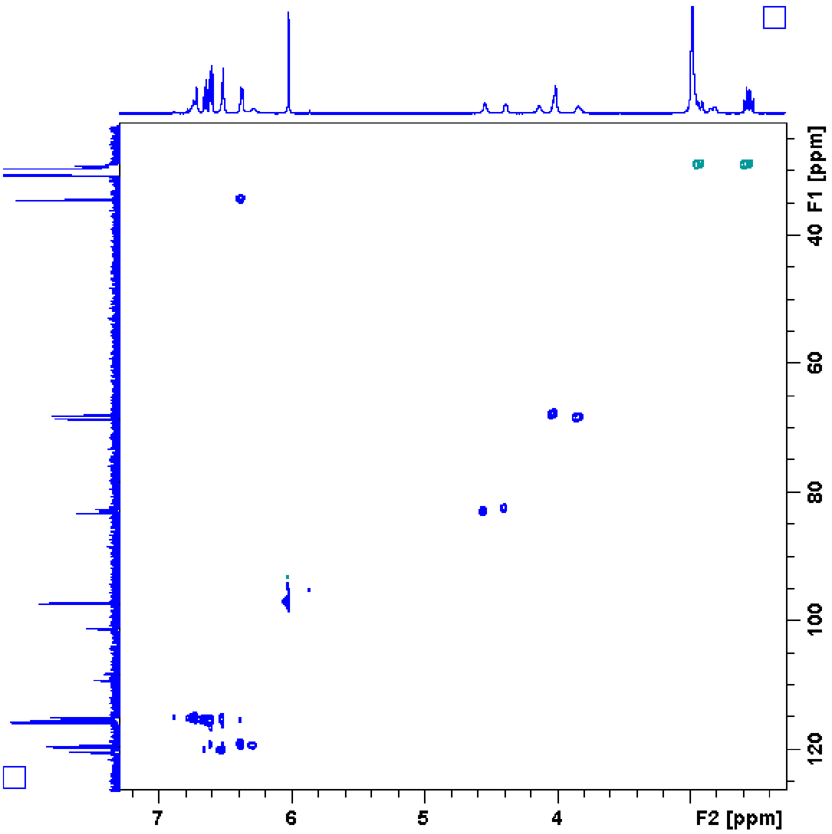

Figure S34. ^{1}H, ^{13}C HSQC spectrum of catechin–catecholmethine–catechin (**7**)

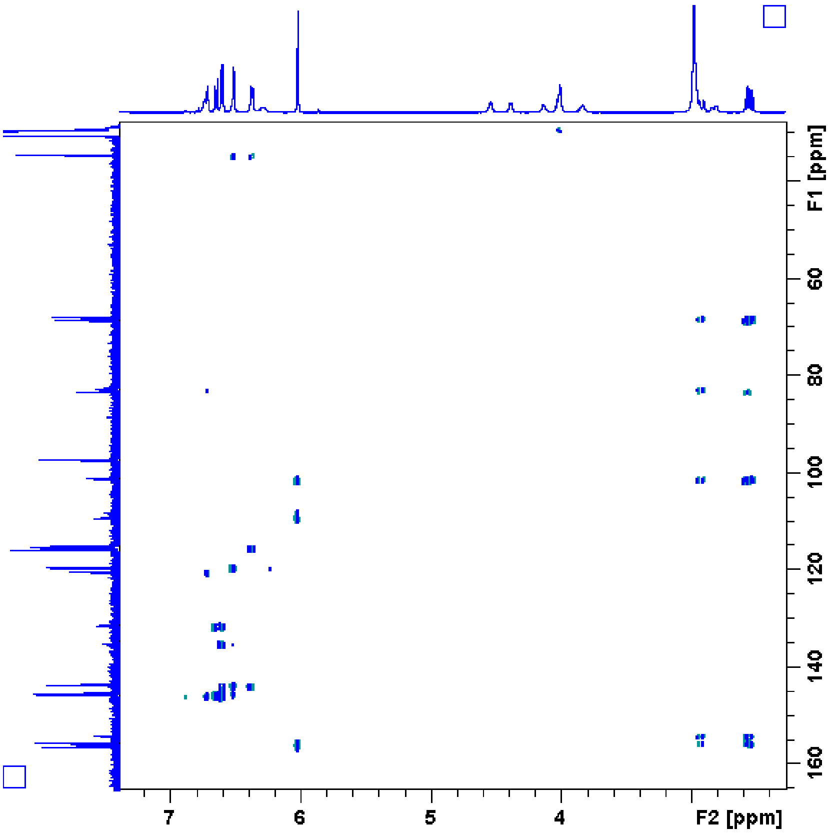

Figure S35. ^{1}H, ^{13}C HMBC spectrum of catechin–catecholmethine–catechin (**7**)

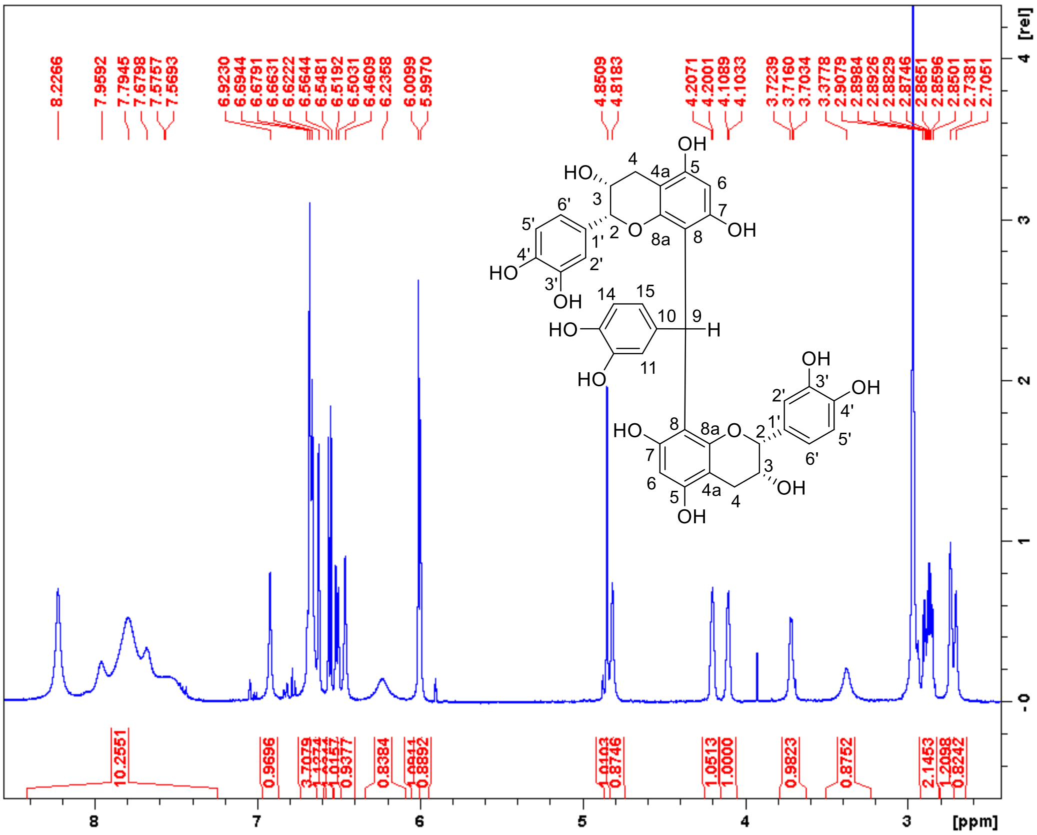

Figure S36. ^{1}H NMR spectrum and structure of epicatechin–catecholmethine–epicatechin (**8**)

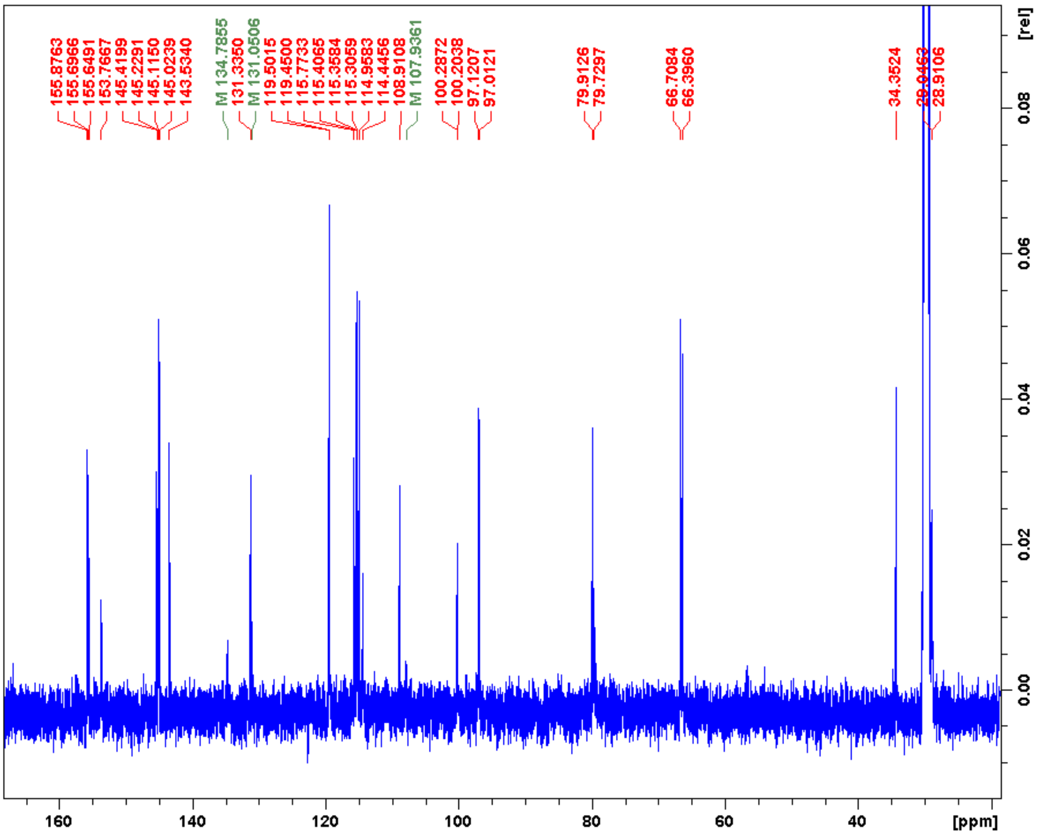

Figure S37. ^{13}C NMR spectrum of epicatechin–catecholmethine–epicatechin (**8**)

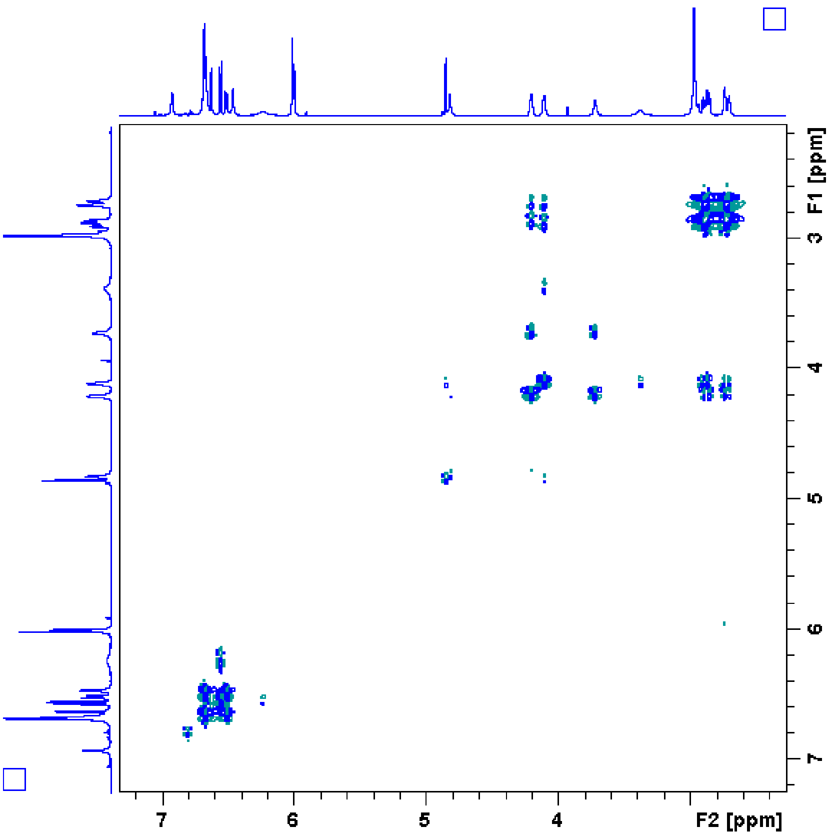

Figure S38. ¹H, ¹H COSY spectrum of epicatechin–catecholmethine–epicatechin (**8**)

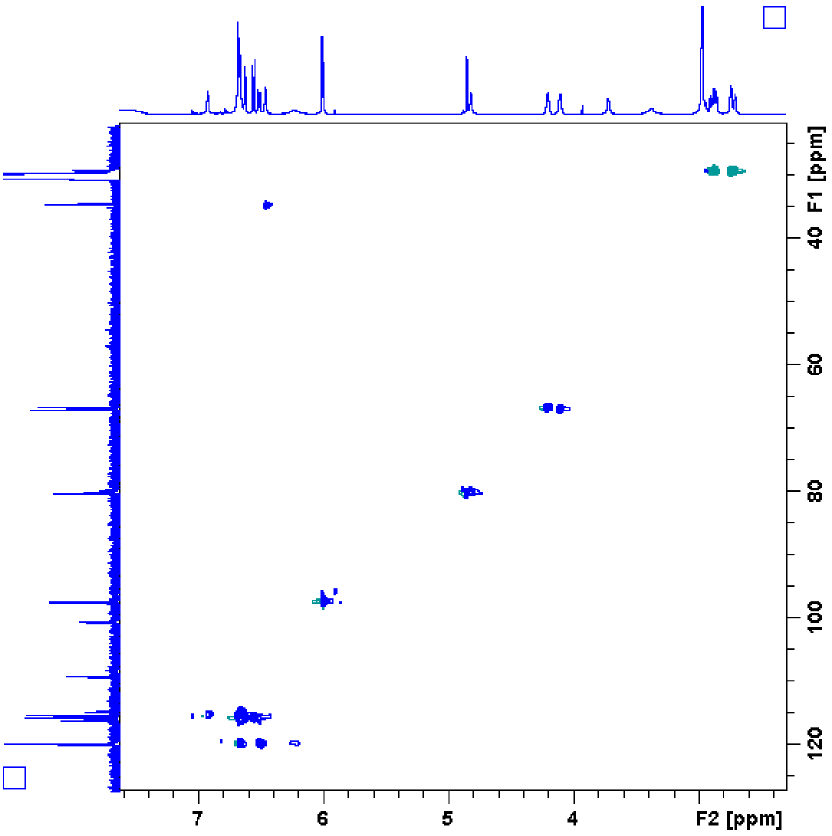

Figure S39. ¹H, ¹³C HSQC spectrum of epicatechin–catecholmethine–epicatechin (**8**)

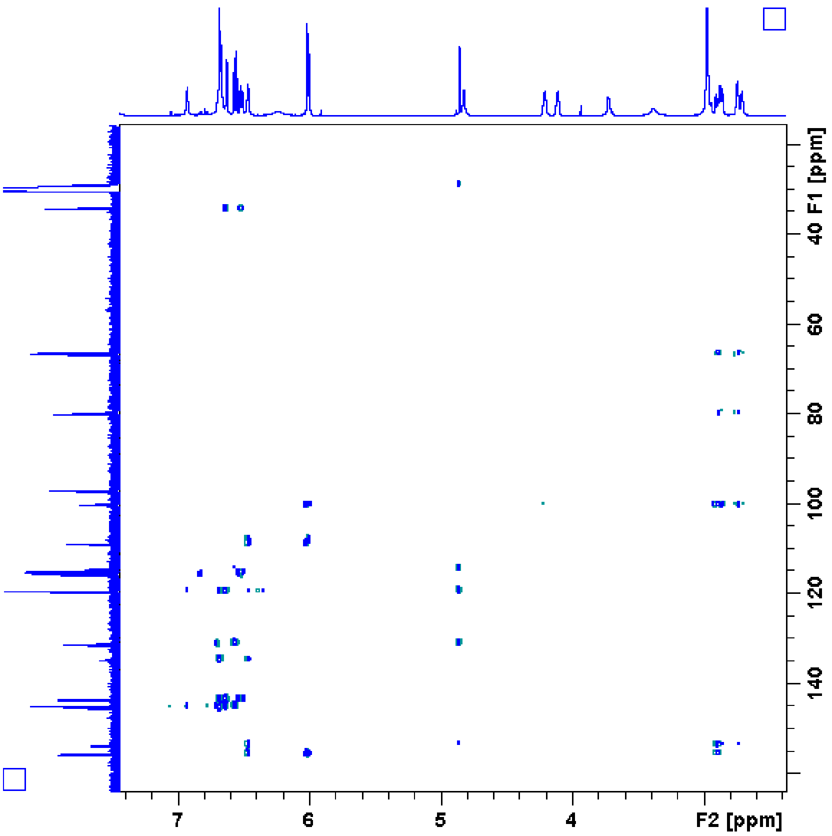

Figure S40. ^{1}H, ^{13}C HMBC spectrum of epicatechin–catecholmethine–epicatechin (**8**)

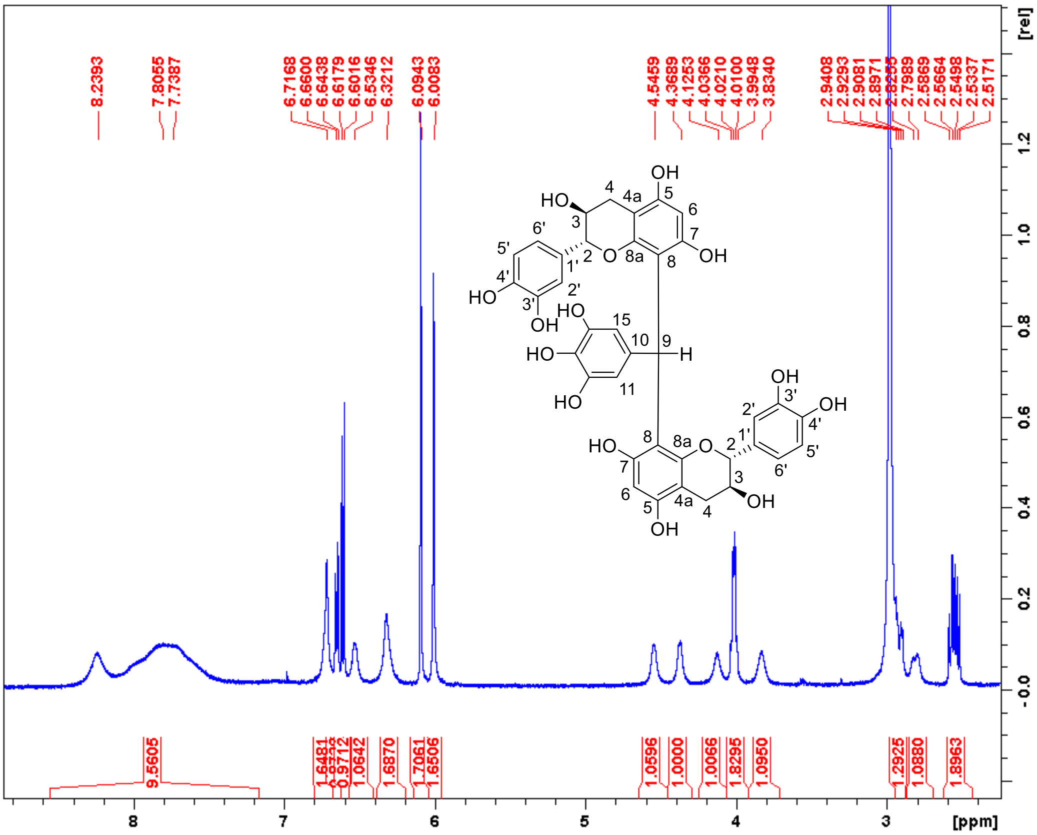

Figure S41. ^{1}H NMR spectrum and structure of catechin–pyrogallolmethine–catechin (**9**)

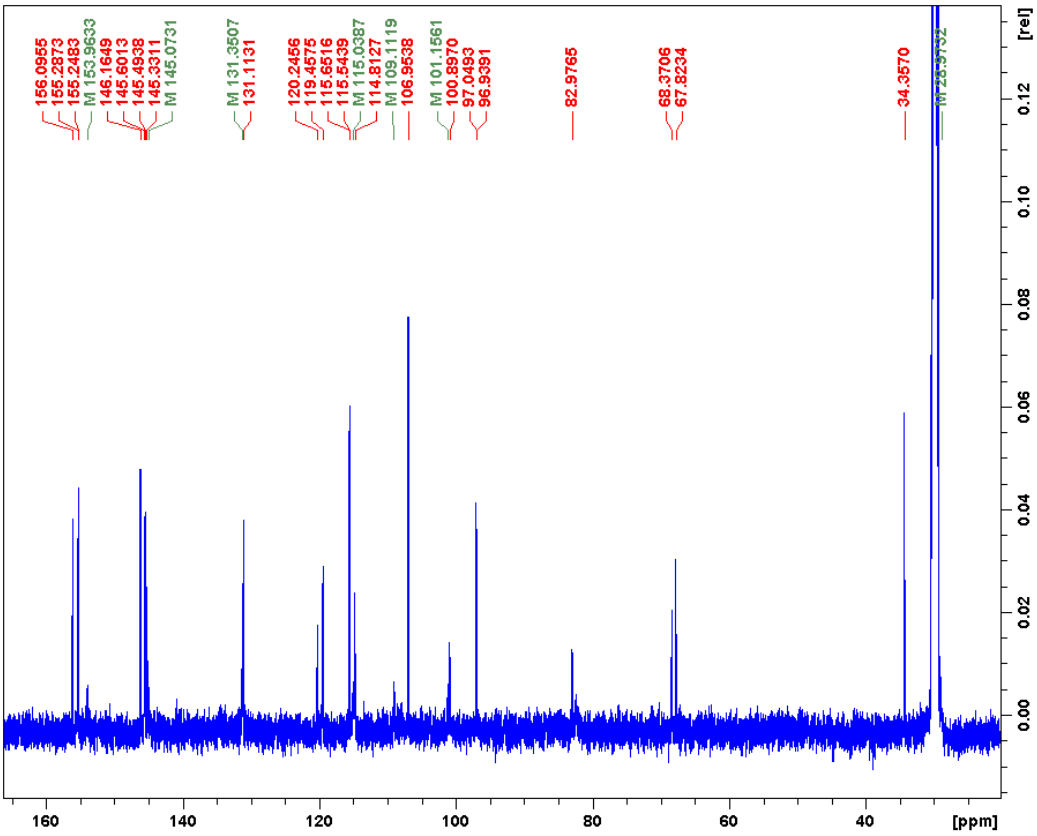

Figure S42. ^{13}C NMR spectrum of catechin–pyrogallolmethine–catechin (**9**)

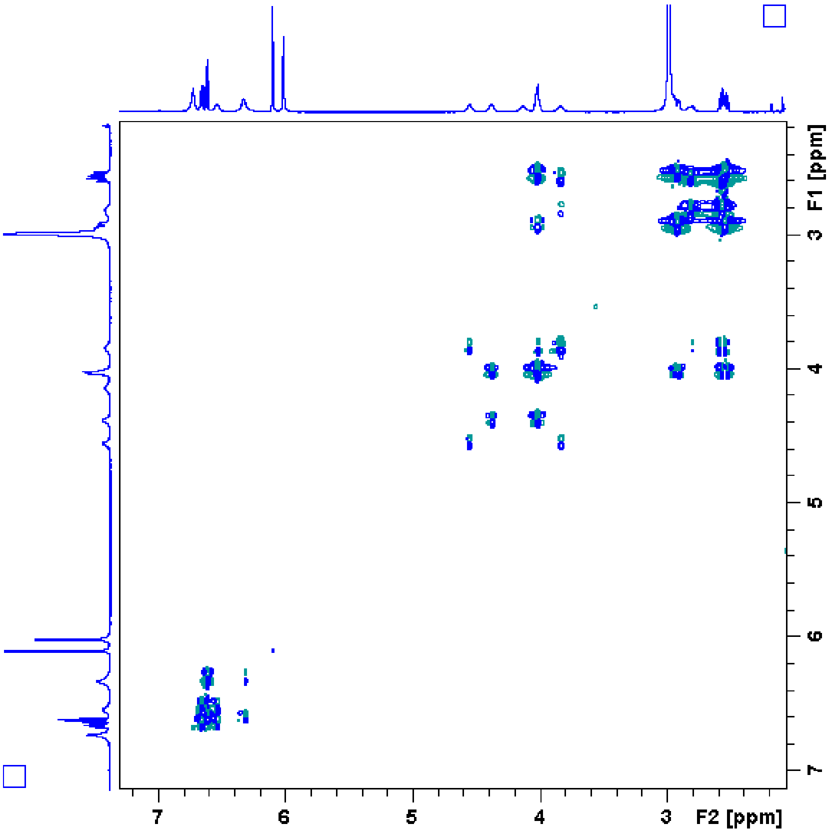

Figure S43. ^{1}H, ^{1}H COSY spectrum of catechin–pyrogallolmethine–catechin (**9**)

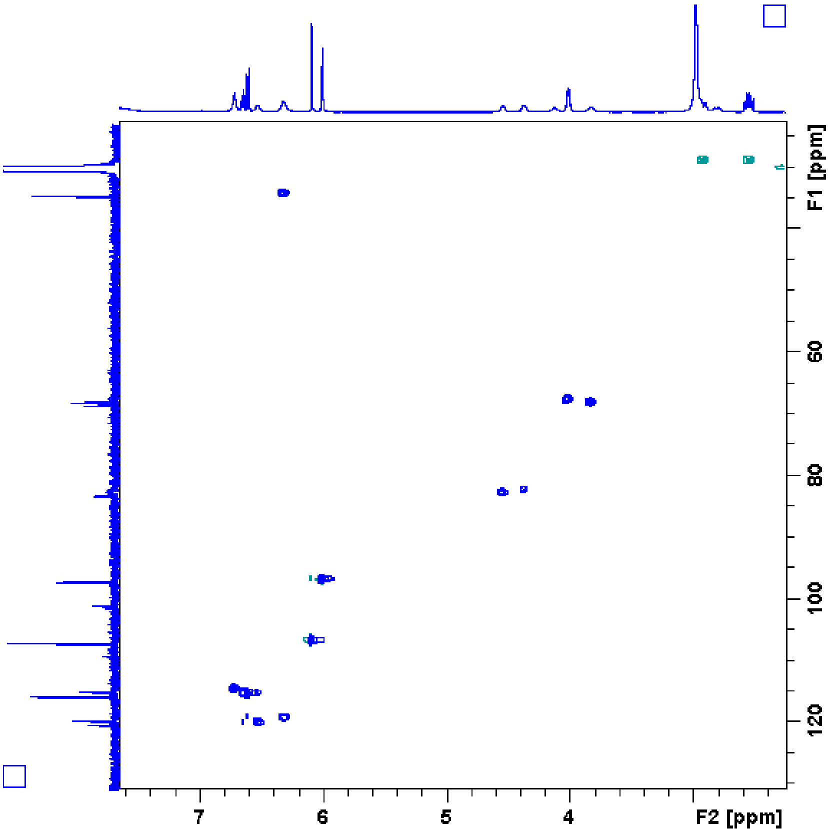

Figure S44. ^{1}H, ^{13}C HSQC spectrum of catechin–pyrogallolmethine–catechin (**9**)

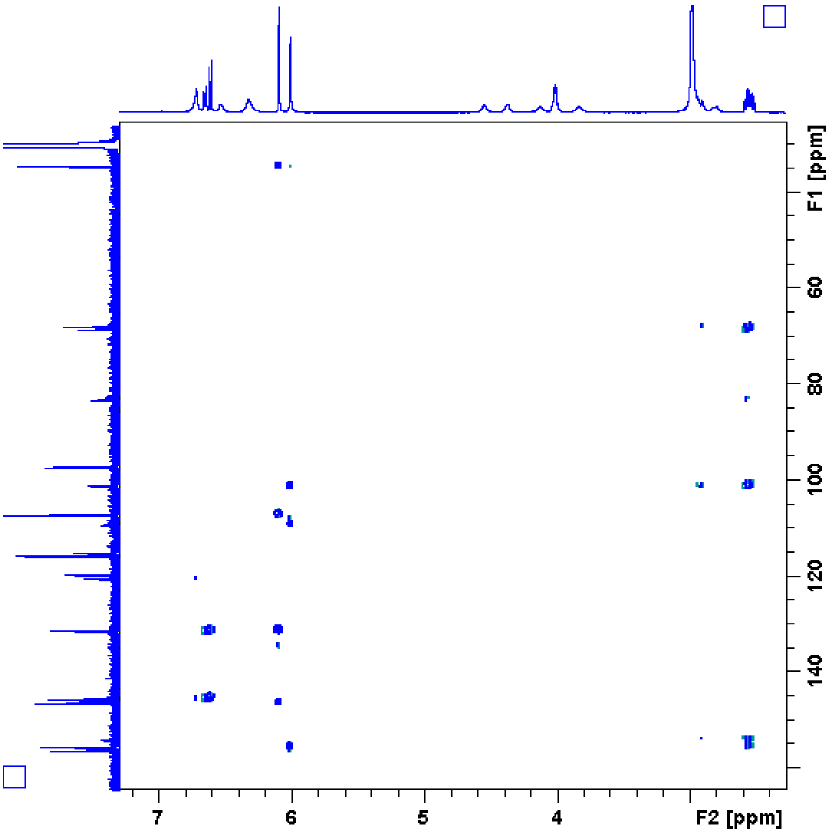

Figure S45. ^{1}H, ^{13}C HMBC spectrum of catechin–pyrogallolmethine–catechin (**9**)

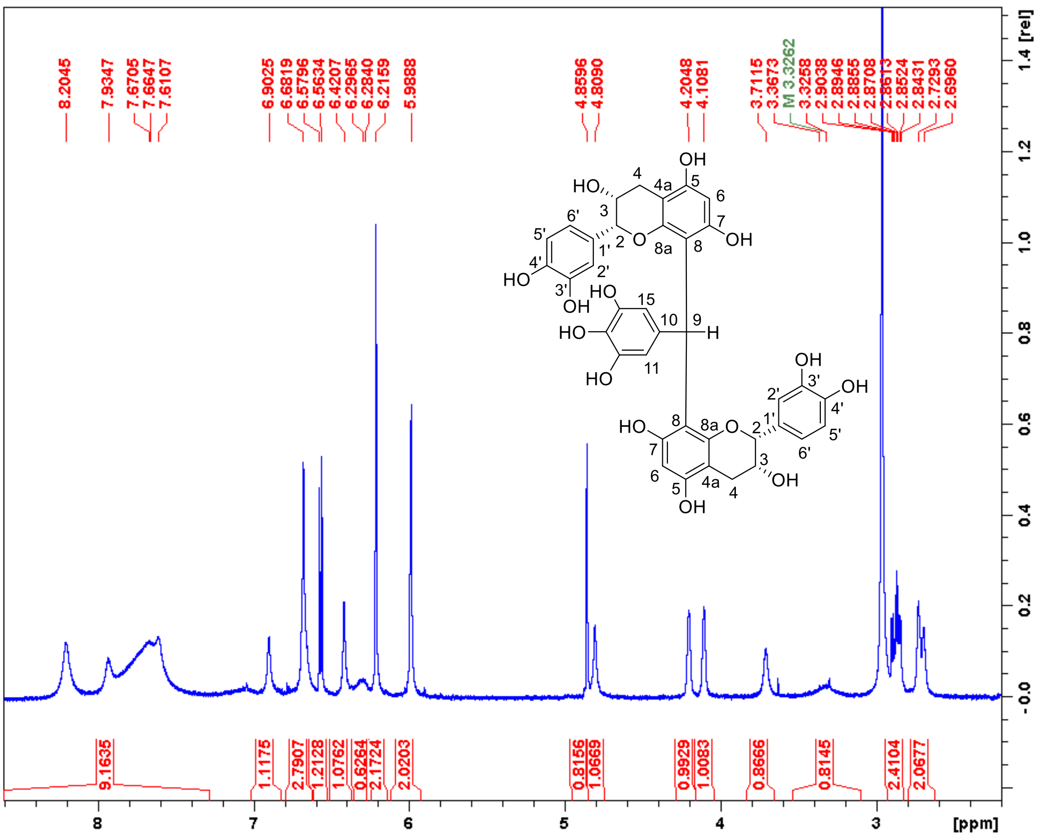

Figure S46. ^{1}H NMR spectrum and structure of epicatechin–pyrogallolmethine–epicatechin (**10**)

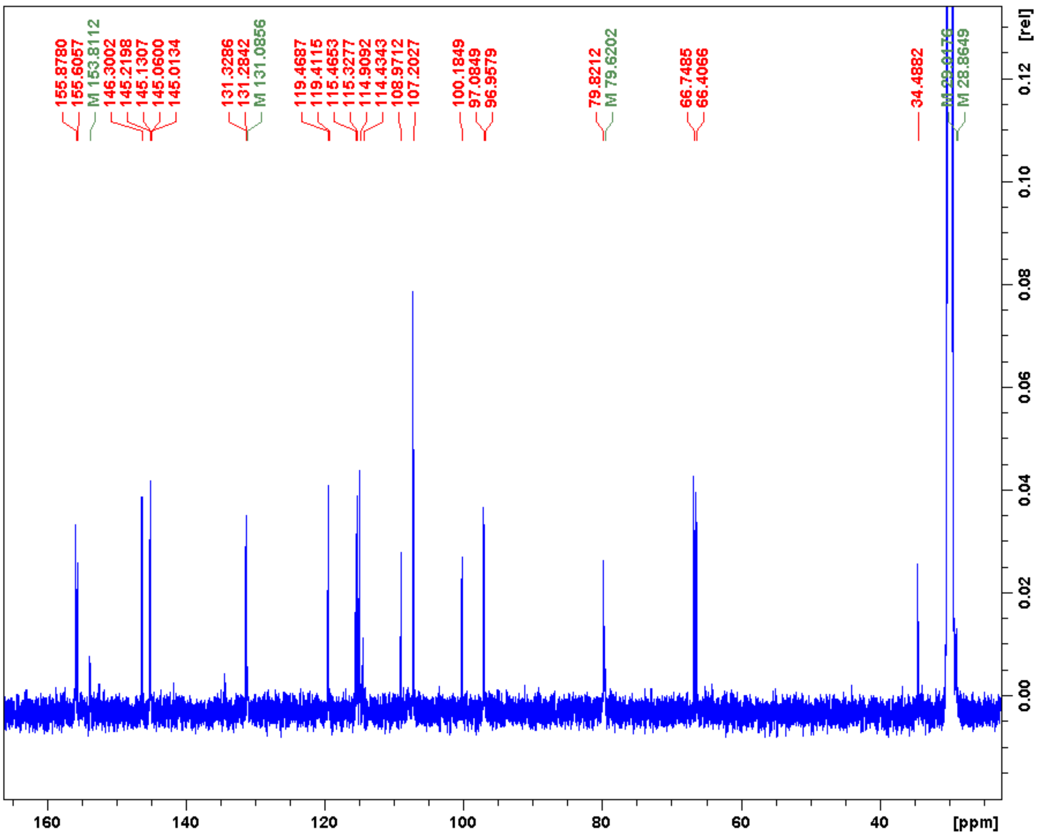

Figure S47. ^{13}C NMR spectrum of epicatechin–pyrogallolmethine–epicatechin (**10**)

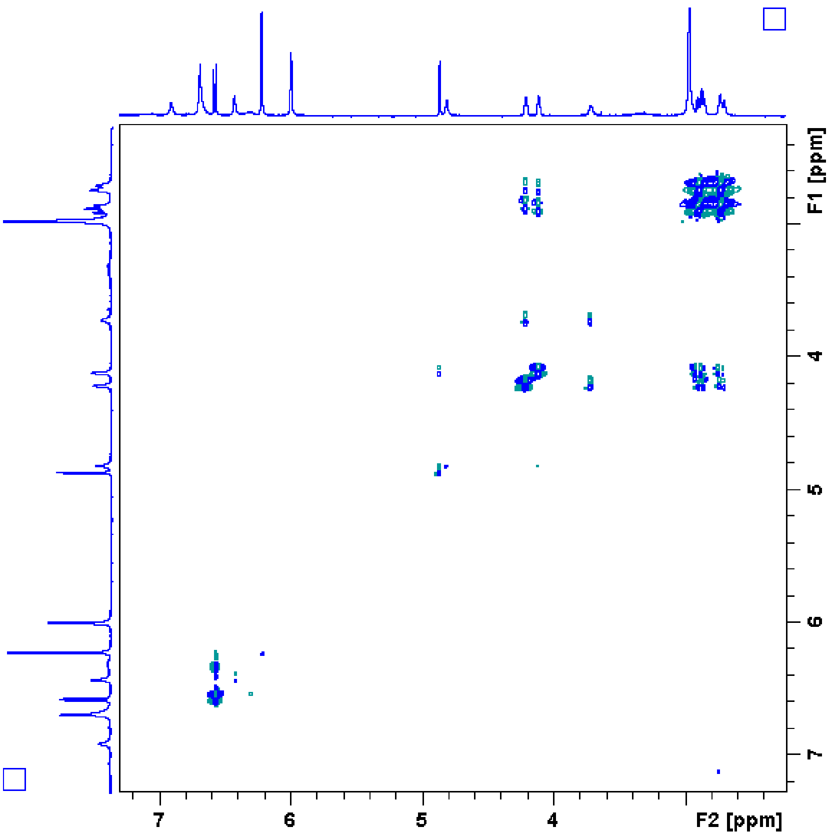

Figure S48. ^{1}H, ^{1}H COSY spectrum of epicatechin–pyrogallolmethine–epicatechin (**10**)

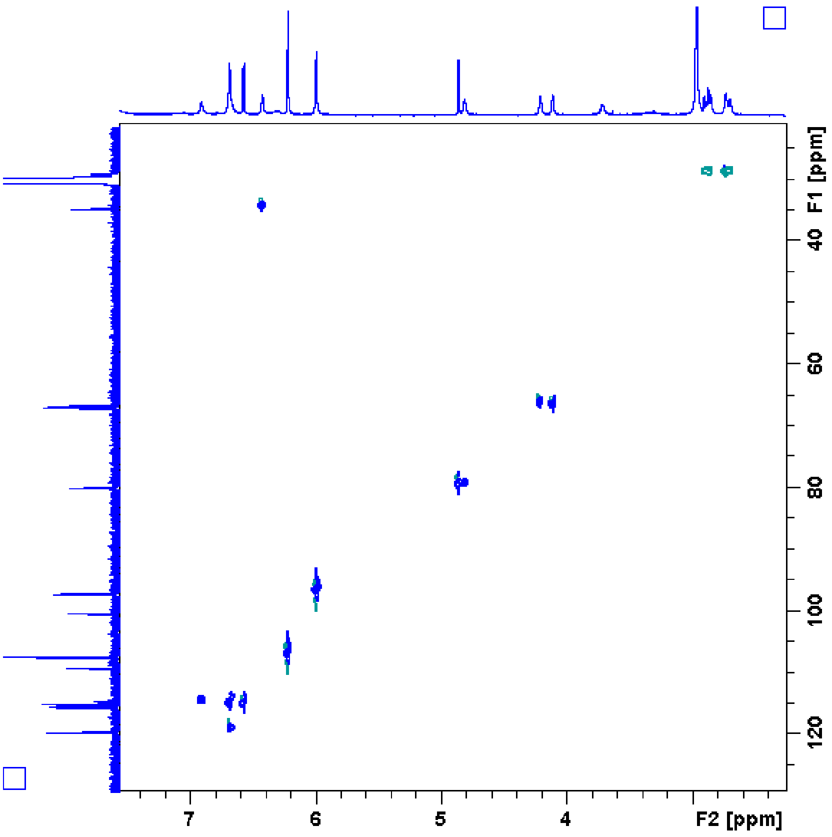

Figure S49. ^{1}H, ^{13}C HSQC spectrum of epicatechin–pyrogallolmethine–epicatechin (**10**)

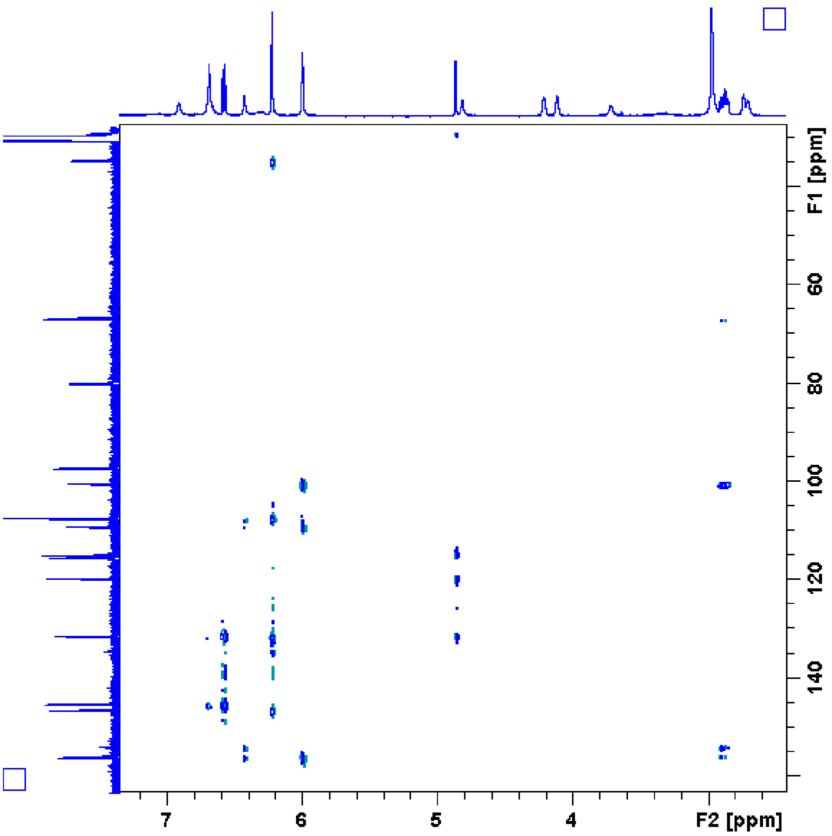

Figure S50. ^{1}H, ^{13}C HMBC spectrum of epicatechin–pyrogallolmethine–epicatechin (**10**)

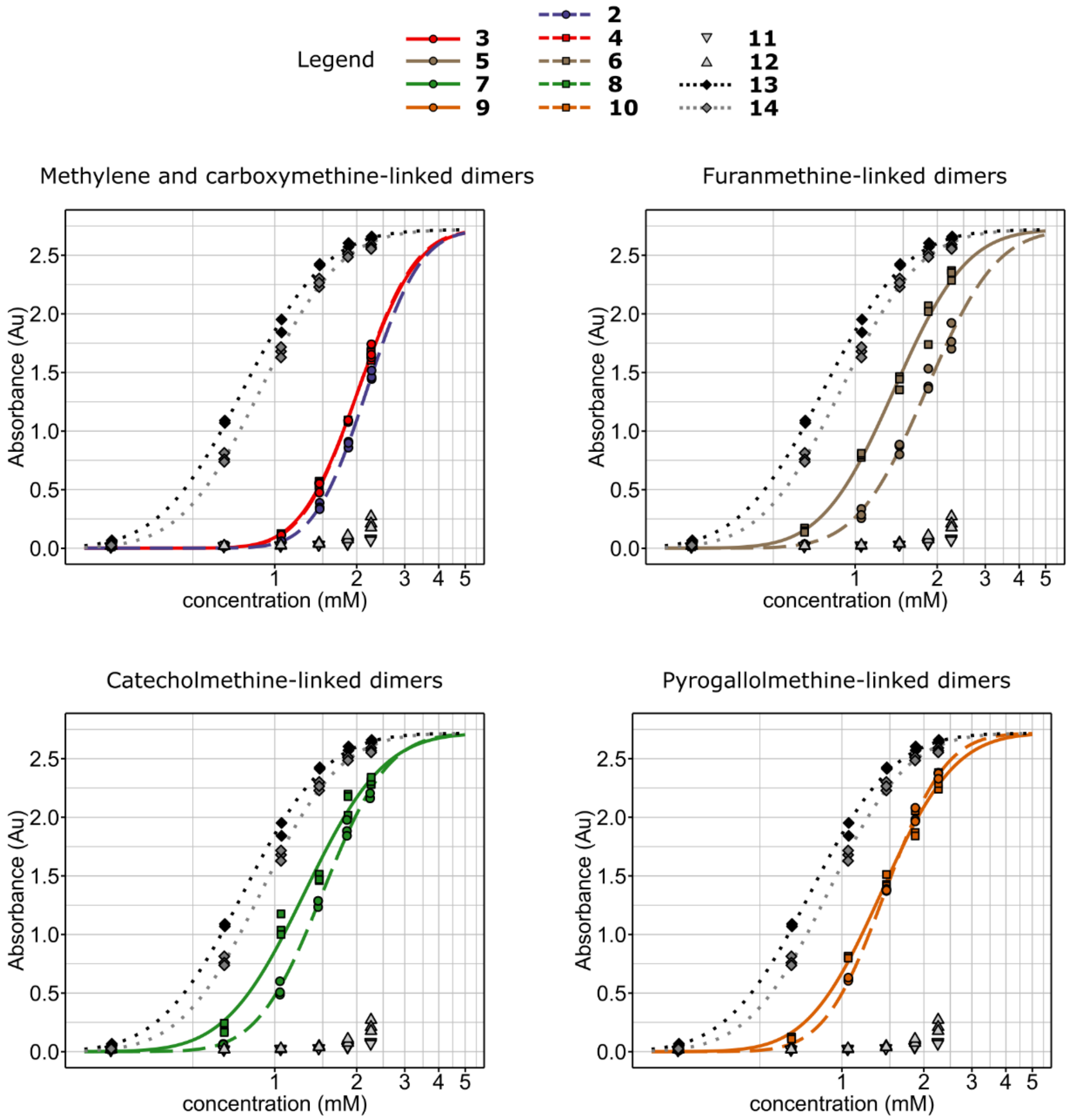

Figure S51. Graphs of the log-normal dose–response model for the protein precipitation capacity by different linkage types. The concentration in the x-axis is the concentration of compounds **2**–**14** in the reaction mixture, while the concentration of BSA was kept at a constant of 0,1 mM. The compounds that had no activity (**15**, **16**), low activity (**11**, **12**) or were not soluble to suitable solvents (**1**) were excluded from the model. Common lower asymptote was assumed to be zero and each compound was assumed to have the same upper asymptote, which was estimated from the data. Refer to Table 1 for the estimates of the parameters.

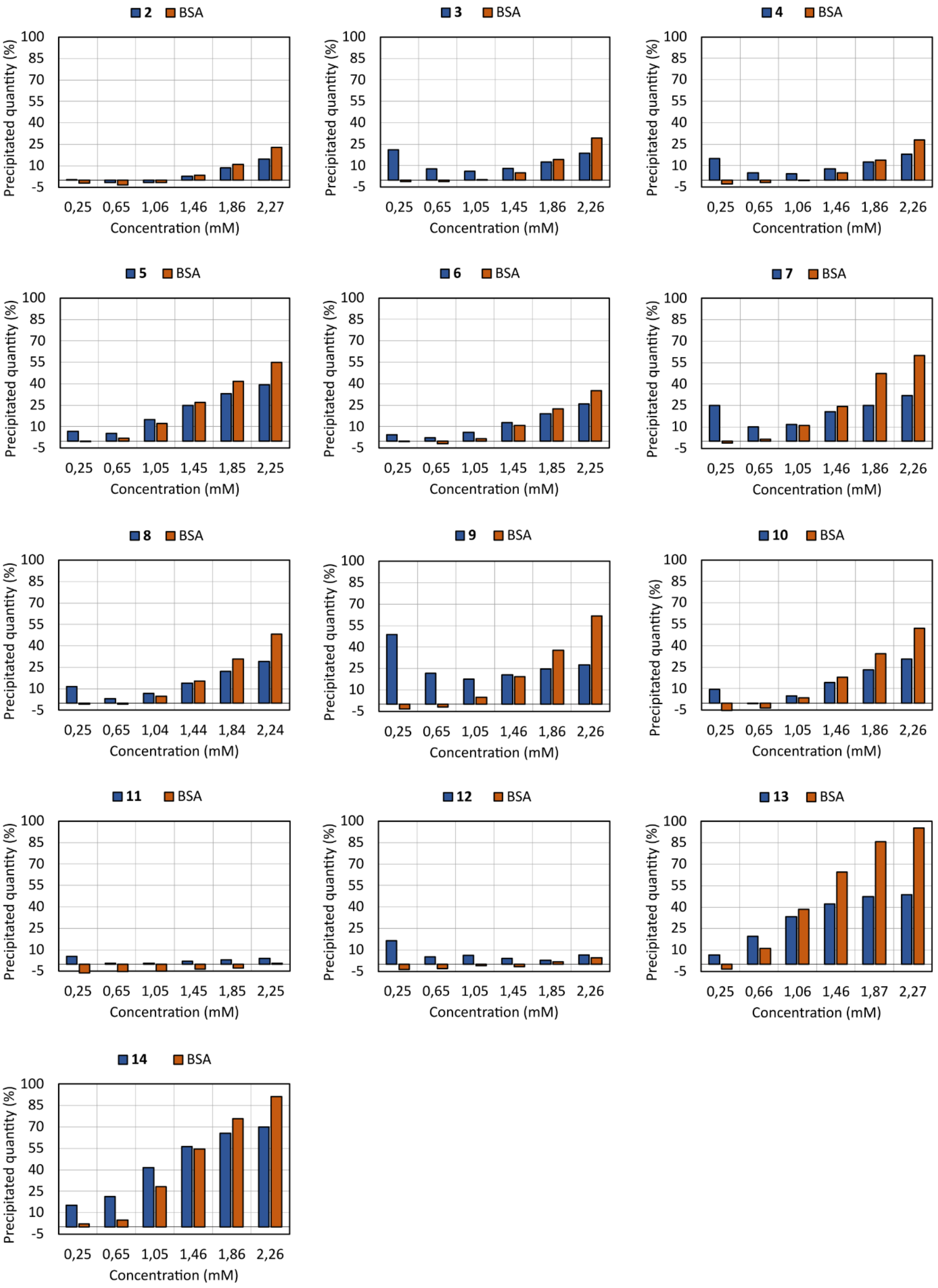

Figure S52. Proportions (%) of the precipitated compounds in the PPC experiment. The precipitated quantity was calculated by subtracting the concentrations of the compounds in the supernatants from the known initial concentrations in the reaction mixtures. The concentrations in the x-axis are the concentrations of compounds **2**–**14** in the reaction mixture. The concentration of BSA was kept at a constant of 0.1 mM.

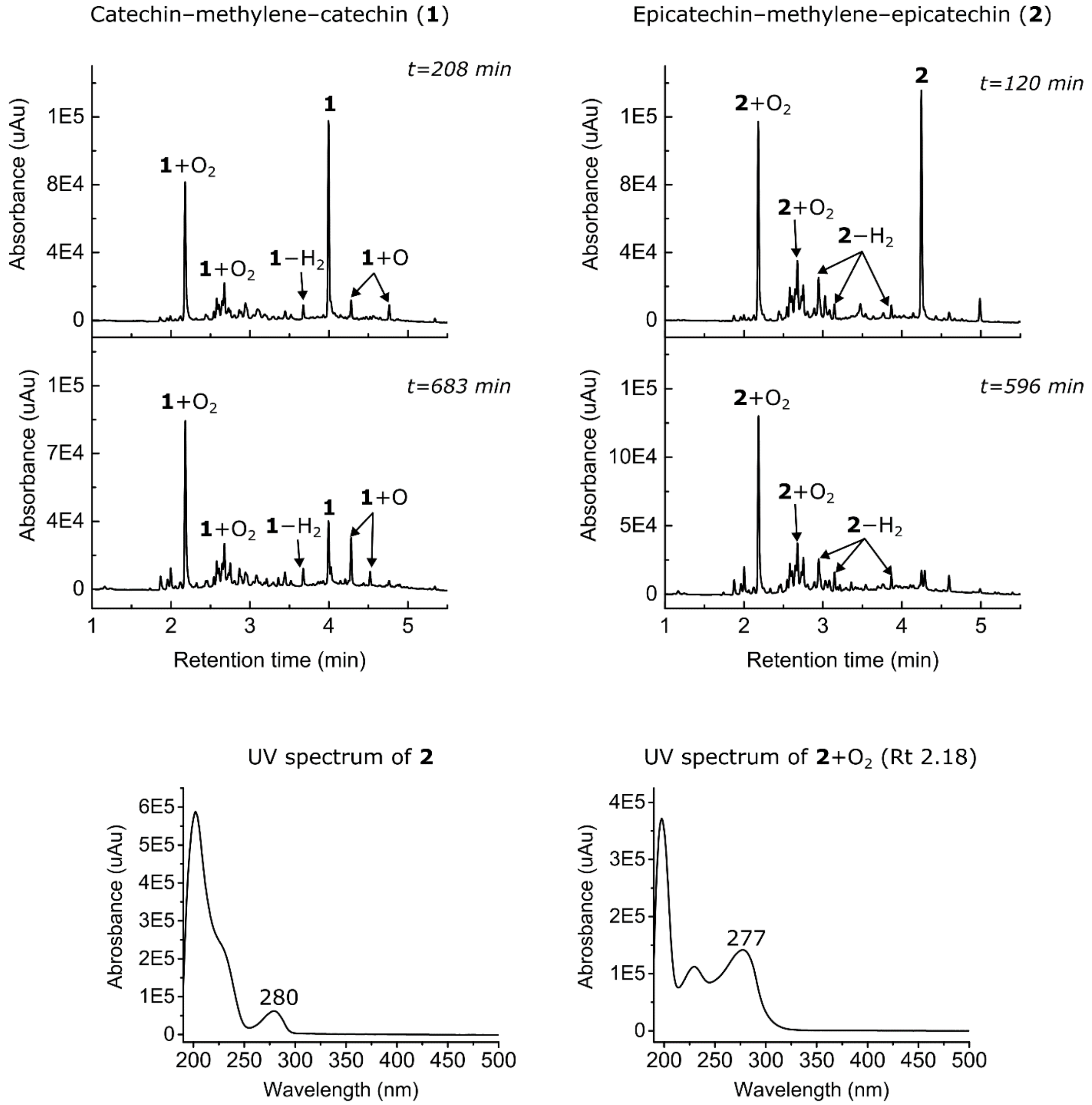

Figure S53. UV chromatograms (280 nm) of compounds **1** and **2** from the stability experiments. Upper chromatograms are at the half-life of the compound and lower ones are at the last time point that was utilized for the kinetics plot (see figure 4), i.e., either at a time point when majority of the starting material had degraded or at the end of the experiments. Selected UV spectra of epicatechin-based dimers are shown as examples. Corresponding UV spectra of **1** were identical to the spectra of **2**.

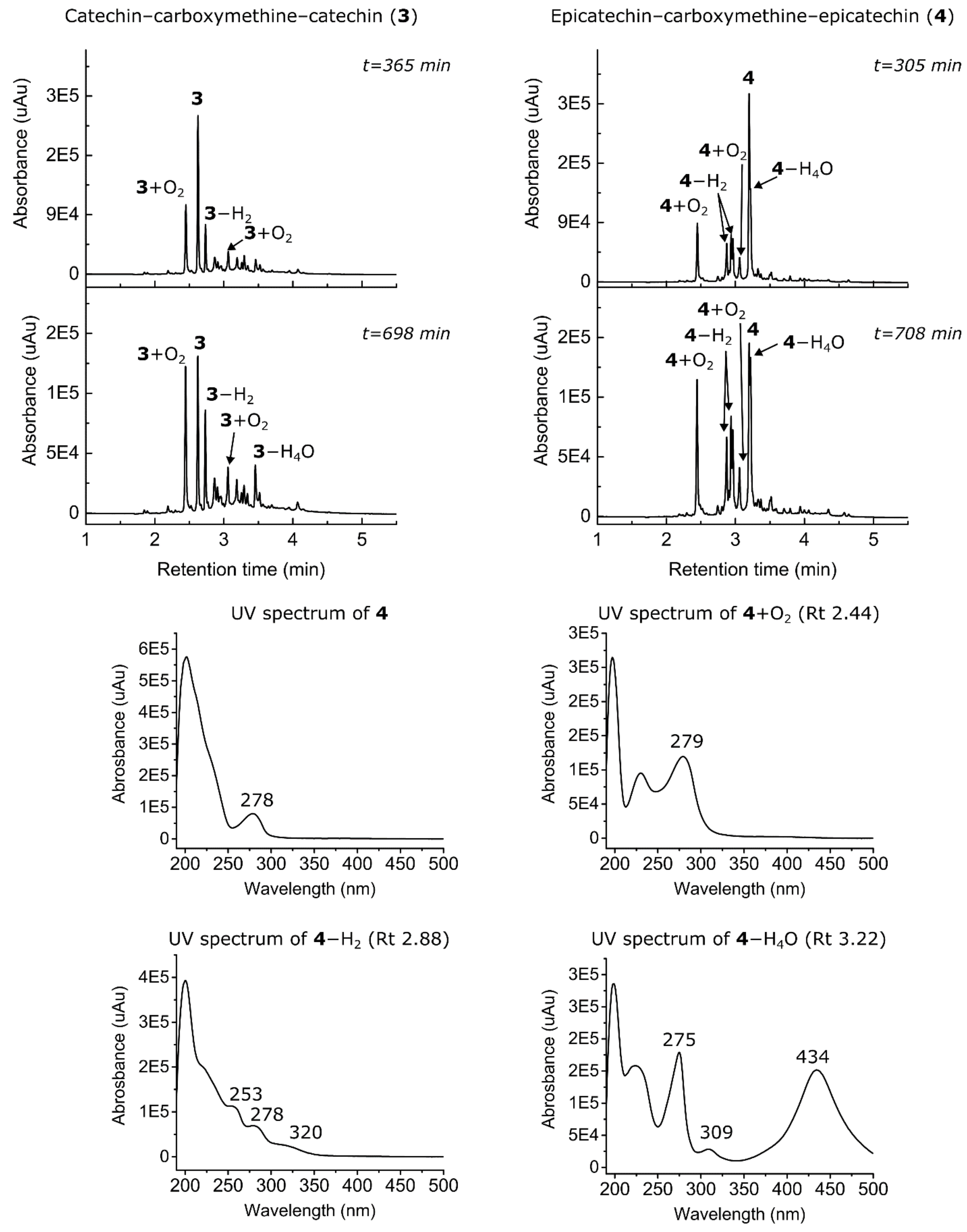

Figure S54. UV chromatograms (280 nm) of compounds **3** and **4** from the stability experiments. Upper chromatograms are at the half time of the compound and lower ones are at the last time point that was utilized for the kinetics plot (see figure 4), i.e., either at a time point when majority of the starting material had degraded or at the end of the experiments. Selected UV spectra of epicatechin-based dimers are shown as examples. Corresponding UV spectra of **3** were identical to the spectra of **4**.

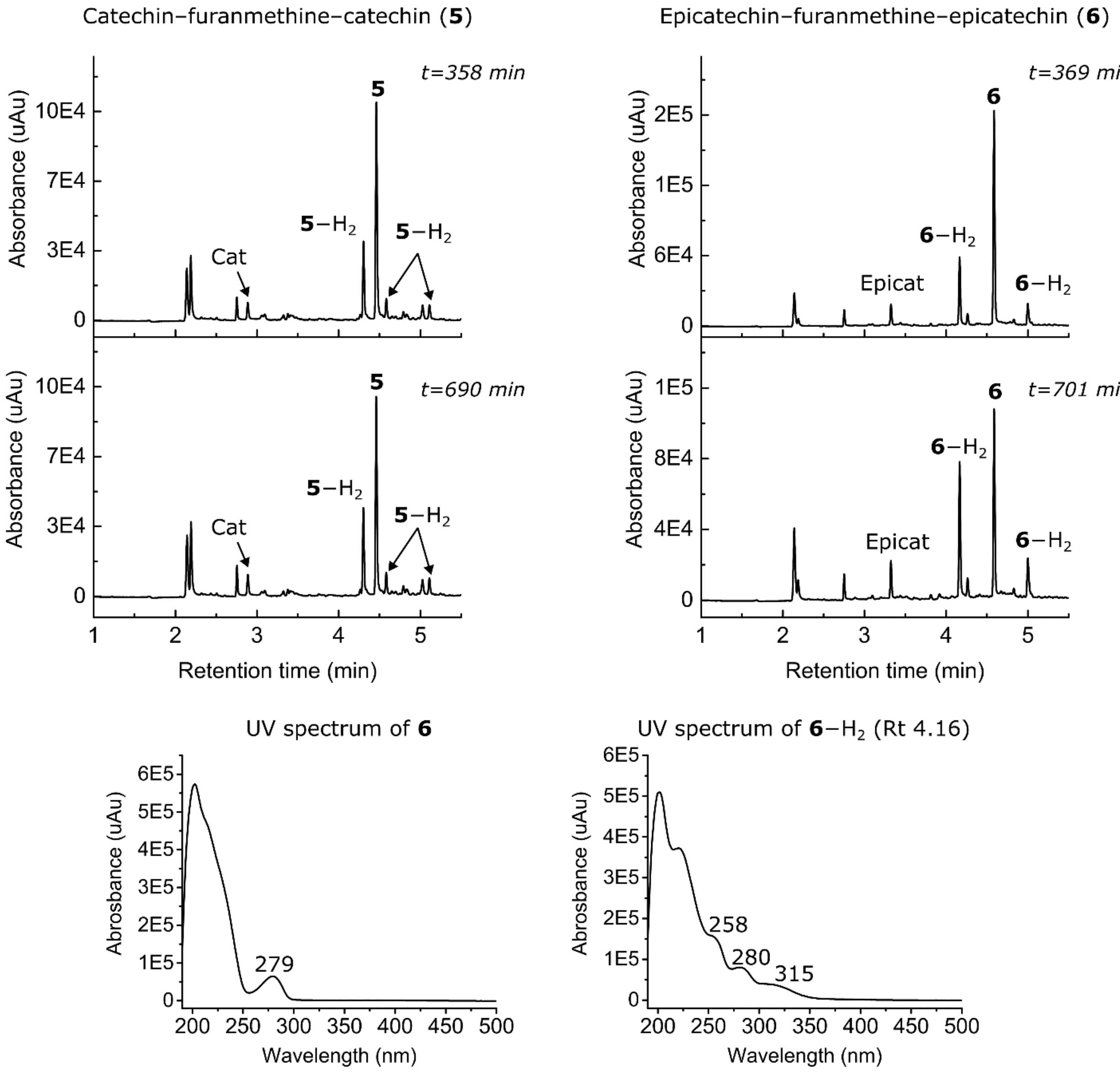

Figure S55. UV chromatograms (280 nm) of compounds **5** and **6** from the stability experiments. Upper chromatograms are at the half-life of the compound and lower ones are at the last time point that was utilized for the kinetics plot (see figure 4), i.e., either at a time point when majority of the starting material had degraded or at the end of the experiments. Selected UV spectra of epicatechin-based dimers are shown as examples. Corresponding UV spectra of **5** were identical to the spectra of **8**.

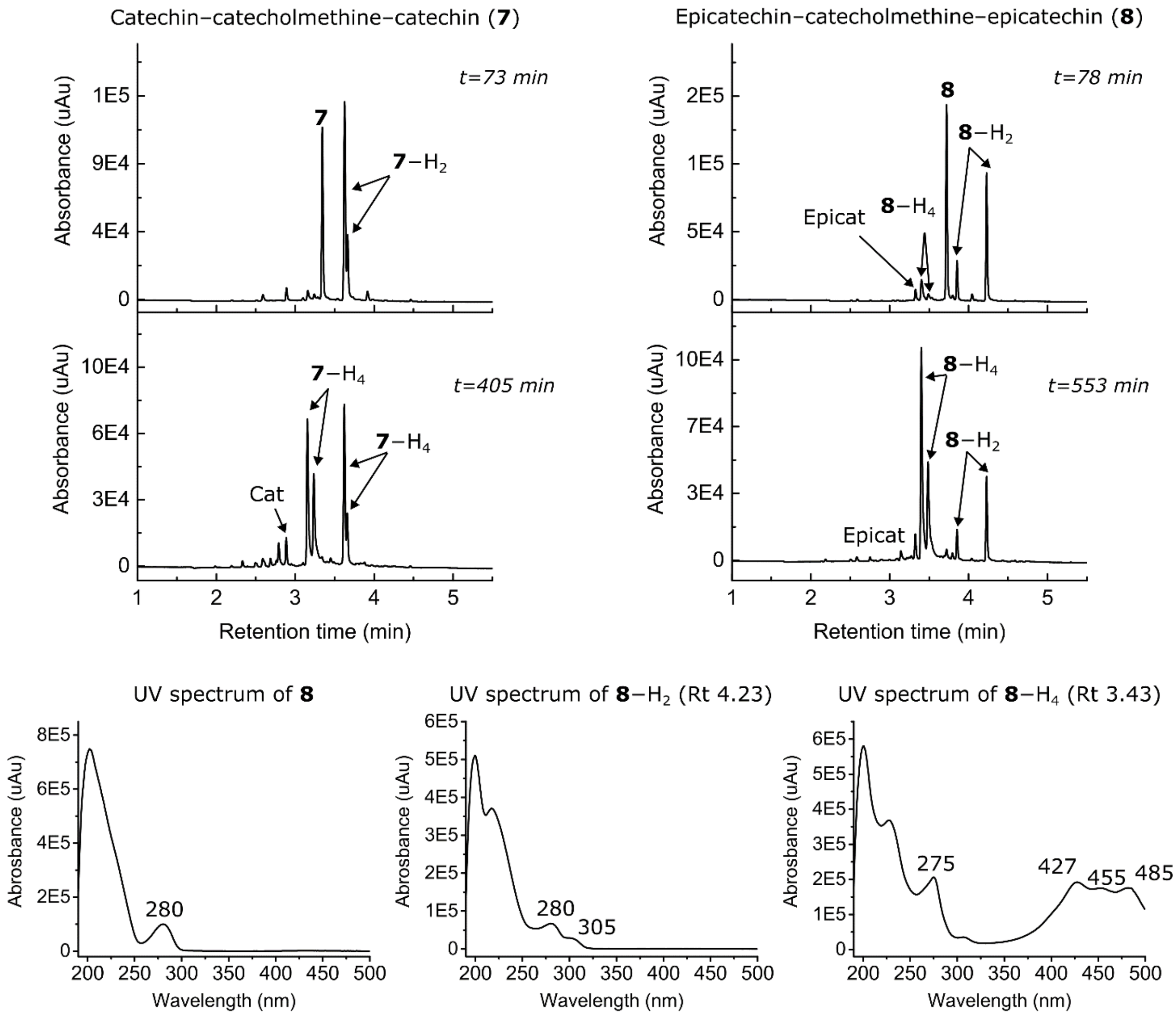

Figure S56. UV chromatograms (280 nm) of compounds **7** and **8** from the stability experiments. Upper chromatograms are at the half-life of the compound and lower ones are at the last time point that was utilized for the kinetics plot (see figure 4), i.e., either at a time point when majority of the starting material had degraded or at the end of the experiments. Selected UV spectra of epicatechin-based dimers are shown as examples. Corresponding UV spectra of **7** were identical to the spectra of **8**.

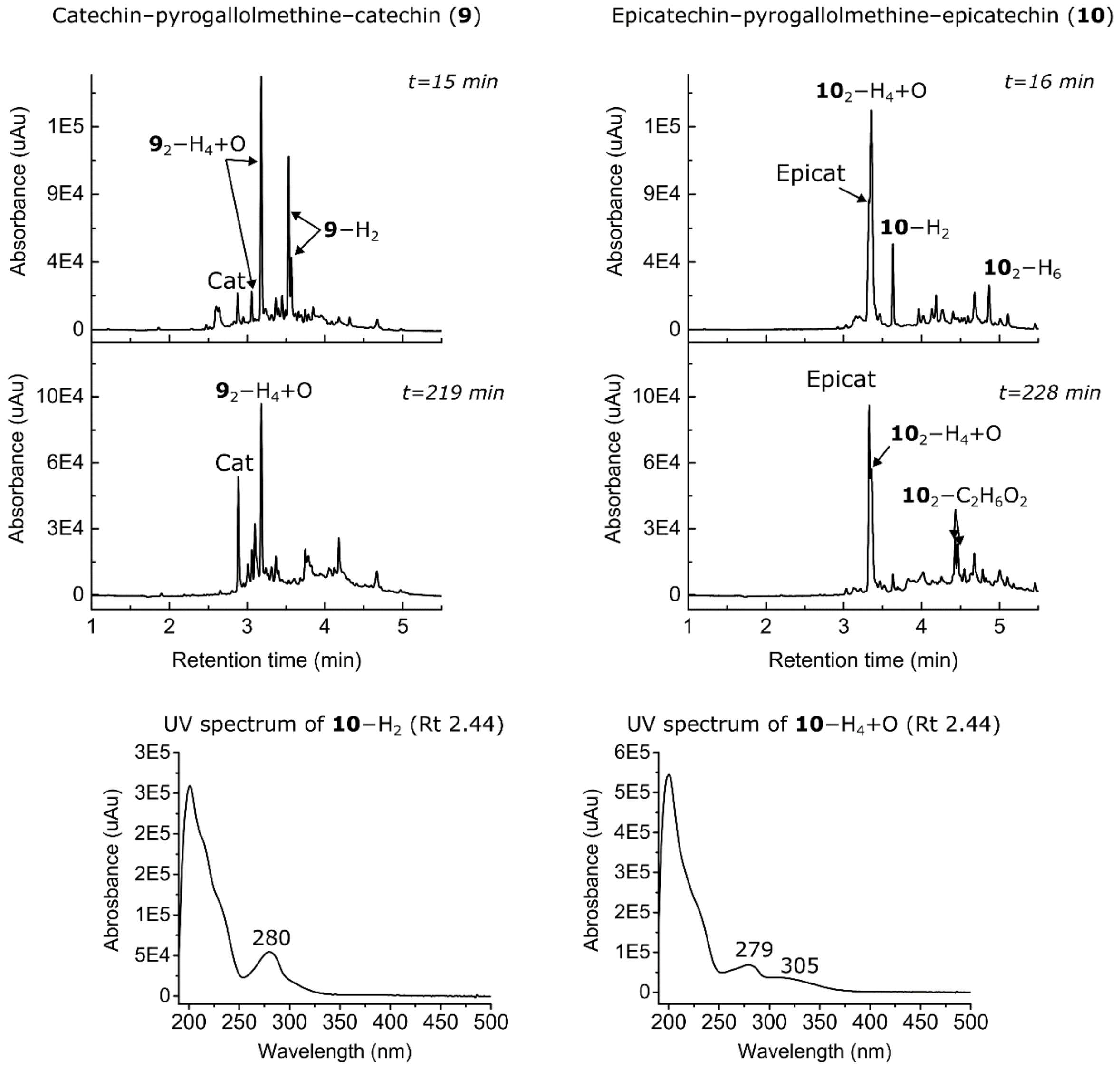

Figure S57. UV chromatograms (280 nm) of compounds **9** and **10** from the stability experiments. The starting materials had degraded completely already at the first time point. Selected UV spectra of epicatechin-based dimers are shown as examples. Corresponding UV spectra of **9** were identical to the spectra of **10**.

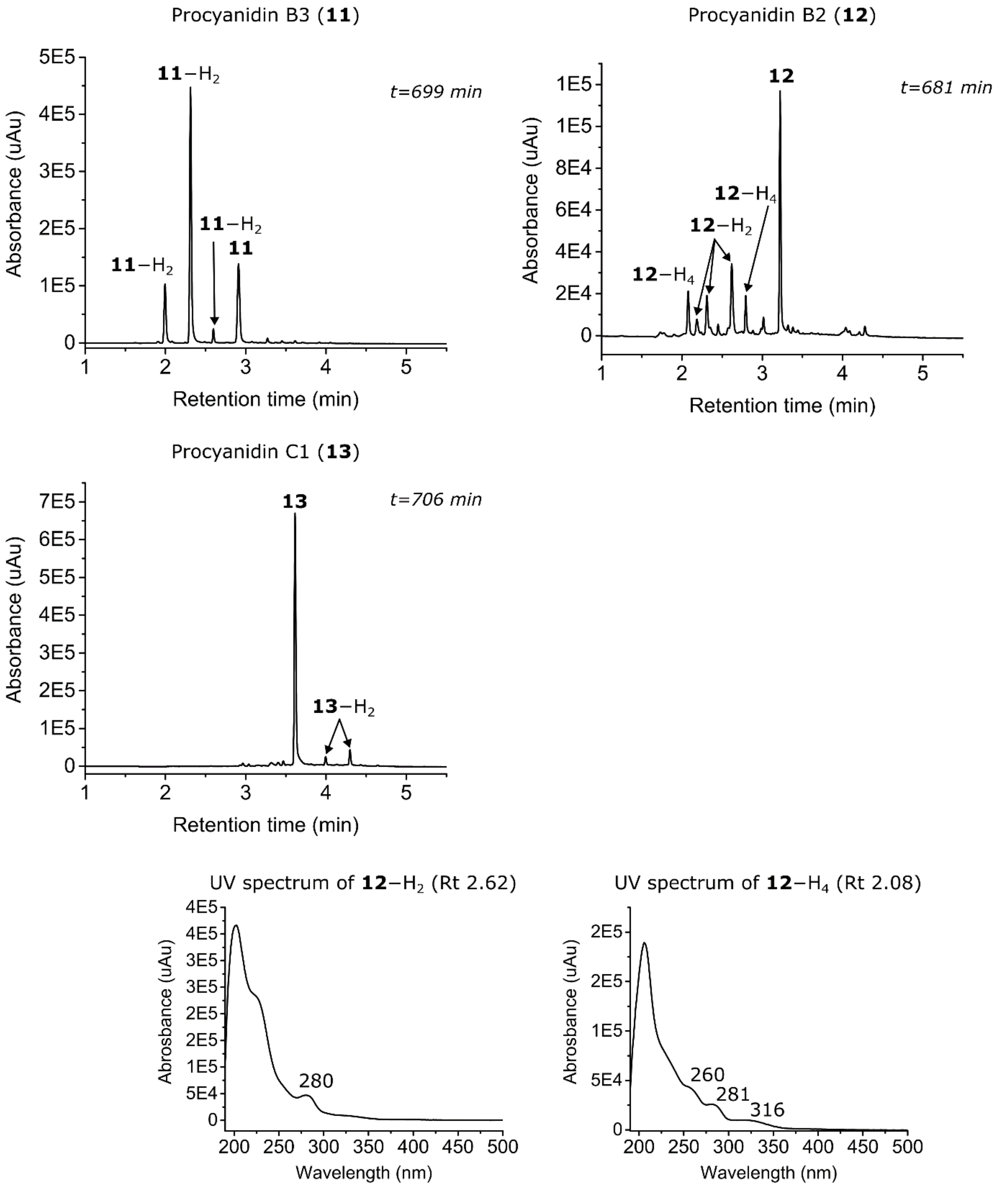

Figure S58. UV chromatograms (280 nm) of compounds **11**–**13** from the stability experiments at the last time point of the experiment (13 hours). Selected UV spectra of epicatechin-based dimers are shown as examples. Corresponding UV spectra of **11** and **13** were identical to the spectra of **12**.

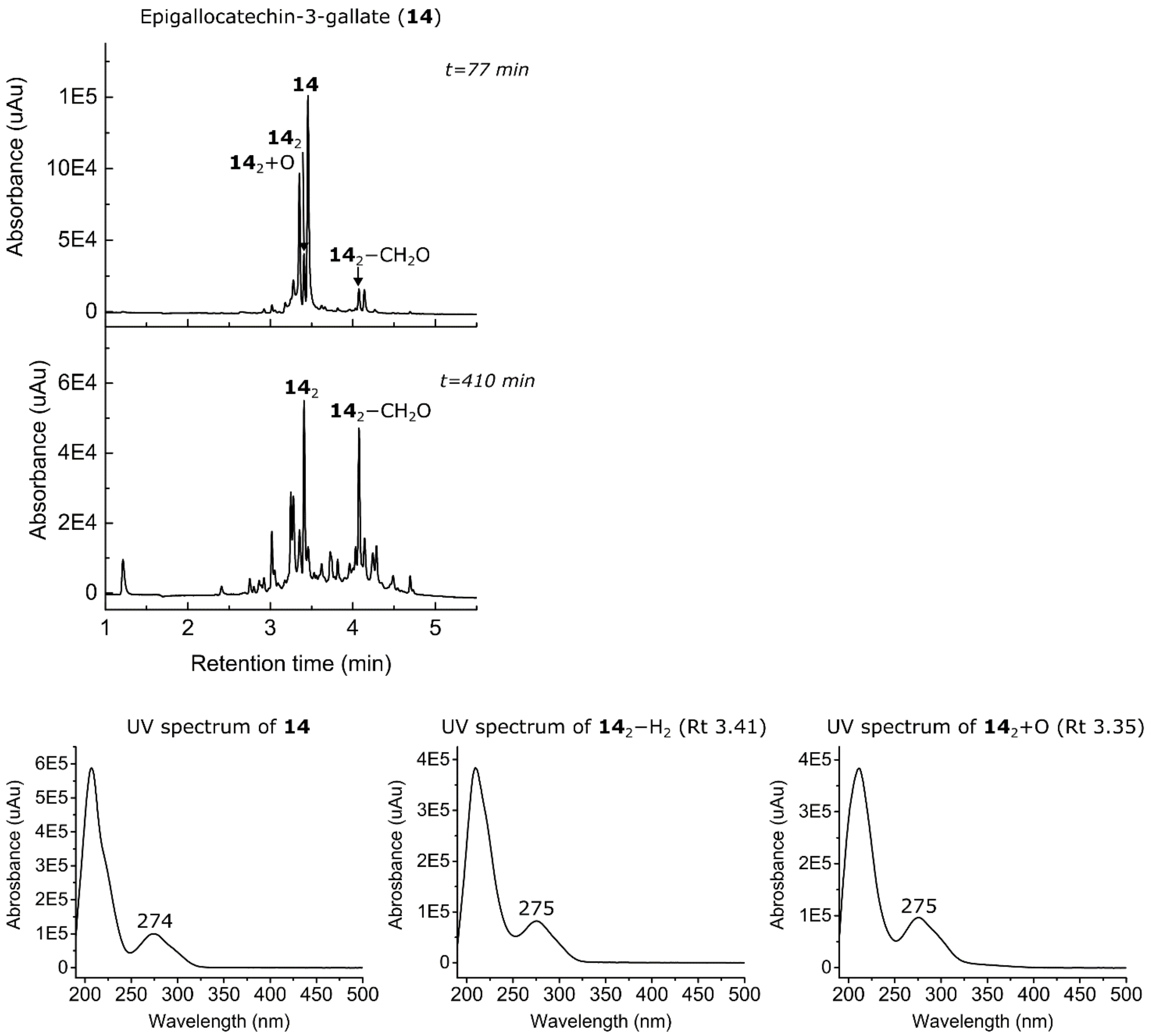

Figure S59. UV chromatograms (280 nm) of compound **14** from the stability experiments. The upper chromatogram of is at the half-life of the compound **14** and the lower is at the last time point that was utilized for the kinetics curves (see Figure 4). Selected UV spectra are shown as examples.

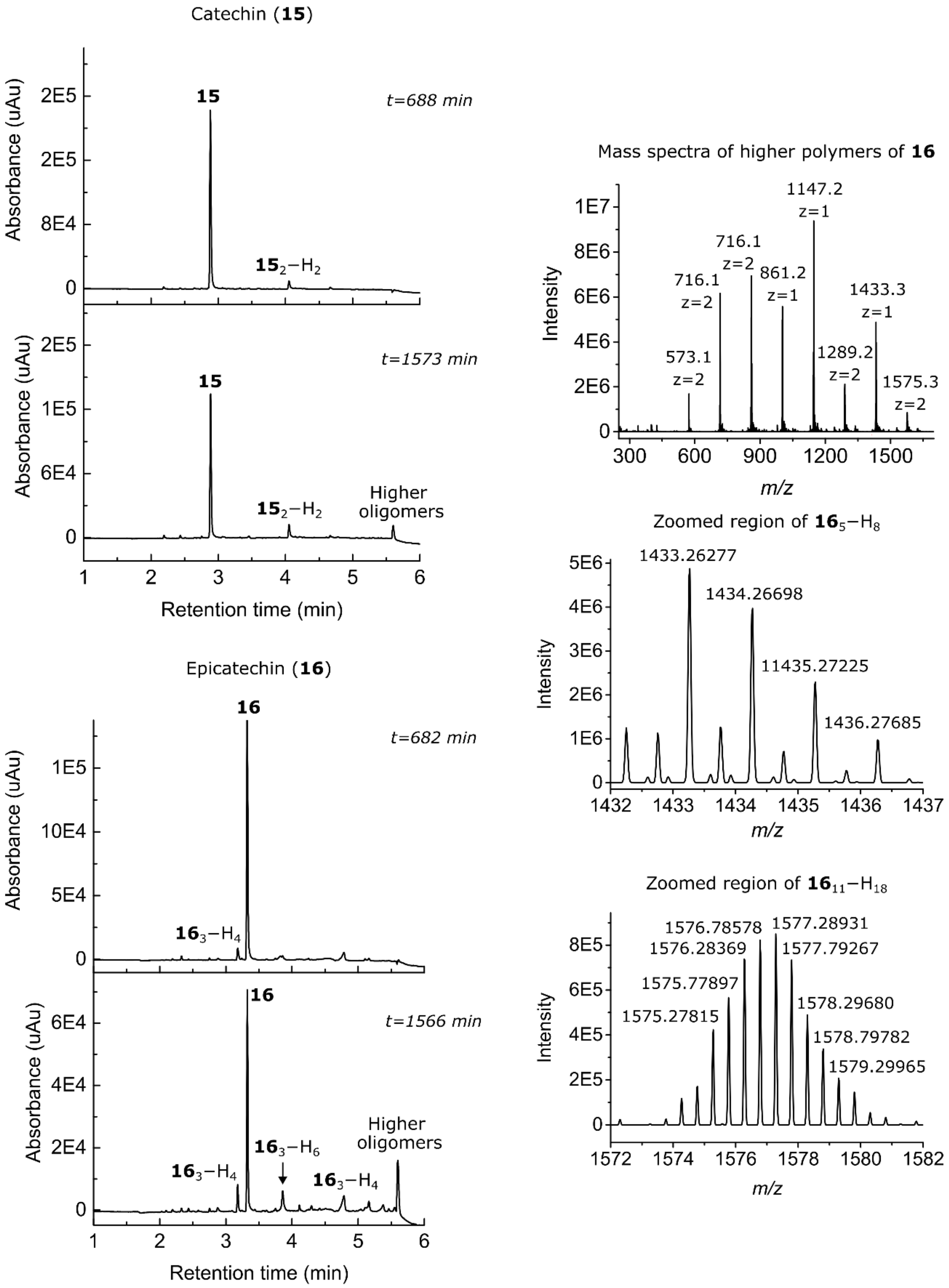

Figure S60. UV chromatograms (280 nm) of compounds **15** and **16** from the stability experiments. The upper chromatograms are at the time point when the experiment was halted with the other compounds (13 hours) but with **15** and **16** the experiment was continued for additional 13 hours (lower chromatograms). Mass spectrum of higher polymers of **16** at 26 hours with two zoomed regions are given as an example of higher polymers of both **15** and **16**.

Table S1. Compound characterization of the products in the stability experiments in phosphate buffered saline solution (Fig. 5 and S53–S60). Only UV maxima above 250 nm are reported. Abbreviations: sh = shoulder. See figures S53–S60 for examples of UV spectra.

Compound identification	Retention time (min)	Measured *m/z* ([M−H]⁻)	Measured mass (Da)	Exact mass (Da)	Mass error (ppm)	Molecular formula	UV maxima (nm)
Epicatechin–methylene–epicatechin (**1**)	3.99	591.15091	592.15819	592.15808	0.180	$C_{31}H_{28}O_{12}$	280
**1**+O_2	2.18	623.14151	624.14879	624.14791	1.404	$C_{31}H_{28}O_{14}$	278
**1**+O_2	2.68	623.14122	624.14850	624.14791	0.940	$C_{31}H_{28}O_{14}$	278
**1**−H_2	3.67	589.13519	590.14247	590.14243	0.062	$C_{31}H_{26}O_{12}$	-
**1**+O	4.28	607.14586	608.15314	608.15300	0.224	$C_{31}H_{28}O_{13}$	282, 305 (sh)
**1**+O	4.52	607.14603	608.15331	608.15300	0.504	$C_{31}H_{28}O_{13}$	-
Epicatechin–methylene–epicatechin (**2**)	4.25	591.15138	592.15866	592.15808	0.973	$C_{31}H_{28}O_{12}$	280
**2**−H_2	2.95	589.13557	590.14285	590.14243	0.706	$C_{31}H_{26}O_{12}$	271
**2**−H_2	3.15	589.13594	590.14322	590.14243	1.333	$C_{31}H_{26}O_{12}$	-
**2**−H_2	3.87	589.13550	590.14278	590.14243	0.587	$C_{31}H_{26}O_{12}$	-
**2**+O_2	2.18	623.14057	624.14785	624.14791	-0.102	$C_{31}H_{28}O_{14}$	277
**2**+O_2	2.68	623.14114	624.14842	624.14791	0.811	$C_{31}H_{28}O_{14}$	276
Epicatechin–carboxymethine–epicatechin (**3**)	2.62	635.14112	636.14840	636.14791	0.765	$C_{32}H_{28}O_{14}$	279
**3** (isomer)	3.29	635.14097	636.14825	636.14791	0.529	$C_{32}H_{28}O_{14}$	278
**3**−H_2	2.73	633.12546	634.13274	634.13226	0.751	$C_{32}H_{26}O_{14}$	253 (sh), 278 (sh)
**3**+O_2	3.06	667.13113	668.13841	668.13774	0.997	$C_{32}H_{28}O_{16}$	278
**3**+O_2	2.45	667.13000	668.13728	668.13774	-0.694	$C_{32}H_{28}O_{16}$	279
**3**−H_4O	3.46	615.11481	616.12209	616.12169	0.643	$C_{32}H_{24}O_{13}$	275, 436
Epicatechin–carboxymethine–epicatechin (**4**)	3.20	635.14050	636.14778	636.14791	-0.210	$C_{32}H_{28}O_{14}$	278
**4** (isomer)	2.96	635.14032	636.14760	636.14791	-0.493	$C_{32}H_{28}O_{14}$	276
**4**−H_2	2.87	633.12542	634.13270	634.13226	0.688	$C_{32}H_{26}O_{14}$	258 (sh), 278, 315 (sh)
**4**−H_2	2.94	633.12560	634.13288	634.13226	0.972	$C_{32}H_{26}O_{14}$	273
**4**+O_2	2.44	667.13087	668.13815	668.13774	0.608	$C_{32}H_{28}O_{16}$	279
**4**+O_2	2.91	667.13070	668.13798	668.13774	0.354	$C_{32}H_{28}O_{16}$	-
**4**+O_2	3.06	667.13118	668.13846	668.13774	1.072	$C_{32}H_{28}O_{16}$	278
**4**−H_4O	3.22	615.11483	616.12211	616.12169	0.676	$C_{32}H_{24}O_{13}$	275, 435

Compound identification	Retention time (min)	Measured m/z ([M–H]$^-$)	Measured mass (Da)	Exact mass (Da)	Mass error (ppm)	Molecular formula	UV maxima (nm)
Catechin–furanmethine–catechin (**5**)	4.46	657.16156	658.16884	658.16864	0.298	$C_{35}H_{30}O_{13}$	280
**5** (isomer)	5.03	657.16191	658.16919	658.16864	0.830	$C_{35}H_{30}O_{13}$	279
**5**–H_2	4.30	655.14593	656.15321	656.15299	0.330	$C_{35}H_{28}O_{13}$	258, 282, 315 (sh)
**5**–H_2	4.58	655.14630	656.15358	656.15299	0.894	$C_{35}H_{28}O_{13}$	-
**5**–H_2	5.11	655.14625	656.15353	656.15299	0.818	$C_{35}H_{28}O_{13}$	-
Catechin	2.91	289.07178	290.07906	290.07904	0.057	$C_{15}H_{14}O_6$	279
Epicatechin–furanmethine–epicatechin (**6**)	4.59	657.16162	658.16890	658.16864	0.390	$C_{35}H_{30}O_{13}$	279
**6**–H_2	4.16	655.14632	656.15360	656.15299	0.924	$C_{35}H_{28}O_{13}$	258 (sh), 280, 315 (sh)
**6**–H_2	5.02	655.14615	656.15343	656.15299	0.665	$C_{35}H_{28}O_{13}$	258 (sh), 280, 315 (sh)
Epicatechin	3.32	289.07183	290.07911	290.07904	0.229	$C_{15}H_{14}O_6$	278
Catechin–catecholmethine–catechin (**7**)	3.37	699.17191	700.17919	700.17920	-0.019	$C_{37}H_{32}O_{14}$	280
**7**–H_2	3.63	697.15361	698.16089	698.16356	-3.829	$C_{37}H_{30}O_{14}$	280, 304 (sh)
**7**–H_2	3.66	697.15613	698.16341	698.16356	-0.220	$C_{37}H_{30}O_{14}$	279, 304 (sh)
**7**–H_4	3.15	695.14093	696.14821	696.14791	0.426	$C_{37}H_{28}O_{14}$	275, 427, 452, 480
**7**–H_4	3.23	695.14097	696.14825	696.14791	0.483	$C_{37}H_{28}O_{14}$	275, 427, 452, 480
Catechin	2.92	289.07170	290.07898	290.07904	-0.219	$C_{15}H_{14}O_6$	279
Epicatechin–catecholmethine–epicatechin (**8**)	3.75	699.17184	700.17912	700.17920	-0.119	$C_{37}H_{32}O_{14}$	280
**8**–H_2	3.85	697.15644	698.16372	698.16356	0.224	$C_{37}H_{30}O_{14}$	280, 304 (sh)
**8**–H_2	4.23	697.15618	698.16346	698.16356	-0.148	$C_{37}H_{30}O_{14}$	280, 304 (sh)
**8**–H_4	3.43	695.14050	696.14778	696.14791	-0.192	$C_{37}H_{28}O_{14}$	275, 428, 452, 482
**8**–H_4	3.51	695.14043	696.14771	696.14791	-0.292	$C_{37}H_{28}O_{14}$	275, 427, 455, 485
Epicatechin	3.32	289.07163	290.07891	290.07904	-0.460	$C_{15}H_{14}O_6$	278
**9**–H_2	3.53	713.15160	714.15888	714.15847	0.569	$C_{37}H_{30}O_{15}$	280, 310
**9**–H_2	3.57	713.15200	714.15928	714.15847	1.129	$C_{37}H_{30}O_{15}$	280, 310
**9**$_2$–H_4+O	3.18	1441.28958	1442.29686	1442.29622	0.441	$C_{74}H_{58}O_{31}$	280, 320 (sh)
**9**$_2$–H_4+O	3.06	1441.28772	1442.29500	1442.29622	-0.848	$C_{74}H_{58}O_{31}$	280, 320 (sh)
Catechin	2.90	289.07171	290.07899	290.07904	-0.185	$C_{15}H_{14}O_6$	280

Compound identification	Retention time (min)	Measured *m/z* ([M−H]⁻)	Measured mass (Da)	Exact mass (Da)	Mass error (ppm)	Molecular formula	UV maxima (nm)
**10**$_2$−H_4+O	3.35	1441.28845	1442.29573	1442.29622	-0.342	$C_{74}H_{58}O_{31}$	278, 320 (sh)
**10**−H_2	3.63	713.15103	714.15831	714.15847	-0.229	$C_{37}H_{30}O_{15}$	280, 305 (t)
**10**$_2$−$C_2H_6O_2$	4.68	1367.28735	1368.29463	1368.29528	-0.478	$C_{72}H_{56}O_{28}$	-
**10**$_2$−H_6	4.87	1423.27741	1424.28469	1424.28565	-0.677	$C_{74}H_{56}O_{30}$	277, 320 (sh)
**10**$_2$−$C_2H_6O_2$	4.43	1367.28805	1368.29533	1368.29528	0.034	$C_{72}H_{56}O_{28}$	279, 325 (sh)
**10**$_2$−$C_2H_6O_2$	4.47	1367.28739	1368.29467	1368.29528	-0.448	$C_{72}H_{56}O_{28}$	278, 325 (sh)
Epicatechin	3.32	289.07166	290.07894	290.07904	-0.357	$C_{15}H_{14}O_6$	279
PC B3 (**11**)	2.93	577.13593	578.14321	578.14243	1.343	$C_{30}H_{26}O_{12}$	279
**11**−H_2	2.00	575.12031	576.12759	576.12678	1.400	$C_{30}H_{24}O_{12}$	281
**11**−H_2	2.31	575.12017	576.12745	576.12678	1.157	$C_{30}H_{24}O_{12}$	280
**11**−H_2	2.60	575.12054	576.12782	576.12678	1.799	$C_{30}H_{24}O_{12}$	-
**11**−H_2	3.27	575.12030	576.12758	576.12678	1.382	$C_{30}H_{24}O_{12}$	-
PC B2 (**12**)	3.22	577.13500	578.14228	578.14243	-0.266	$C_{30}H_{26}O_{12}$	279
**12**−H_2	2.19	575.11979	576.12707	576.12678	0.497	$C_{30}H_{24}O_{12}$	280
**12**−H_2	2.31	575.11982	576.12710	576.12678	0.549	$C_{30}H_{24}O_{12}$	280
**12**−H_2	2.62	575.11989	576.12717	576.12678	0.671	$C_{30}H_{24}O_{12}$	280, 330
**12**−H_4	2.08	573.10416	574.11144	574.11113	0.534	$C_{30}H_{22}O_{12}$	258 (sh), 280, 325
**12**−H_4	2.79	573.10428	574.11156	574.11113	0.743	$C_{30}H_{22}O_{12}$	281
PC C1 (**13**)	3.61	865.19937	866.20665	866.20581	0.966	$C_{45}H_{38}O_{18}$	279
**13**−H_2	3.99	863.18458	864.19186	864.19016	1.963	$C_{45}H_{36}O_{18}$	278
**13**−H_2	4.30	863.18399	864.19127	864.19016	1.280	$C_{45}H_{36}O_{18}$	279
Epigallocatechin-3-gallate (**14**)	3.46	457.07817	458.08545	458.08491	1.171	$C_{22}H_{18}O_{11}$	274
**14**$_2$ (Theasinesin)	3.41	913.14798	914.15526	914.15417	1.188	$C_{44}H_{34}O_{22}$	275
**14**$_2$−CH_2O (Theasinensin P-2)	4.07	883.13782	884.14510	884.14362	1.670	$C_{43}H_{32}O_{21}$	-
**14**$_2$+O	3.35	929.14269	930.14997	930.14910	0.931	$C_{44}H_{34}O_{23}$	275
**15**$_2$−H_2	4.05	575.12009	576.12737	576.12678	1.018	$C_{30}H_{24}O_{12}$	278, 414
**15**$_3$−H_4	5.60	861.16724	862.17452	862.17451	0.007	$C_{45}H_{34}O_{18}$	257 (sh), 368
**15**$_4$−H_6	5.60	1147.21598	1148.22326	1148.22225	0.877	$C_{60}H_{44}O_{24}$	257 (sh), 368
**15**$_5$−H_8	5.60	1433.26260	1434.26988	1434.26999	-0.079	$C_{75}H_{54}O_{30}$	257 (sh), 368
**15**$_6$−H_{10}	5.60	859.15308[a]	1720.32071	1720.31773	1.734	$C_{90}H_{64}O_{36}$	257 (sh), 368

Compound identification	Retention time (min)	Measured *m/z* ([M−H]$^-$)	Measured mass (Da)	Exact mass (Da)	Mass error (ppm)	Molecular formula	UV maxima (nm)
**15**$_7$–H_{10}	5.60	1003.18367[a]	2008.38189	2008.38112	0.385	$C_{105}H_{76}O_{42}$	257 (sh), 368
**15**$_8$–H_{12}	5.60	1146.20580[a]	2294.42615	2294.42885	-1.176	$C_{120}H_{86}O_{48}$	257 (sh), 368
**15**$_9$–H_{12}	5.60	1290.23745[a]	2582.48945	2582.49244	-1.157	$C_{135}H_{98}O_{54}$	257 (sh), 368
**16**$_3$–H_4	3.18	861.16864	862.17592	862.17451	1.631	$C_{45}H_{34}O_{18}$	280, 405
**16**$_3$–H_6	3.86	859.15315	860.16043	860.15866	2.054	$C_{45}H_{32}O_{18}$	280, 405
**16**$_3$–H_4	4.79	861.16784	862.17512	862.17451	0.703	$C_{45}H_{34}O_{18}$	375
**16**$_4$–H_6	5.60	1147.21483	1148.22211	1148.22225	-0.125	$C_{60}H_{44}O_{24}$	370
**16**$_5$–H_8	5.60	1433.26291	1434.27019	1434.26999	0.137	$C_{75}H_{54}O_{30}$	370
**16**$_6$–H_{10}	5.60	859.15322[a]	1720.32099	1720.31773	1.897	$C_{90}H_{64}O_{36}$	370
**16**$_7$–H_{12}	5.60	1002.17731[a]	2006.36917	2006.36547	1.846	$C_{105}H_{74}O_{42}$	370
**16**$_8$–H_{12}	5.60	1146.20642[a]	2294.42739	2294.42885	-0.635	$C_{120}H_{86}O_{48}$	370
**16**$_9$–H_{14}	5.60	1289.22951[a]	2580.47357	2580.47659	-1.169	$C_{135}H_{96}O_{54}$	370
**16**$_{10}$–H_{16}	5.60	1432.25295[a]	2866.52045	2866.52433	-1.353	$C_{150}H_{106}O_{60}$	370
**16**$_{11}$–H_{18}	5.60	1575.27820[a]	3152.57095	3152.57207	-0.354	$C_{165}H_{116}O_{66}$	370

[a]Doubly charged $[M{-}2H]^{2-}$ ions.
